# High *PKCλ* expression is required for *ALDH1*-positive cancer stem cell function and indicates a poor clinical outcome in late-stage breast cancer patients

**DOI:** 10.1371/journal.pone.0235747

**Published:** 2020-07-13

**Authors:** Yuka Nozaki, Hitomi Motomura, Shoma Tamori, Yumiko Kimura, Chotaro Onaga, Shotaro Kanai, Yuka Ishihara, Ayaka Ozaki, Yasushi Hara, Yohsuke Harada, Yasunari Mano, Tsugumichi Sato, Keiko Sato, Kazunori Sasaki, Hitoshi Ishiguro, Shigeo Ohno, Kazunori Akimoto

**Affiliations:** 1 Faculty of Pharmaceutical Sciences, Tokyo University of Science, Chiba, Japan; 2 Translational Research Center, Research Institute for Science & Technology, Tokyo University of Science, Chiba, Japan; 3 Research Institute for Biomedical Sciences, Tokyo University of Science, Chiba, Japan; 4 Faculty of Science and technology, Tokyo University of Science, Chiba, Japan; 5 Department of Molecular Biology, Yokohama City University Graduate School of Medical Science, Kanagawa, Japan; 6 Department of Urology, Yokohama City University Graduate School of Medicine, Kanagawa, Japan; 7 Photocatalyst Group, Kanagawa Institute of Industrial Science and Technology, Kanagawa, Japan; Università degli Studi della Campania, ITALY

## Abstract

Despite development of markers for identification of cancer stem cells, the mechanism underlying the survival and division of cancer stem cells in breast cancer remains unclear. Here we report that *PKCλ* expression was enriched in basal-like breast cancer, among breast cancer subtypes, and was correlated with *ALDH1A3* expression (*p* = 0.016, χ^2^-test). Late stage breast cancer patients expressing *PKCλ*^high^ and *ALDH1A3*^high^ had poorer disease-specific survival than those expressing *PKCλ*^low^ and *ALDH1A3*^low^ (*p* = 0.018, log rank test for Kaplan-Meier survival curves: hazard ratio 2.58, 95% CI 1.24–5.37, *p* = 0.011, multivariate Cox regression analysis). Functional inhibition of PKCλ through siRNA-mediated knockdown or CRISPR-Cas9-mediated knockout in ALDH1^high^ MDA-MB 157 and MDA-MB 468 basal-like breast cancer cells led to increases in the numbers of trypan blue-positive and active-caspase 3-positive cells, as well as suppression of tumor-sphere formation and cell migration. Furthermore, the amount of *CASP3* and *PARP* mRNA and the level of cleaved caspase-3 protein were enhanced in PKCλ-deficient ALDH1^high^ cells. An Apoptosis inhibitor (z-VAD-FMK) suppressed the enhancement of cell death as well as the levels of cleaved caspase-3 protein in PKCλ deficient ALDH1^high^ cells. It also altered the asymmetric/symmetric distribution ratio of ALDH1A3 protein. In addition, PKCλ knockdown led to increases in cellular ROS levels in ALDH1^high^ cells. These results suggest that PKCλ is essential for cancer cell survival and migration, tumorigenesis, the asymmetric distribution of ALDH1A3 protein among cancer cells, and the maintenance of low ROS levels in ALDH1-positive breast cancer stem cells. This makes it a key contributor to the poorer prognosis seen in late-stage breast cancer patients.

## Introduction

In numerous countries, breast cancer is the most common malignant neoplasm in women. Breast cancer is classified into at least six subtypes, normal-like, luminal A, luminal B, HER2-enriched, claudin-low, and basal-like, based on stringent patterns of gene expression [1–3]. Among those, basal-like breast cancer, which exhibits stem-like properties and accounts for up to 15–20% of all breast cancers, is associated with particularly poor outcomes [4]. In addition, based on their immunohistochemically determined receptor status, breast cancers have also been classified as ER and/or PgR positive type, HER2 positive type, and triple negative type (TNBC; ER and/or PgR negative, HER2 negative). About 70% of basal-like breast cancers overlap with TNBC [5–8].

Overall, breast cancer prognoses are good. However, patients with late-stage lesions (stage III or IV) have significantly shorter overall survival (OS) [9]. This is because late-stage breast cancer is often resistant to standard medical treatments such as conventional surgery, chemotherapy and radiotherapy, which makes their recurrence and metastasis much more likely [9]. Thus, new pharmacological approaches to manage late-stage cancer are needed.

Cancer stem cells (CSCs) are a small subpopulation of cancer cells exhibiting capacities for self-renewal, multipotency, and tumorigenesis. Apart from those features, CSCs also exhibit characteristic cellular properties, including cell migration, asymmetric cell division and resistance to reactive oxygen species (ROS), and most are resistant to standard chemotherapy and radiotherapy [10–16]. For example, CSCs make up the metastatic niche and generate bulk tumor at distant organs. It is therefore thought that CSCs are a critical factor in the metastatic cascade [12]. CD44^+^/CD24^-/low^ CSCs, derived from breast cancer, exhibit migration potential that increases with tumor grade [13], while a human CD44^+^ CD24^-/low^ Lin^-^ and mouse Thy1^+^ CD24^+^ Lin^-^ CSC-enriched population exhibits low ROS levels and high expression of anti-ROS genes [14].

CSCs have a capacity for asymmetric propagation; that is, they have the ability to generate other stem-like cells or differentiated cells [15]. This feature is controlled by the balance of their symmetric and asymmetric cell division. Cancer cells positive for PKH26 (PKH^pos^) have the capacity for asymmetric division and correlate with poorly differentiated cancers displaying a higher CSC content [16]. A detailed understanding of the mechanisms that define this property of CSCs could potentially reveal novel therapeutic targets and foster progress toward new drug development against CSCs.

CD44^+^/CD24^−/low^, CD133, and ALDH1 are three currently known CSC markers for isolation CSCs from other cancer cells [17–23]. ALDH1, an enzyme that converts aldehydes to carboxylic acids, is abundant in normal stem/progenitor cells and also exhibits higher activity in CSCs from various cancers, including breast cancer [22, 23]. Cells exhibiting higher ALDH1 activity (ALDH1^high^), which are enriched among CSCs, can be isolated using ALDEFLUOR assays with flow cytometry [23]. Moreover, several studies have shown that two ALDH1 isoforms, ALDH1A1 and ALDH1A3, are useful markers for isolating and tracking CSCs [24–26]. In basal-like breast cancers, ALDH1A3 expression predominates, and expression of ALDH1A3, but not ALDH1A1, correlates significantly with cancer type, tumor grade and metastasis in breast cancer [27–32]. However, the mechanism by which basal-like breast CSCs survive and divide remains unclear.

PKCλ is a member of the atypical protein kinase C (aPKC) family, which is a Ser/Thr kinase subfamily of PKC [33, 34]. Mammalian aPKCs include PKCλ and PKCζ, which play critical roles in determining cell polarity in the context of multicellular organisms. For example, they determine the formation and maintenance of the apico-basal polarity of epithelial cells and mediate asymmetric cell division [34–36]. Overexpression of PKCλ has been detected in several cancers, and patients overexpressing PKCλ have poor clinical outcomes [37–48]. In particular, PKCλ is highly expressed in TNBC, where it reportedly mediates cell proliferation, migration, and survival [37]. PKCλ also controls the Notch signaling pathway, a key driver of stemness in KRAS-mediated lung adenocarcinoma [49] and in glioblastoma [50]. In lung adenocarcinoma, PKCλ-NOTCH3 signaling controls tumor-initiating cells (TICs), which exhibit such CSC-like properties as oncosphere formation and an asymmetric CD133 distribution during cell division [49]. In addition, PKCλ controls signaling by SOX2-hedgehog acyl transferase (HHAT), a master transcriptional regulator of stemness, in lung squamous cell carcinoma [51], and Auranofin, a Par6-PKCλ complex inhibitor, suppresses oncosphere growth from ovarian TICs [52]. However, the actions of PKCλ in ALDH1-positive breast CSCs remains largely unexplored.

In the present study, we show that in patients with stage III-IV tumors, high expression of *PKCλ* and *ALDH1A3* contributes to poor clinical outcomes. Furthermore, PKCλ is involved in the regulation of the asymmetric distribution of ALDH1A3 among cells and the maintenance of lower ROS levels in ALDH1-positive breast CSCs. We therefore conclude that high PKCλ expression is required for ALDH1-postive cancer stem cell function and indicates a poor clinical outcome in late-stage breast cancer patients.

## Materials and methods

### Analysis of the METABRIC dataset

The Molecular Taxonomy of Breast Cancer International Consortium (METABRIC) dataset [53, 54] was downloaded from the cBioportal (https://www.cbioportal.org/) [55, 56] in March, 2019, after which the downloaded data were analyzed as we described previously [31, 32, 57]. The clinicopathological data from patients were shown in an earlier report [32]. The median age at the time of breast cancer diagnosis was 61.1 years in this dataset (range: 21 to 90 years). The numbers of patients with the indicated PAM 50 subtypes were as follows: normal-like, 148; luminal A, 679; luminal B, 461; HER2-enriched, 220; claudin-low, 199; and basal-like, 199. The gene alteration data were obtained from cBioportal, and the mRNA expression levels were compared using the Kruskal-Wallis test with the Steel-Dwass test. To construct survival curves, we defined the optimal cutoffs for the high- and low-expression groups using receiver operator characteristic (ROC) curves plotting expression of genes versus patient disease-specific survival rate (DSS) at several tumor stage. The optimal cutoff thresholds were determined using the Youden index. Survival curves were plotted using by the Kaplan-Meier method, and curves were compared using the log-rank (Cochran-Mantel-Haenszel) test. A multivariate Cox regression model was used to evaluate the influence of gene expression and to estimate adjusted hazard ratios (HRs) with age as a confounding factor. We also defined groups based on expression of *PKCλ*, *PKCζ*, and stemness markers: + (z-score > 0) and—(z-score < 0) in Table 1. The *p* values for the correlation between *PKCλ* or *PKCζ* and stemness marker expression were calculated using the χ^2^ test. For the heatmap of stemness gene and *PKCλ* and *PKCζ* expression (z-score) in Fig 2A, the average value of these genes was calculated and drawn as a heatmap using R version 3.5.2 (R Foundation for Statistical Computing Vienna, Austria). *p*-values below 0.05 were considered to be significant (* *p* < 0.05, ** *p* < 0.01, *** *p* < 0.001). All other statistical analyses were carried out using BellCurve for Excel ver. 3.00 (SSRI, JAPAN).

**Table 1:** Expression of *PKCλ an*d *PKCζ* and several stemness marker genes in basal-like breast cancer.

		*PKCλ*			*PKCζ*		
		(+)	(-)	*p*	(+)	(-)	*p*
*ALDH1A1*	(+)	24	20	0.017	16	28	0.063
	(-)	114	41		81	74	
*ALDH1A3*	(+)	87	49	0.016	62	74	0.191
	(-)	51	12		35	28	
*CD44*	(+)	68	25	0.280	42	51	0.344
	(-)	70	36		55	51	
*CD133*	(+)	129	52	0.062	84	97	0.037
	(-)	9	9		13	5	
*OCT4*	(+)	89	38	0.766	74	53	< 0.001
	(-)	49	23		23	49	
*SOX2*	(+)	30	13	0.946	23	20	0.482
	(-)	108	48		74	82	
*KLF4*	(+)	41	19	0.839	28	32	0.700
	(-)	97	42		69	70	
*c-MYC*	(+)	112	46	0.355	66	92	< 0.001
	(-)	26	15		31	10	
*NANOG*	(+)	75	30	0.501	51	54	0.959
	(-)	63	31		46	48	
*NOTCH1*	(+)	116	53	0.607	84	85	0.520
	(-)	22	8		13	17	
*NOTCH3*	(+)	92	43	0.594	61	74	0.145
	(-)	46	18		36	28	
*STAT3*	(+)	61	28	0.824	41	48	0.497
	(-)	77	33		56	54	
*BMI1*	(+)	28	9	0.355	16	21	0.458
	(-)	110	52		81	81	

The *p* values were calculated using the χ^2^ test.

### Analysis of the cancer genome atlas (TCGA) dataset

TCGA breast cancer dataset [58] was downloaded from Oncomine (https://www.oncomine.org; Compendia Bioscience, Ann Arbor, MI, USA) [59] in October, 2019 and from cBioportal (https://www.cbioportal.org) [55, 56] in March, 2019. The downloaded data were analyzed as we described previously [31, 32, 57]. Expression of PKCλ mRNA (reporter: A_23_P18392) and PKCζ mRNA (A_23_P51186) was compared between normal and cancerous tissues, both of which were available from TCGA breast cancer dataset, using the Wilcoxon signed rank test. Clinicopathological data from the breast cancer patients were presented in an earlier report [32]. The median age at the time of breast cancer diagnosis was 57.9 years in this dataset (range: 26 to 90 years). The dataset contains mRNA expression data from 61 normal breast tissue samples and 532 primary breast tumor samples. The gene alteration data were obtained from cBioportal. RNA expression was displayed using paired comparison of normal tissues vs. tumor tissues from all samples using R version 3.5.2 (R Foundation for Statistical Computing Vienna, Austria). The *p* values were calculated using the Wilcoxon signed-rank test. To make survival curves, we defined the optimal cutoffs for the high- and low-expression groups using ROC curves based on the expression of genes vs. patient OS in each cancer. Survival curves were plotted using the Kaplan-Meier method. The *p*-value for each cancer was calculated using the log-rank (Cochran-Mantel-Haenszel) test. A multivariate Cox regression model was used to evaluate the influence of gene expression and to estimate the adjusted HRs with age and sex as confounding factors. *p*-values below 0.05 were considered to be significant (* *p* < 0.05, ** *p* < 0.01, *** *p*< 0.001). All other statistical analyses were carried out using BellCurve for Excel ver. 3.00 (SSRI, JAPAN).

### Cell culture

Two human basal-like breast cancer cell lines (MDA-MB 157 and MDA-MB 468) were grown in Dulbecco’s modified Eagle’s medium (DMEM) medium supplemented with 10% fetal bovine serum (FBS) (Biosera) and a human normal-like (non-transformed) mammary epithelial cell line (MCF10A) were grown in mammary epithelial cell growth medium (MEGM; Lonza). These cell lines were purchased from the American Type Culture Collection (ATCC). The cells were then cultured as described previously [31, 32].

### PKCλ gene knockout using CRISPR-Cas9

The CRISPR online design tools CRISPR direct (https://crispr.dbcls.jp/) and CHOPCHOP (https://chopchop.cbu.uib.no/) were used to design a crRNA sequence targeting Exon1 of PRKCI (PKCλ) gene (5’-/AltR1/GCCGCCGCCUGCGACCGUGUGUUUUAGAGCUAUGCU/AltR2/-3’). Alt-R™ CRISPR-Cas9 Negative Control crRNA #1 (IDT) was used as control. The probability of off-target was checked using COSMID (https://crispr.bme.gatech.edu). Alt-R^®^ CRISPR-Cas9 tracrRNA—ATTO™ 550 (IDT) (100 μM) and Alt-R^®^ CRISPR-Cas9 crRNA (IDT) (100 μM) were mixed in Nuclease Free Duplex Buffer (IDT), heated at 95°C for 5 min, and cooled to room temperature to form 1 μM crRNA:tracrRNA duplex. The crRNA: tracrRNA duplex and diluted Alt-R® S.p. Cas9 Nuclease 3NLS (IDT) (1 μM) were mixed in Opti-MEM^®^ Media (Thermo) and incubated at room temperature for 5 min to form RNP complexes. The RNP complexes and Lipofectamine^®^ RNAiMAX Transfection Reagent (Invitrogen) were mixed in Opti-MEM^®^ Media and incubated at room temperature for 20 min to form transfection complexes, which were added to each well of a 24-well or 6-well tissue culture plate. Cells were then added to the transfection complexes at a concentration of 8–30 x 10^4^ cells/well. The final RNP concentration was 10 nM. The plate containing the transfection complexes and cells was incubated for 24 h or 48 h in a 5% CO_2_ incubator at 37ºC. Thereafter, high ATTO™ 550-intensity cells (top 0.2%) were sorted as single cells into a 96-well plate using a BD FACS Aria^TM^ II (BD Biosciences). The cells were then harvested, and PKCλ expression was assessed by immunoblotting.

### Immunoblotting

For growth in 2D cultures, ALDH1^high^ cells (approximately 5 x 10^4^ cells /well) were cultured in 12-well plates for 24 h in DMEM and then harvested using Trypsin. For tumor-sphere formation, ALDH1^high^ cells (2 x 10^4^ cells /well) were cultured for 3 days in DMEM supplemented with 10% FBS and 0.6% methylcellulose (see *in vitro* tumor-sphere culture section) and harvested manually. ALDH1^high^ cells were isolated using a cell sorter. The collected cells were washed three times with 1 x PBS and lysed in RIPA buffer (50 mM Tris (pH 8.0), 150 mM NaCl, 0.5 w/v% sodium deoxycholate, 0.1 w/v% SDS, 1.0 w/v% Nonidet p-40) plus 1 x protease inhibitor (Nacalai tesque) and 1 x phosphatase inhibitor (Nacalai tesque).

To detect ALDH1A3, Akt, pS473-Akt, pT308-Akt, p44/42 MAPK, p-p44/42 MAPK, PKCλ, and β-actin, aliquots of cell lysate containing approximately 20 μg of total proteins were subjected to SDS-PAGE (8% or 12% gel) and transferred (2 mA/cm^2^, 1 h) to Immobilon-P PVDF membranes (Milipore, IPVH00010). The membranes were then blocked with 5% skim milk in wash buffer (50 mM Tris (pH 7.5), 200 mM NaCl and 0.05% Tween20) or 5% BSA in TTBS (25 mM Tris (pH 7.5), 140 mM NaCl, 2.5mM KCI and 0.1% Tween 20) and incubated with the primary antibodies in dilution buffer containing 1% skim milk, 1% BSA or IMMUNOSHOT reagent 1 (CSR). The membranes were then probed with a horseradish peroxidase-conjugated secondary antibody in the same dilution buffer.

To detect Caspase-3 and Cleaved caspase-3, aliquots of lysate containing approximately 25 μg of total proteins were subjected to SDS-PAGE (15% gel) and transferred (2 mA/cm^2^, 30 min) to Immobilon-P PVDF membranes (Millipore, IPVH00010). The membranes were then blocked with 5% BSA in TTBS and incubated with the primary antibodies in IMMUNOSHOT reagent 1 (CSR), after which the membranes were probed with the horseradish peroxidase-conjugated secondary antibody in IMMUNOSHOT reagent 2 (CSR). Specific signals were detected with chemiluminescence reagent Immunostar LD (Wako) or EzWestLumiOne (ATTO) using Chemi Doc MP (Bio-Rad).

The antibodies used in this study were monoclonal mouse anti-PKCι(λ) (BD Biosciences, 610176, 1:2000), polyclonal rabbit anti-ALDH1A3 (Invitrogen, PA5-29188, 1:5000), polyclonal rabbit anti-Caspase-3 (CST, 9662S, 1:2000), polyclonal rabbit anti-cleaved caspase-3 (Asp175) (CST, 9661S, 1:1000), monoclonal rabbit anti-Akt1 (C73H10) (CST, 2938S, 1:2000), polyclonal rabbit anti-phospho Akt (Ser473) (736E11) (CST, 3787L, 1:2000), polyclonal rabbit anti-phospho Akt (Thr308) (244F9) (CST, 4056S, 1:2000), polyclonal rabbit anti-p44/42 MAPK (Erk1/2) (CST, 9102, 1:2000), polyclonal rabbit anti-Phospho-p44/42 MAPK (Erk1/2) (Thr202/Tyr204) (CST, 9101, 1:2000) and monoclonal mouse anti-β-actin (Proteintech, 60008-1-1g, 1:5000). The secondary antibodies used were horseradish peroxidase-conjugated anti-mouse IgG (CST, 7076, 1:3000) and anti-rabbit IgG (CST, 7074, 1:3000).

### Quantitative PCR

Total RNA was isolated using Sepasol®-RNA I Super G (Nacalai tesque) and Direct-zol^TM^ RNA miniprep (ZYMO) according to manufacturer’s instructions and reverse-transcribed using Rever Tra Ace qPCR RT Master Mix (TOYOBO). Gene expression assays were performed with THUNDERBIRD probe qPCR Mix (TOYOBO) according to the manufacturer’s instructions. The reaction protocol was as follows: 95°C for 1 min followed by 45 cycles of denaturation at 95°C for 10 s and extension at 60°C for 1 min. We carried out three independent experiments. 18S rRNA (ABI) was used to normalize differences in RNA input. Quantitative PCR primer and probe sequences are given in S1 Table.

### ALDEFLUOR assay

ALDH1^high^ cells were isolated from MDA-MB 157 and MDA-MB 468 cells and analyzed using an ALDEFLUOR assay kit (Stem Cell Technologies) as previously described [31, 32]. ALDH1^high^ cells were sorted using FACS Aria^TM^ II and III (BD Bioscience) and analyzed using a FACS Calibur (BD Bioscience). ALDH1^low^ cells were sorted as the 5% of the total population exhibiting the lowest ALDH1 activity. The data were analyzed using FlowJo 8.8.6 software (BD Bioscience).

### siRNA transfection

PKCλ knockdown in breast cancer cell lines was achieved by transfection of siRNAs (SIGMA) as previously described [32]. Briefly, siRNA targeting PKCλ was transfected using OPTI-MEM (Gibco) and Lipofectamine™ RNAiMAX Transfection Reagent (Invitrogen). The cells were seeded into 6-well plates at a concentration of 3.0 x 10^5^ cells/well and then treated with 10 nM (final concentration) siRNA transfection mixtures. The siRNAs used were MISSION siRNA Universal Negative Control (SIGMA) and PKCλ siRNA (5’-CAA GUG UUC UGA AGA GUU UTT-3’).

### *in vitro* tumor-sphere culture

Tumor-spheres were cultured as previously described [31, 32]. ALDH1^high^ cells isolated from the Negative control or PKCλ KO clone were plated in ultralow attachment 96-well plates (Greiner) (1 x 10^3^ cells/well) and cultured for 7 days in DMEM supplemented with 10% FBS and 0.6% methylcellulose. Images were captured with a DMIL LED (Leica). Sphere size was then measured as mean area of diameter (μm) for approximately 200 spheres using ImageJ Fiji software.

### Immunofluorescence

To stain for cleaved (active) caspase-3, ALDH1^high^ cells were isolated after knock down PKCλ for 48 h using targeted siRNA and then plated for 24 h in 8-well Lab-Tek chambers (Thermo) at a density of 5 x 10^3^ cells/well. For cell-pair assays to evaluate the asymmetric/symmetric distribution of ALDH1A3 and PKCλ, ALDH1^high^ cells were seeded onto micro cover glass (Matsunami) in a 24-well plate (Thermo) at density of 2 x 10^4^ cells/well. The cells were incubated for 24 h at 37°C in 5% CO_2_, then fixed in 2% paraformaldehyde (Wako) for 15 min, quenched in 100 mM Glycine (Kanto Chemical) for 10 min, and permeabilized with 0.1% Triton-X (SIGMA) for 10 min. Thereafter, they were blocked for 30 min in 10% fetal bovine serum (FBS; Biosera) and incubated overnight at 4°C with primary antibody diluted in 10% normal goat serum (NGS, Invitrogen). After three washes with TBST (20 mM Tris-HCl (pH 8.0), 150 mM NaCl, 0.05% Tween 20) for 5 min each, the samples were incubated with Alexa Fluor-conjugated secondary antibody for 1 h at room temperature and stained for 10 min with 0.1 μg/mL Hoechst 33342 (Invitrogen) diluted in PBS. After another three washes with TBST for 5 min each, the cells were mounted with Fluoro-KEEPER antifade reagent (Nacalai tesque) and ProLong Gold antifade reagent (Thermo). The primary antibodies used in this study were monoclonal mouse anti- PKCλ (BD Biosciences, 610207, 1:250), which recognizes PKCλ and PKCζ, polyclonal rabbit anti-ALDH1A3 (Invitrogen, PA5-29188, 1:500) and polyclonal rabbit anti-cleaved caspase-3 (D175) (CST, 9661S, 1:500). The secondary antibodies were anti-mouse Alexa 488 (CST, 4408S, 1:500) and anti-rabbit Alexa 555 (Invitrogen, A21429, 1:500). Images were captured using 6000B (Leica) and BZ-9000 (KEYENCE) microscopes. For the asymmetric cell distribution assay, more than 150 cells were counted.

### Transwell migration assays

Transwell migration assays were performed using 8.0 μm Falcon^<^ cell culture inserts (CORNING) in a 24 well plate (SIGMA). ALDH1^high^ cells were seeded into the upper chamber at a density of 5 x 10^4^ cells/well in DMEM supplemented with 5% FBS. The lower chamber contained DMEM supplemented with 10% FBS. After incubation for 24 h at 37°C, the transwell inserts were removed from the plate, washed twice with 1xPBS, and fixed in 2% paraformaldehyde (Wako) for 2 min and methanol for 20 min. After fixation, the inserts were washed twice with 1xPBS, stained for 15 min with 0.5% crystal violet (SIGMA), and again washed twice with 1xPBS. Cells in the upper chamber were then removed using a cotton swab. Images of migrated cell on the underside of the filter were captured using a DMIL LED microscope (Leica), and the numbers of cells in different 5 fields of view were counted.

### Trypan blue assays

In Fig 3J and 3K, ALDH1^high^ cells isolated after PKCλ KD for 48 h with targeted siRNA were plated in 12-well plates (Thermo) at a density of 2 x 10^4^ cells/well and incubated for 24 h. apoptosis inhibitor (using the pan-caspase inhibitor z-VAD-FMK) was purchased from PEPTIDE Institute Inc. and dissolved in 100% DMSO, making a 200 mM stock solution. In Fig 4F, ALDH1^high^ control cells or MDA-MB 157 PKCλ KO cells were plated in 12-well plates (Thermo) at a density of 2 x 10^4^ cells/well and incubated for 48 h with 100 μM z-VAD-FMK or 0.5% DMSO as a control. After staining with 0.4 w/v% Trypan blue solution (Wako), the cells were counted manually.

### Analysis of intracellular ROS levels

Sorted ALDH1^high^ cells were plated on 8-well Lab-Teck chamber slides (Thermo) at a density of 5 x 10^4^ cells/well and cultured for 48 h. To measure cellular ROS levels, the cells were stained with H2-DCFDA (Invitrogen), which generates fluorescent signals when oxidized by ROS in the cells. Cells were incubated with prewarmed 10 μM H2-DCFDA (diluted in PBS) staining solution for 30 min at 37°C and stained for 10 min with 0.1 μg/mL Hoechst 33342 (Invitrogen) diluted in PBS. All subsequent steps were performed in the dark. Images were captured using a 6000B microscope (Leica). Mean fluorescence values were determined using the ImageJ Fiji software.

## Results

### High expression of *PKCλ* mRNA in basal-like breast cancers exhibiting a low frequencies of gene amplification and mutation

**Fig 1:**
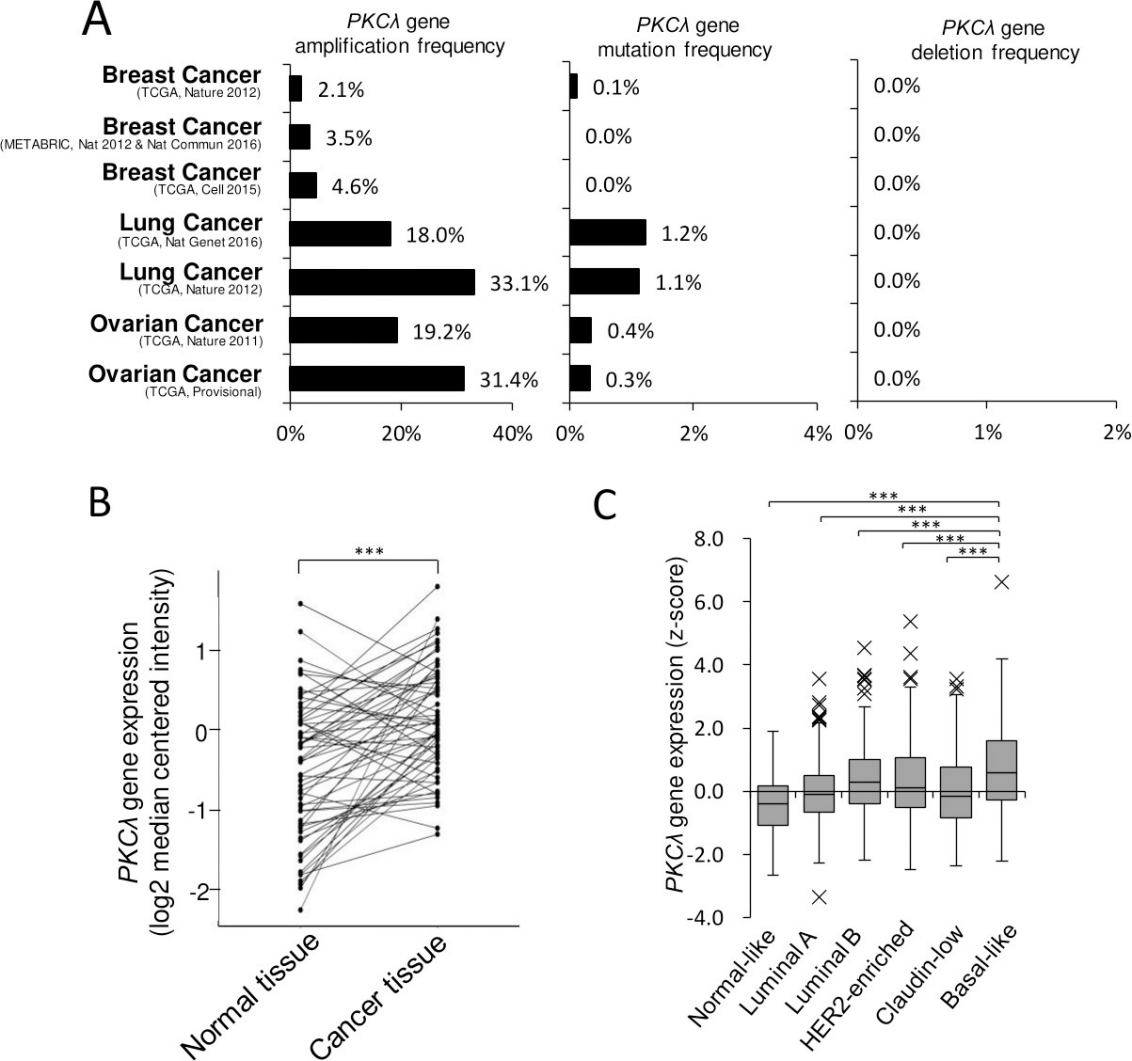
*PKCλ* mRNA overexpression with a low frequency of gene alterations in breast cancer. (A) *PKCλ* gene alterations (amplification, mutation and deep deletion frequency) in breast, lung, and ovarian cancers. (B) Paired comparison of PKCλ expression in normal and tumor tissues from TCGA dataset analysis of normal vs cancer tissue, n = 60. The *p* values were calculated using the Wilcoxon signed rank test. (C) *PKCλ* mRNA expression in breast cancer PAM50 subtypes from the METABRIC dataset. n = 1898. Centerline, median; box limits, lower (Q1) and upper (Q3) quartile, whiskers, ±1.5 x interquartile range (IQR); x, outlier. ***p < 0.001; Kruskal-Wallis test with Steel-Dwass test.

Earlier immunohistochemical analyses showed that PKCλ protein is overexpressed in a variety of human cancers, including breast cancer [37–48], and that amplification of its gene occurs in lung and ovarian cancers [39, 44]. To assess *PKCλ* gene alterations in breast cancer, we used two datasets: TCGA dataset from oncomine, which includes data from normal tissues, and the METABRIC dataset from cBIoportal, which lacks data from normal tissues. We first compared *PKCλ* gene alterations in breast cancers to those in lung and ovarian cancers. As shown in Fig 1A, the frequency of *PKCλ* gene amplification was lower in both breast cancer datasets tested (2.1% or 4.6% in TCGA, 3.5% in METABRIC) than in the lung (18.0% or 33.1% in TCGA) or ovarian (19.2% or 31.4% in TCGA) cancer datasets. In addition, there are few genetic mutations (0.1% in TCGA) and no deletions (0% in TCGA and METABRIC) in the breast cancer datasets. We therefore compared *PKCλ* mRNA expression between breast cancer and normal tissues derived from the same patients using TCGA dataset, which revealed that *PKCλ* expression was significantly higher in the cancers than normal tissues (Fig 1B). It appears, therefore, that higher *PKCλ* expression in breast cancers reflects increased transcription rather than gene amplification or mutation.

We next used the METABRIC dataset to examine in more detail the relationship between *PKCλ* overexpression and breast cancer PAM50 subtypes. As shown in Fig 1C, *PKCλ* expression was highest in basal-like breast cancers. This is consistent with the idea that overexpression of PKCλ protein contributes to tumorigenesis in TNBC, which is similar to the basal-like subtype [37].

### Correlation between *PKCλ* and *ALDH1A3* in basal-like breast cancer

**Fig 2:**
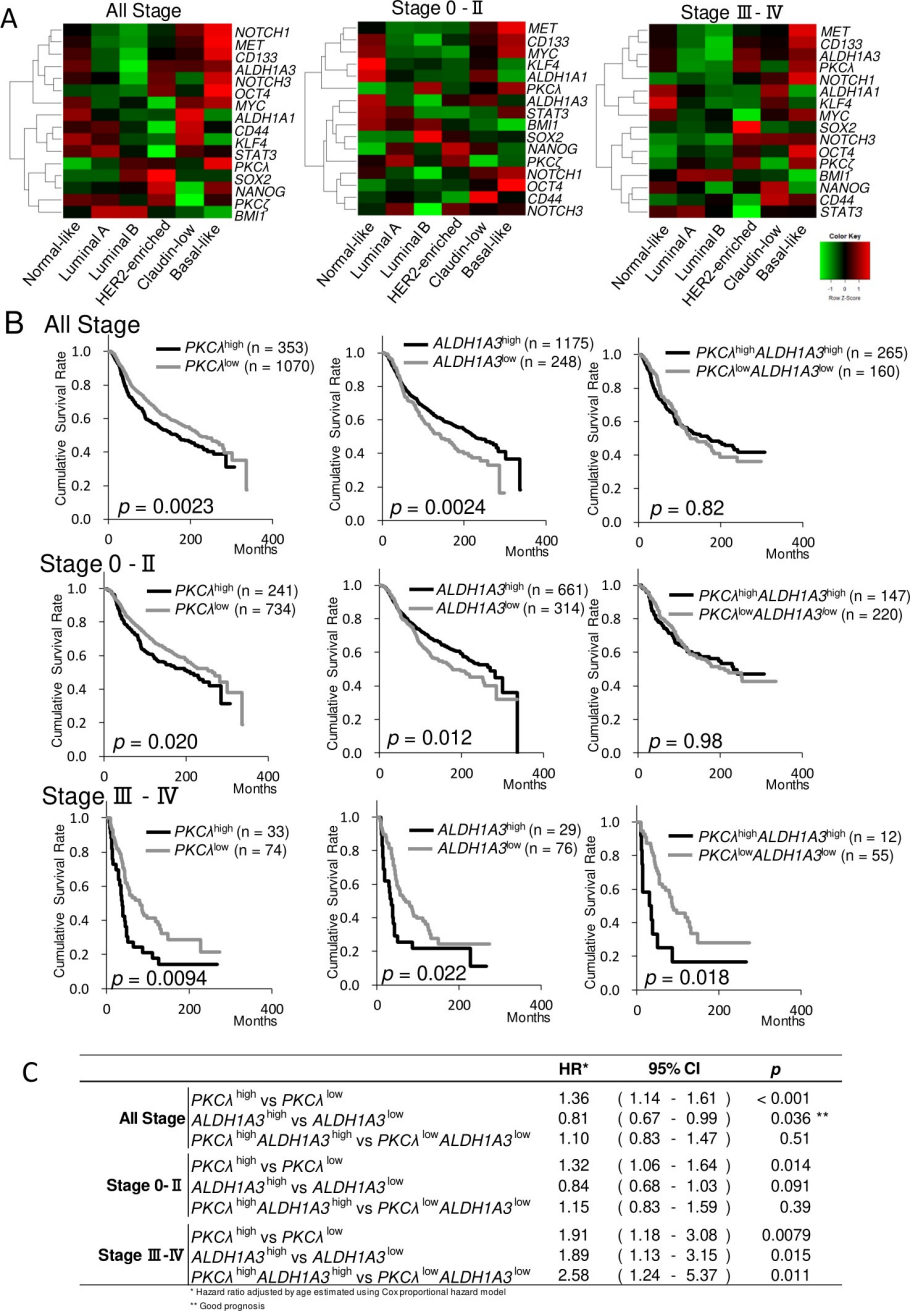
High expression of *PKCλ* and *ALDH1A3* contributes to poor clinical outcomes in breast cancer patients. (A) Heatmaps of the average levels of *PKCλ*, *PKCζ*, and stemness gene expression (z-score) in breast cancer PAM50 subtypes in the METABRIC dataset at several tumor stages. Raw z-scores were recalculated based on the average values. In the heatmap, red and green represent upregulated and downregulated genes, respectively: left panel, all stages; center panel, stages 0-II, right panel, stages III-IV. (B) Kaplan-Meier survival analysis of DSS in stage III-IV breast cancers from the METABARIC dataset. The *p* values were calculated using the log-rank test. (C) HRs for *PKCλ* and *ALDH1A3* expression estimated using a multivariate Cox regression model for DSS among breast cancers at several tumor stages.

Basal-like breast cancer is an aggressive subtype exhibiting stem-like properties [7, 8, 31, 32]. We therefore examined expression of *PKCλ* and several stem cell marker genes in several breast cancer subtypes. We found that *PKCλ*, along with *NOTCH1*, *MET*, *CD133*, *ALDH1A3*, *NOTCH3*, *OCT4*, *MYC* and *NANOG*, was enriched in basal-like breast cancer (Fig 2A, left panel; S1 Fig). Recently, Tokinaga-Uchiyama et al. reported that high expression of PKCλ protein associates with poor clinical outcomes in cases of stage III-IV cervical cancer [46]. In addition, we observed that *PKCλ* expression was highest in patients with stage III-IV basal-like tumors (S1 Fig). This prompted us to assess the relationship between expression of *PKCλ* and stem cell marker genes in stage III-IV breast cancer subtypes. We found that *PKCλ* was overexpressed in basal-like and HER2-enriched types, as was *ALDH1A3*, *CD133* and *OCT4* (Fig 2A, right panel). Moreover, *PKCλ* correlated with *ALDH1A1* and *ALDH1A3* in basal-like breast cancer (*ALDH1A1; p* = 0.017; *ALDH1A3; p* = 0.016, χ^2^-test). Among patients with basal-like cancers, the population co-expressing both *PKCλ* and *ALDH1A3* (43.7%; n = 87/199) was higher than that co-expressing *PKCλ* and *ALDH1A1* (12.1%; n = 24/199) (Table 1).

### Late-stage breast cancer patients exhibiting correlated expression of *PKCλ* and *ALDH1A3* had poorer clinical outcomes

To evaluate the prognosis of patients showing higher expression of *PKCλ* and/or *ALDH1A3*, we performed a Kaplan-Meier analysis of DSS. At all disease stages, patients expressing only *PKCλ*^high^ had a poorer prognosis (all stage, *p* = 0.0023; stage 0-II, *p =* 0.020; stage III-IV, *p =* 0.0094, log-rank test). By contrast, patients expressing *ALDH1A3*^high^ had a poorer prognosis only at stages III-IV (*p =* 0.022, log-rank test) (Fig 2B). Stage III-IV patients expressing both *PKCλ*^high^ and *ALDH1A3*^*high*^ also had poorer clinical outcomes (Fig 2B, *p* = 0.018, log-rank test). Multivariate analysis of DSS showed that patients expressing only *PKCλ*^high^ (HR 1.91, 95% CI 1.18–3.08, *p* = 0.0079) or both *PKCλ*^high^ and *ALDH1A3*^high^ (HR 2.58, 95% CI 1.24–5.37, *p* = 0.011) had poorer prognoses at stages III-IV (Fig 2C). Among breast cancer subtypes, tumors expressing *PKCλ*^high^ and *ALDH1A3*^high^ at late stages included a higher fraction of basal-like breast cancers (26.5%) than did all stages (20.6%) or early-stages (16.1%) (S2 Fig). These results suggest that *PKCλ* may be involved in cancerous progression and may contribute to poor clinical outcomes when expressed in ALDH1-positive CSCs in basal-like breast cancers at late tumor stages.

### PKCλ contributes to *in vitro* tumor-sphere formation by ALDH1^high^ cells

**Fig 3:**
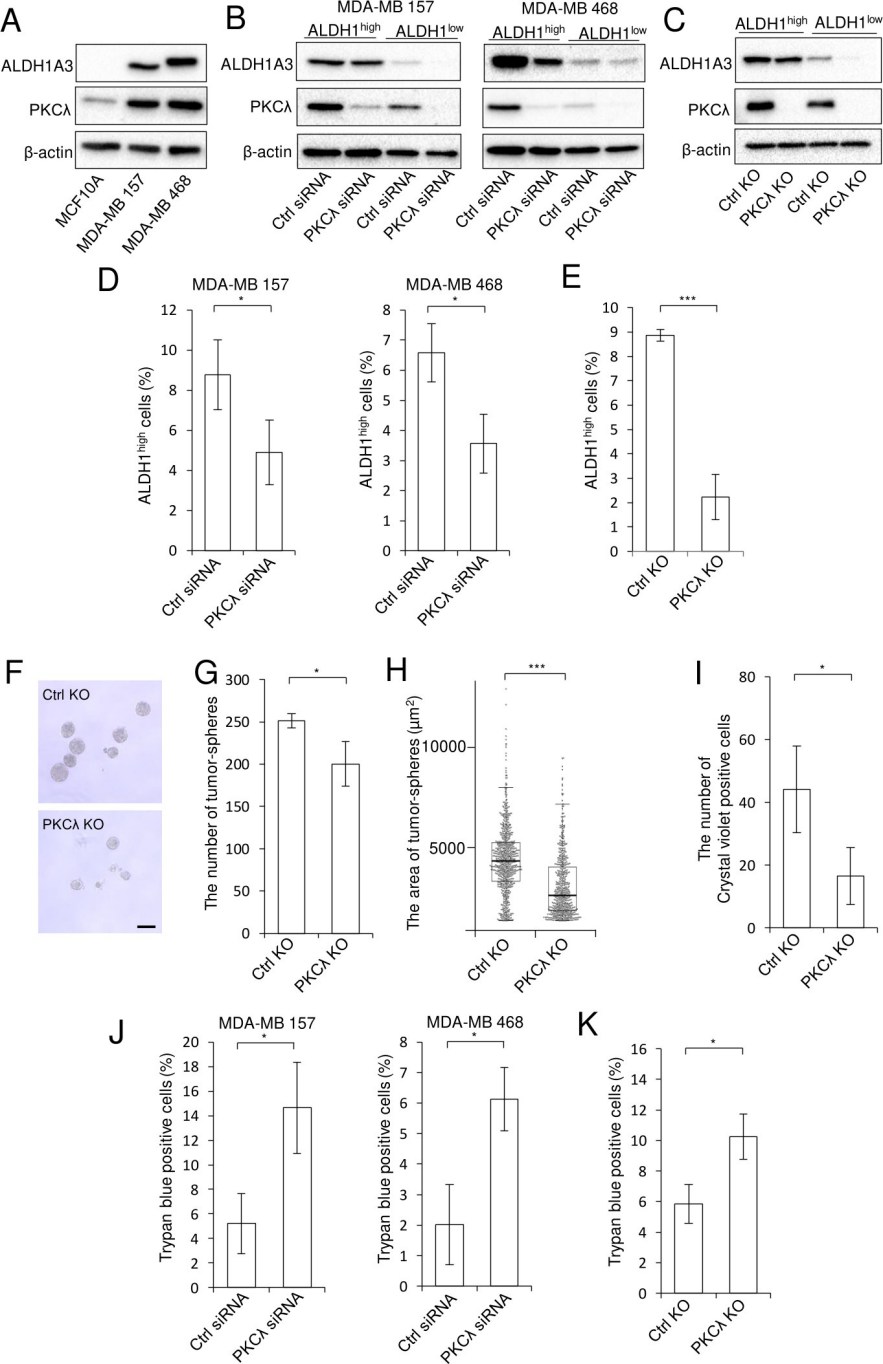
PKCλ depletion reduces *in vitro* tumor-sphere formation and migration and increases cell death among ALDH1^high^ cells. (A) Immunoblot analysis of PKCλ and ALDH1A3 expression in MCF10A, MDA-MB 157 and MDA-MB 468 cells. (B) Immunoblot analysis of PKCλ and ALDH1A3 expression in ALDH1^high^ and ALDH1^low^ cells isolated after PKCλ KD using targeted siRNA in MDA-MB 157 (left) and MDA-MB 468 (right) cells. (C) Immunoblot analysis of PKCλ and ALDH1A3 in ALDH1^high^ and ALDH1^low^ cells isolated from control or MDA-MB 157 PKCλ KO cells. In panels A-C, β-actin was used as an internal control. (D) ALDEFLUOR assays of the numbers of ALDH1^high^ cells isolated from among MDA-MB 157 (left) and MDA-MB 468 (right) cells after PKCλ KD using targeted siRNA. *p < 0.05, Student’s t-test. (E) ALDEFLUOR assays of the numbers of ALDH1^high^ cells isolated from among control or MDA-MB 157 PKCλ KO cells. *p < 0.05, ***p < 0.001, Student’s t-test. (F-H) *in vitro* tumor-sphere formation by ALDH1^high^ control or MDA-MB 157 PKCλ KO cells. Shown are representative images (F), numbers of tumor-spheres, *p < 0.05, Student’s t-test (G), and areas of the tumor-spheres, ***p < 0.001, two-sided Mann-Whitney’s U test (H). Scale bar, 50 μm. (I) Transwell migration of ALDH1^high^ control or MDA-MB 157 PKCλ KO cells. Cells that migrated to the lower side of the insert filter were stained with crystal violet and counted. *p < 0.05, Student’s t-test. (J) Trypan blue assays of ALDH1^high^ MDA-MB 157 (left) and MDA-MB 468 (right) cells performed 72 h after PKCλ siRNA transfection. (K) Trypan blue assays of ALDH1^high^ control or MDA-MB 157 PKCλ KO cells. * *p* < 0.05, Student’s t-test. Data depict the mean ± SD (three independent experiments).

Using the MDA-MB 157 and MDA-MB 468 human basal-like breast cancer cell lines, we observed that levels of PKCλ and ALDH1A3 expression were higher in both breast cancer cell lines than in human normal-like (non-transformed) MCF10A cells (Fig 3A). In addition, protein levels of both ALDH1A3 and PKCλ were higher in ALDH1^high^ than ALDH1^low^ cells isolated from among MDA-MB 157 and MDA-MB 468 cells (Fig 3B and [32]). The ALDH1^high^ cells also exhibited such CSC features as self-renewal, multidifferentiation and tumorigenesis to a greater degree than ALDH1^low^ cells [31]. Therefore, to further investigate the role of PKCλ in ALDH1-positive CSCs, we used two methods to inhibit the enzyme: siRNA-mediated knockdown (KD) and CRISPR-Cas9-mediated knockout (KO). As shown in Fig 3B and 3C, PKCλ KD or KO did not significantly affect levels of ALDH1A3 protein in ALDH1^high^ MDA-MB 157 cells, though PKCλ KD did elicit a decrease in ALDH1A3 levels in ALDH1^high^ MDA-MB 468 cells. In addition, ALDEFLUOR assays showed that the number of ALDH1^high^ cells was reduced in PKCλ-depleted cancer cells (Fig 3D and 3E). To assess the function of PKCλ in ALDH1-positive basal-like breast CSCs, we performed *in vitro* tumor-sphere assays using PKCλ-deficient ALDH1^high^ cells. PKCλ depletion in ALDH1^high^ cells led to decreases in both the number and size of tumor-spheres (Fig 3F–3H). These results suggest that PKCλ is involved in cell proliferation and/or survival and contributes to tumor-sphere formation by ALDH1-positive CSCs in basal-like breast cancer.

### PKCλ contributes to ALDH1^high^ cell migration

It is known that migration of the breast CSC population (CD44^+^ CD24^-/low^) gradually increases with tumor stage progression [13] and that PKCλ KD decreases the migration potential of multiple TNBC cell lines [37]. That suggests PKCλ plays an important role in the migration of ALDH1-positive CSCs. To further test that idea, we assessed the effect of PKCλ depletion on ALDH1^high^ cell migration. We found that PKCλ depletion in ALDH1^high^ cells led to a decrease in the number of migrated cells (Fig 3I), which suggests that PKCλ is required for ALDH1-positive basal-like breast CSC migration.

### PKCλ contributes to ALDH1^high^ cell survival

To determine the reason why PKCλ depletion led to decreases in the number of ALDH1^high^ cells and in tumor-sphere formation by ALDH1^high^ cells, we performed trypan blue assays with PKCλ-depleted ALDH1^high^ cells. We found that PKCλ depletion led to increases in the numbers of trypan blue-positive cells among ALDH1^high^ MDA-MB 157 and MDA-MB 468 cells (Fig 3J and 3K). We also examined the Akt and p44/42 MAPK phosphorylation status in PKCλ-depleted ALDH1^high^ cells. The levels of phospho-Akt (S473) were not significantly changed in PKCλ-deficient ALDH1^high^ cells. The levels of phospho-Akt (T308) were differed in between ALDH1^high^ PKCλ KD MDA-MB468 cells and PKCλ KO cells (S3 Fig). On the other hand, levels of phospho-44/42 MAPK were slightly enhanced in PKCλ-deficient ALDH1^high^ cells (S3 Fig). These results suggest that PKCλ is essential for the survival of ALDH1-positive basal-like breast CSCs with Akt and MAPK independent manner.

### PKCλ suppresses apoptosis of ALDH1^high^ cells

**Fig 4:**
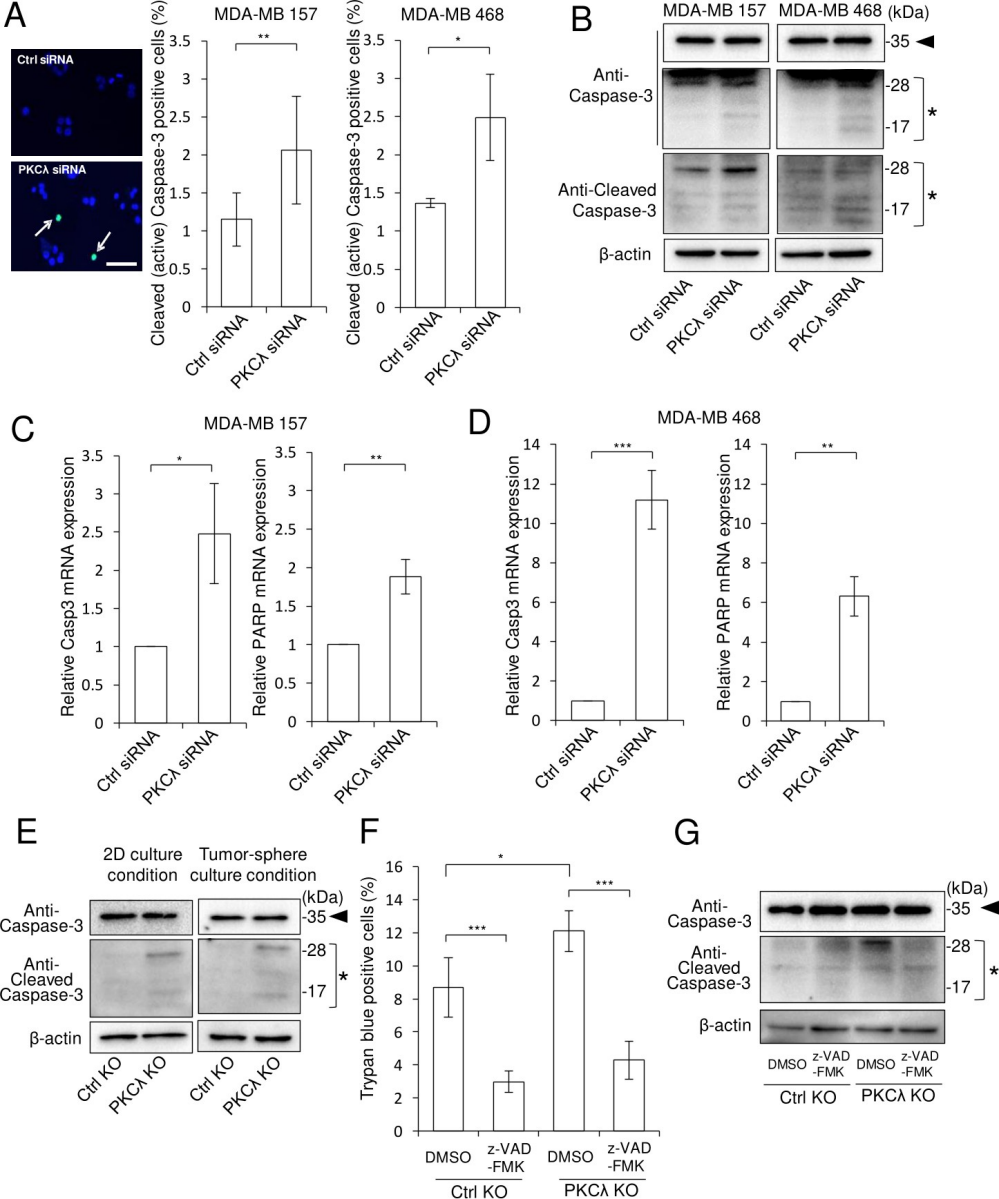
PKCλ depletion leads to apoptosis in ALDH1^high^ cells. (A) Numbers of cleaved (active) caspase-3-positive ALDH1^high^ MDA-MB 157 cells 72 h after PKCλ siRNA transfection (left, representative immunofluorescent staining of cleaved (active) caspase-3 (green) and Hoechst 33342 staining (blue); right, quantitative values). * *p* < 0.05, **p < 0.01 Student’s t-test. Scale bar; 100 μm. (B) Immunoblot analysis of intact and cleaved caspase-3 in ALDH1^high^ MDA-MB 157 (left) and MDA-MB 468 (right) cells 48 h after PKCλ siRNA transfection of ALDH1^high^ cells. β-actin was used as an internal control. (C-D) Quantitative PCR analysis of Casp3 and PARP in ALDH1^high^ cells isolated from MDA-MB 157 (C) and MDA-MB 468 (D) 48 h after PKCλ siRNA transfection. *p < 0.05, ** *p* < 0.01, *** *p* < 0.001, Student’s t-test. (E) Immunoblot analysis of intact and cleaved caspase-3 in ALDH1^high^ control or MDA-MB 157 PKCλ KO cells under 2D (left) and tumor-sphere culture condition (right). β-actin was used as an internal control. (F) Trypan blue assays in ALDH1^high^ control and MDA-MB 157 PKCλ KO cells after incubation with 0.5% DMSO (control) or 100 μM z-VAD-FMK for 48 h. * *p* < 0.05, *** *p* < 0.001, Tukey’s test. (G) Immunoblot analysis of intact and cleaved caspase-3 in ALDH1^high^ control or MDA-MB 157 PKCλ KO cells after incubation with 0.5% DMSO (control) or 100 μM z-VAD-FMK for 48 h. The arrowheads mark intact Caspase-3, and asterisks mark Cleaved Caspase-3. Data depict the mean ± SD (three independent experiments).

To determine more specifically how PKCλ depletion led to an increase in ALDH1^high^ cell death, we next considered whether PKCλ contributes to apoptosis in ALDH1^high^ cells. Fig 4A shows that PKCλ KD led to increased numbers of cleaved (active) caspase-3-positive ALDH1^high^ cells derived from both of MDA-MB 157 and MDA-MB 468 cells. PKCλ depletion also led to higher levels of cleaved caspase-3 protein (Fig 4B) as well as higher levels Casp3 and PARP mRNA in ALDH1^high^ cells (Fig 4C and 4D). In addition, PKCλ KO led to increases in the levels of cleaved casepase-3 protein within 2D culture condition and tumor-spheres formed by ALDH1^high^ cells (Fig 4E). Treating ALDH1^high^ cells with the apoptosis inhibitor (z-VAD-FMK) suppressed the cell death and reduced the levels of cleaved caspase-3 otherwise seen with PKCλ depletion (Fig 4F and 4G). These results suggest that PKCλ suppresses apoptosis and promotes cell survival among ALDH1-positive basal-like breast CSCs.

### PKCλ is required for asymmetric and symmetric ALDH1A3 protein distribution in paired cells

**Fig 5:**
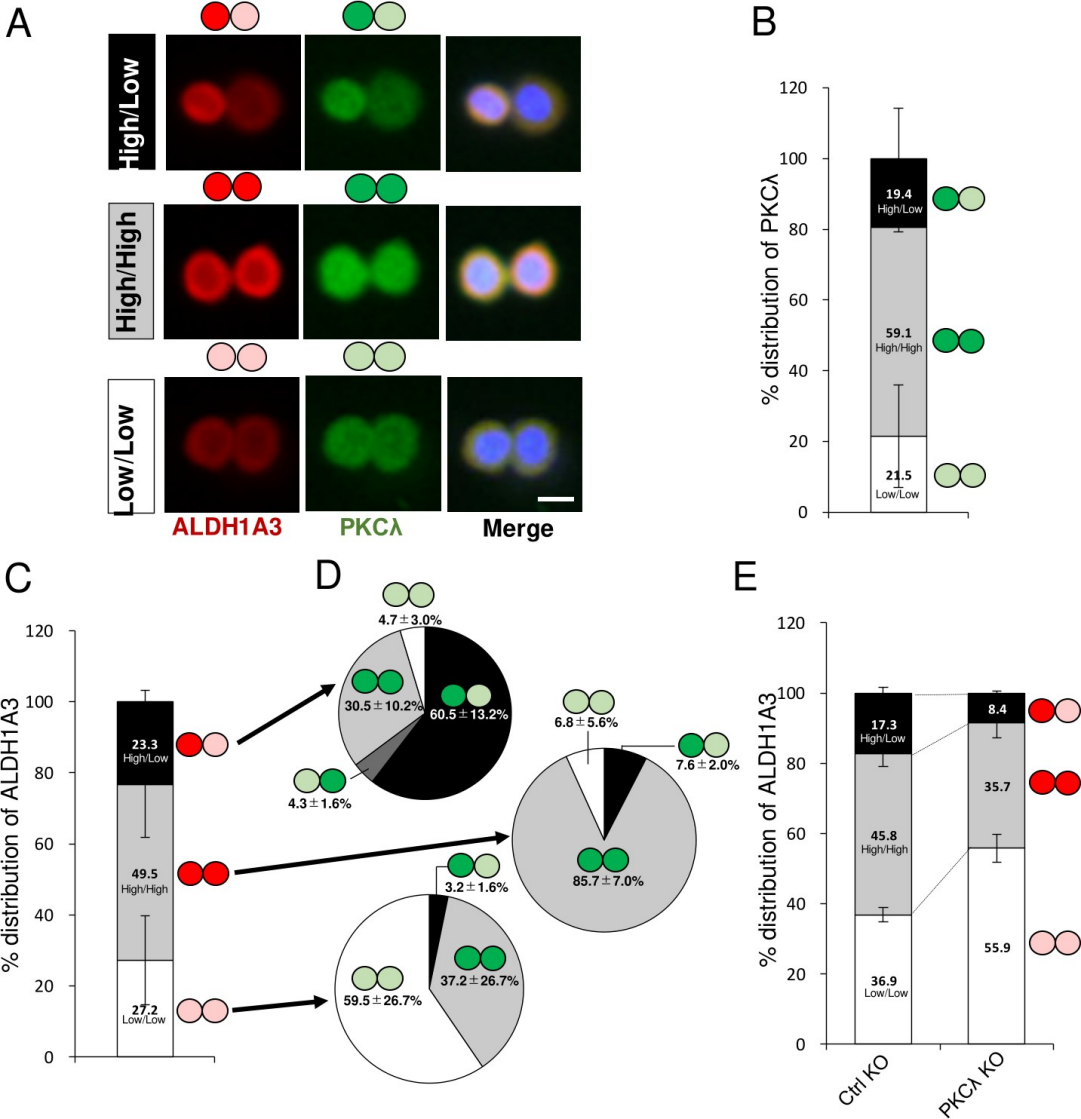
PKCλ depletion leads to changes in the asymmetric distribution of ALDH1A3 in ALDH1^high^ cells. (A) Representative images of three types of distributed cell pairs of ALDH1^high^ MDA-MB 157 cells. Cells were immunostained for ALDH1A3 (red) and PKCλ (green) and with Hoechst 33342 (Blue). (B) Quantitative analysis of the percentage of cell pairs exhibiting PKCλ High/Low, High/High, and Low/Low among MDA-MB 157 ALDH1^high^ cells after incubation for 24 h. (C) Quantitative analysis of percentages of MDA-MB 157 ALDH1^high^ cell pairs exhibiting of High/Low, High/High, and Low/Low ALDH1A3 distributions after incubation for 24 h. (D) Quantitative analysis of the percentages of MDA-MB 157 ALDH1^high^ cell pairs exhibiting High/Low, High/High and Low/Low PKCλ distributions within each of the three ALDH1A3 distributions after incubation for 24 h. (E) Quantitative analysis of the High/High, High/Low and Low/Low ALDH1A3 distributions exhibited by pairs of control MDA-MB 157 ALDH1^high^ cells and PKCλ KO cells after incubation for 24 h. Data depict the mean ± SD (three independent experiments). Scale bar, 10 μm.

An important and characteristic property of CSCs is their capacity for asymmetric cell division to propagate cancer stem-like cells or generate differentiated cells [15]. Because of the involvement of PKCλ in asymmetric cell division of both normal stem/progenitor cells [35, 36, 60] and CSCs [49], we hypothesized that PKCλ controls the balance between symmetric and asymmetric division of ALDH1-positive breast CSCs. To test that idea, we examined the asymmetric/symmetric distribution of PKCλ and ALDH1A3 proteins in ALDH1^high^ cells by immunofluorescently staining corresponding cell pairs for PKCλ and ALDH1A3. PKCλ was detected in both the asymmetric and symmetric fractions (High/Low, 19.4%; High/High, 59.1%; Low/Low, 21.5%) (Fig 5A and 5B). ALDH1A3 was also detected symmetric and asymmetric fractions (High/Low, 23.3%; High/High, 49.5%; Low/Low, 27.2%), and exhibited a similar expression distribution among cells (Fig 5A and 5C). Within cells, moreover, PKCλ largely colocalized with ALDH1A3 (Fig 5A). Within the ALDH1A3 asymmetric distribution (23.3% of total paired cells), higher PKCλ colocalized with higher ALDH1A3 levels, while lower PKCλ colocalized with lower ALDH1A3 levels (60.5%). Within the symmetric distribution (76.7% of total paired cells), higher ALDH1A3 colocalized with higher PKCλ in 85.7% of cells, while lower ALDH1A3 colocalized with lower PKCλ in 59.5% of cells. Higher PKCλ colocalized with lower ALDH1A3 in 37.2% of cells (Fig 5C and 5D). Interestingly, PKCλ depletion caused a decrease in the asymmetric distribution of ALDH1A3 (High/Low; 17.3% to 8.4%) and an increase in the symmetric distribution (High/High, 35.7% to 45.8%; Low/Low, 36.9% to 55.9%) (Fig 5E). These results suggest that PKCλ is required for both asymmetric and symmetric cell division of ALDH1-positive basal-like breast CSCs.

### PKCλ suppresses ROS accumulation in ALDH1^high^ cells

**Fig 6:**
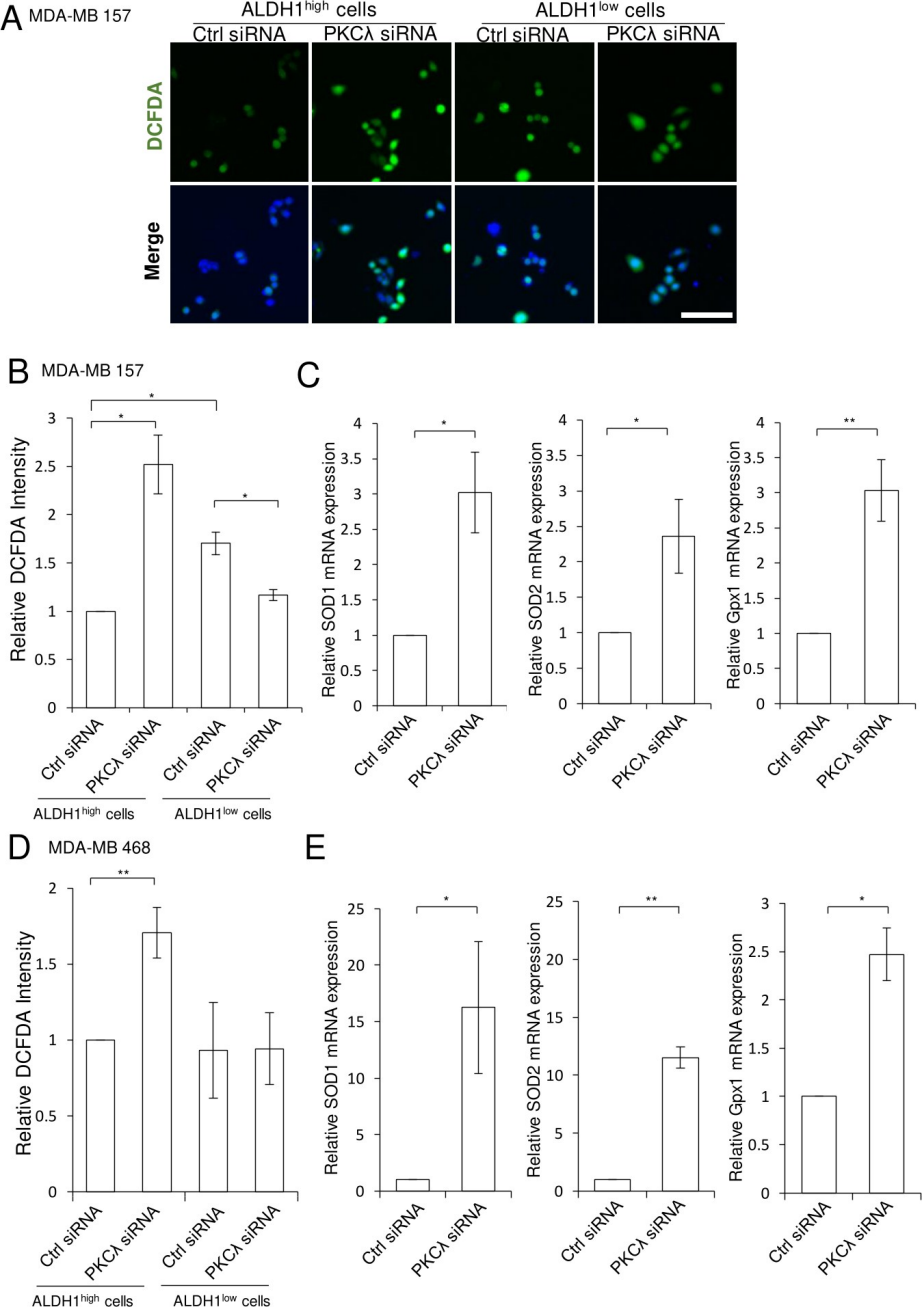
PKCλ depletion leads to increased intracellular ROS levels in ALDH1^high^ cells. (A) Representative images of DCFDA-positive cells in control KD and PKCλ KD of MDA-MB 157 ALDH1^high^ cells. Nuclei are stained with Hoechst 33342 (blue). (B) Intracellular ROS levels in ALDH1^high^ and ALDH1^low^ MDA-MB 157 after PKCλ siRNA transfection. *p < 0.05, Student’s t-test. Data depict the mean ± SD. (three independent experiments). (C) Quantitative PCR analysis of antioxidant genes (SOD1, SOD2 and Gpx1) 48 h after PKCλ siRNA transfection of ALDH1^high^ cells isolated from MDA-MB 157 cells. *p < 0.05, ** *p* < 0.01, Student’s t-test. Data depict the mean ± SE (three independent experiments). (D) Intracellular ROS levels in ALDH1^high^ and ALDH1^low^ MDA-MB 468 after PKCλ siRNA transfection. *p < 0.05, Student’s t-test. Data depict the mean ± SD. (four independent experiments). (E) Quantitative PCR analysis of antioxidant gene (SOD1, SOD2 and Gpx1) expression 48 h after PKCλ siRNA transfection of ALDH1^high^ cells isolated from MDA-MB 468 cells. *p < 0.05, ** *p* < 0.01, Student’s t-test. Data depict the mean ± SE (Three independent experiments). Scale bar; 100 μm.

ROS levels are reportedly lower in CSCs than non-CSCs in cancer [14]. A previous report suggested that increases in ROS can induce caspase-3-induced apoptosis [61]. We therefore assessed the contribution made by PKCλ to the maintenance of low ROS levels in ALDH1-positive CSCs. Fig 6 shows that MDA-MB157 ALDH1^high^ cells had lower ROS levels than ALDH1^low^ cells. In MDA-MB 468 cells, ROS levels did not differ between ALDH1^high^ and ALDH1^low^ cells. PKCλKD led to accumulation of ROS in ALDH1^high^ cells derived from MDA-MB 157 and MDA-MB 468, but led to reductions in ROS levels in ALDH1^low^ MDA-MB 157 cells but not MDA-MB 468 cells (Fig 6B and 6D). In addition, we proposed that depletion PKCλ may lead to enhanced expression of ROS defense genes, including SOD1, SOD2 and Gpx1, in response to ROS accumulation. Consistent with that idea, PKCλ depletion led to increased expression of SOD1, SOD2 and Gpx1 mRNA in response to ROS accumulation in ALDH1^high^ cells derived from both MDA-MB 157 and MDA-MB 468 cells (Fig 6C and 6E). These results suggest that PKCλ plays different roles in the regulation of ROS in CSCs and non-CSCs, and that PKCλ is at least involved in the maintenance of lower ROS-levels to protect ALDH1-positive breast CSCs from ROS damage.

## Discussion

Our findings show that breast cancer patients expressing both *PKCλ*^high^ and *ALDH1A3*^high^ exhibit poorer clinical outcomes at late-stage than those expressing *PKCλ*^low^ and *ALDH1A3*^low^ (Fig 2B and 2C). Moreover, among breast cancer subtypes, tumors showing *PKCλ*^high^ and *ALDH1A3* ^high^ at late stages include a higher frequency of basal-like breast cancers (26.5%) than all-stage (20.6%) or early-stage cancers (16.1%) (S2 Fig). Earlier studies indicate that it is ALDH1A3, not ALDH1A1, that contributes to ALDH activity in basal-like breast cancer cell lines [28–32], and ALDH1A3 is reported to positively associate with tumor grade, stage and metastasis [27]. Furthermore, Opdenaker et al. showed that there is a positive correlation between ALDH1A3 expression and tumor stage in TNBC patients [27], and we previously reported that *ALDH1A3* is highly expressed in basal-like breast cancers (Fig 3A and [32]). Given the correlation between *PKCλ* and *ALDH1A3* expression (Table 1), we suggest that PKCλ contributes to the cancerous progression of ALDH1-positive breast CSCs. Note that basal-like and late-stage breast cancers exhibit stemness [6–9], and that ALDH1 is involved in mediating chemoresistance through drug metabolism and detoxification of cellular aldehydes [62]. Consequently, a new pharmacological approach that targets PKCλ-dependent cellular regulation of ALDH1-positive CSCs is needed for the treatment of late-stage basal-like breast cancer.

One recent study suggests that PKCλ KD leads to a decrease in asymmetric cell division to generate CD133-positive and CD133-negative daughter cells in lung adenocarcinoma oncospheres [49]. In the present study, we revealed that PKCλ KD in MDA-MB 468 cells also leads to decreases in the numbers of CD133-postive cells (S4 Fig), suggesting PKCλ is involved in the stemness of CD133-postive breast CSCs. We further observed that CD133-poistive cells account for large fractions of both MDA-MB157 (46.1 ± 8.7%) and MDA-MB 468 cells (20.1 ± 6.7%) (S4 Fig). As a result, we deemed CD133 positivity not to be an effective indicator for distinguishing CSCs from non-CSCs MDA-MB 157 and MDA-MB 468 cells. This is consistent with an earlier report that in pancreatic cancer, CD133-positive cells are not significantly enriched among CSCs, but ALDH1^high^ cells are [22]. We therefore focused on examining the role of PKCλ in ALDH1-positive breast CSCs.

As shown in Fig 2A, *PKCλ*, *MET*, *NOTCH1*, *CD133*, *MYC*, *ALDH1A3*, *NOTCH3*, *OCT4*, and *CD44* were all enriched in stage III-IV basal-like breast cancers. Similarly, *PKCλ* and the stemness markers *ALDH1A3*, *CD133* and *OCT4* were highly expressed in HER2-enriched cancers (Fig 2A; S1 Fig). HER2 regulates the mammary stem/progenitor cell proliferation, driving tumorigenesis, invasiveness, and HER2-associated radioresistance in breast CSCs [63, 64]. This strongly suggests that the correlation between *PKCλ* and *ALDH1A3*, *CD133* and *OCT4* contributes to the cellular properties of HER2-positive breast CSCs. However, the functional roles of PKCλ in HER2-enriched breast CSCs remains to be determined.

Immunohistochemical studies indicate that patients exhibiting high PKCλ expression have poor prognoses in a variety of cancers [37–48]. And because ALDH1 is a CSC marker in a variety of cancers, we performed Kaplan-Meier and multivariable Cox regression analyses for *PKCλ* and *ALDH1A3* in several cancers (S5 Fig). The Kaplan-Meier analyses showed that patients expressing *PKCλ*^high^ and *ALDH1A3*^high^ had poorer prognoses in cases of pancreatic adenocarcinoma (*p* < 0.001, log-rank test), bladder urothelial carcinoma (*p* = 0.0052, log rank test) and ovarian serous cystadenocarcinoma (*p* < 0.01, log rank test). In a multivariable Cox regression analysis, patients with *PKCλ*^high^ and *ALDH1A3*^high^ had poorer prognoses in cases of pancreatic adenocarcinoma (HR 7.25, 95% CI 2.24–23.48, *p* < 0.001), bladder urothelial carcinoma (HR 2.0, 95% CI 1.29–3.10, *p* = 0.0020), colorectal adenocarcinoma (HR 3.42, 95% CI 1.10–10.63, *p* = 0.034) and ovarian serous cystadenocarcinoma (HR 1.53, 95% CI 1.13–2.08, *p* = 0.0066). On the other hand, patients with *PKCλ*^high^ and *ALDH1A3*^high^ had better prognoses in cases of acute myeloid leukemia (Kaplan-Meier analysis, *p* = 0.049, log rank test; multivariable Cox regression analysis, HR 0.53, 95% CI 0.31–0.90, p = 0.019) (S5 Fig). These results suggest that PKCλ is involved in regulating such cellular properties of ALDH1-positive CSCs as cell proliferation, survival, and migration; asymmetric cell division; and maintenance of lower ROS levels in diverse cancers in addition to breast cancer.

As with *PKCλ*, the aPKC subfamily member *PKCζ* exhibited low frequencies of gene amplification and mutation (S6A Fig), and expression of *PKCζ* was significantly higher in breast cancers than in normal tissues from the same patients (S6B Fig). Unlike *PKCλ*, however, *PKCζ* did not correlate with *ALDH1A3* in basal-like breast cancer (*p* = 0.191, χ^2^-test) (Table 1). We therefore limited our focus to the role of PKCλ in ALDH1-positive breast CSCs. Nonetheless, late-stage breast cancer patients expressing *PKCζ*^high^ and *ALDH1A3*^high^ had poorer clinical outcomes than patients expressing *PKCζ*^low^ and *ALDH1A3*^high^ (S6 Fig). The role of PKCζ in ALDH1-positive breast CSCs will need to be addressed in future studies.

An earlier report suggests that PKCλ activates NOTCH3 expression via ELF3 phosphorylation, and this axis maintains the highly tumorigenic TIC phenotype in KRAS-mediated lung adenocarcinoma [49]. Moreover, the PKCι(λ)-NOTCH1 pathway contributes to the survival of glioblastoma CSCs [50]. As shown in Fig 2A, both *NOTCH3* and *PKCλ* are highly expressed in basal-like cancers at all stages. Furthermore, patients expressing *PKCλ*^high^ and *NOTCH3*^high^ account for a large percentage of basal-like breast cancer patients (46.2%, n = 92 / 199) (Table 1). We therefore suggest that *PKCλ* plays a key role in determining the cellular properties of ALDH1-positive breast CSCs via NOTCH3 signaling. Deletion of PKCλ suppresses migration of MDA-MB 231, a TNBC cell line [37]. In Fig 3I, we show that PKCλ depletion suppresses migration of ALDH1^high^ cells. It may be that PKCλ is required for cell migration of ALDH1-positive breast CSCs. On the other hand, the results summarized in Figs 3 and 4 suggest that the suppression of ALDH1^high^ cell migration reflects increased cell death related to the PKCλ depletion.

It was previously suggested that activation of the PI3K / Akt pathway enriches the tumorigenic stem/progenitor cell population in breast cancer cell lines and tumor xenografts [65]. In addition, PI3K activates PKCλ [66]. In the present study, we found that the levels of phosphorylated Akt differed and the enhancement of phosphorylated MAPK in PKCλ-depleted ALDH1^high^ cells derived from breast cancer cell lines. This suggests that PKCλ is not essentially involved in the activation for Akt and MAPK in ALDH1-positive breast CSCs. That finding is consistent with the earlier report indicating that whether or not PKCλ-dependent activation of Akt and MAPK occurs depends on the cancer cell type [67]. Our results thus strongly suggest that PKCλ is essential for the survival of ALDH1-positive breast CSCs with independent manner of the activation of Akt and MAPK.

CSCs have a capacity for asymmetric cell division to propagate CSCs or generate differentiated cells [15]. This capacity for asymmetric cell division is thought to underlie the cellular heterogeneity of tumors. PKCλ is a master regulator of asymmetric cell division in multicellular organisms, including *C*. *elegance* and *Drosophila melanogaster* [35, 36]. PKCλ KD decreases asymmetric cell division to generate CD133-positive and CD133-negative daughter cells in lung adenocarcinoma oncospheres [49]. In present study, we observed that PKCλ KD alters the distribution of ALDH1A3 protein. Importantly, we detected 10 propagated patterns for ALDH1A3 and PKCλ (Fig 5C and 5D). This suggests PKCλ may be required for symmetric and asymmetric cell propagation of ALDH1A3-positive cells and may be a key contributor to the cellular heterogeneity seen in breast cancer.

Several recent studies indicate that CSCs contain less intracellular ROS than non-CSCs [14]. These lower ROS levels are reportedly associated with increased expression of free radical scavenging systems and contribute to radiotherapy resistance [14]. However, the mechanism by which the lower ROS levels are maintained is poorly understood. Our results suggest that PKCλ contributes to the maintenance of low ROS levels in ALDH1-positive breast CSCs (Fig 6). Inhibition of ALDH1 in breast cancer cells is associated with increased ROS levels [68, 69]. Furthermore, levels of ROS and ALDH1 activity are inversely related in melanoma [70]. It thus appears that ALDH1 activity mediates scavenging of ROS in CSCs. Human CD44^+^ CD24^-/low^ Lin^-^ and mouse Thy1^+^ CD24^+^ Lin^-^ CSC-enriched populations exhibit low ROS levels and higher expression of anti-ROS genes, including Foxo1, than a non-CSCs population [14]. Moreover, Foxo1 phosphorylation by PKCλ contributes to cell proliferation in angiosarcoma [71]. In addition, it has been reported that PKCζ, another PKC isoform, regulates glucose-6-phosphate dehydrogenase (G6PD) gene expression [72]. G6PD is the rate limiting enzyme in the pentose phosphate pathway (PPP). This is the major pathway for nicotinamide adenine dinucleotide phosphate (NADPH) generation [73], which is an essential cofactor for maintenance of redox balance within cells [74, 75]. Mele et al. reported that lapatinib, a tyrosine kinase inhibitor widely used for the treatment of breast cancer, and polydatin, a G6PD inhibitor, exerted a synergic effect on MCF7 (MCF7^mock^) cell viability, but had no effect on G6PD-overexpressing MCF7 (MCF7^G6PD+^) cells [76]. It was concluded that both PKCζ and PKCλ regulate G6PD gene expression and maintenance of NADPH levels in cells. We therefore suggest that one mechanism to maintain low ROS levels in CSCs may involve regulating PKCλ-dependent phosphorylation of Foxo1 or G6PD gene expression. On the other hand, PKCλ knockdown suppressed ROS levels in MDA-MB157 ALDH1^low^ cells, but did not change ROS levels in MDA-MB 468 ALDH1^low^ cells (Fig 6). Thus, the role of PKCλ in the regulation of ROS levels appears to differ between CSCs and non-CSCs.

ALDH1 is involved in the detoxification of toxic aldehyde intermediates, which are generated by ROS-induced peroxidation of intracellular lipids [77]. PKCλ depletion in ALDH1^high^ cells leads to increases in the numbers of apoptotic and dead cells (Fig 3J, 3K and Fig 4) and a corresponding increase in intracellular ROS (Fig 6). This suggests PKCλ may be involved in preventing apoptosis by maintaining lower intracellular ROS levels in ALDH1-positive breast CSCs. Because cancer cells are continuously exposed to ROS, they have developed protective mechanisms that enable proliferation, survival, migration and tumorigenesis, despite the presence of ROS in CSCs. The PKCλ-dependent maintenance of low ROS levels in ALDH1^high^ cells may be one of those mechanisms. However, the complex relationship between ROS levels and the cellular properties of CSCs remain unclear.

## Conclusions

In this study, we have shown that patients with stage III-IV breast cancer expressing *PKCλ*^high^ and *ALDH1A3*^high^ have poorer clinical outcomes than those expressing *PKCλ*^low^ and *ALDH1A3*^low^. Furthermore, PKCλ deficiency led to suppression of cell survival, tumor-sphere formation, migration, and asymmetric cell propagation, as well as intracellular accumulation of ROS in ALDH1-positive breast CSCs. PKCλ thus appears to be essential for the regulation for these cellular properties of ALDH1-positive breast CSCs. We therefore conclude that high PKCλ expression is required for ALDH1-postive cancer stem cell function and indicates a poor clinical outcome in late-stage breast cancer patients.

## Supporting information

S1 Fig *PKCλ*, *PKCζ*, *ALDH1A3*, *CD133*, and *OCT4* gene expression in breast cancer subtypes at tumor stages III-IV. mRNA expression in breast cancer PAM50 subtypes in stage III-IV patients from the METABRIC dataset: (A) *PKCλ*, (B) *PKCζ*, (C) *ALDH1A3*, (D) *CD133* and (E) *OCT4*. Centerline, median; box limits, lower (Q1) and upper (Q3) quartile; whiskers, ±1.5 x interquartile range (IQR); x, outlier. We could not find significant differences.
(TIF)

S2 Fig High rate of basal-like cancers among PAM50 subtypes in stage III-IV patients expressing *PKCλ*^high^ and *ALDH1A3* ^high^. Population rates (%) of PAM50 subtypes at several tumor stage in the METABRIC dataset.
(TIF)

S3 Fig Akt and MAPK phosphorylation status in PKCλ-depleted ALDH1^high^ cells. (A-B) Immunoblot analysis of Akt, phospho-Akt (S473), phospho-Akt (T308), p44/42 MAPK, phospho-p44/42 MAPK and PKCλ in ALDH1^high^ cells isolated after PKCλ KD using targeted siRNA in MDA-MB 157 (left) and MDA-MB 468 (right) cells (A) and in MDA-MB 157 PKCλ KO cells (B). β-actin was used as an internal control.
(TIF)

S4 Fig PKCλ KD leads to decreases in CD133-positive cells. Numbers of CD133-positive cells after 48h PKCλ KD (left, MDA-MB 157; right, MDA-MB 468). *p < 0.05, Student’s t-test. Data depict the mean ± SD (three independent experiments).
(TIF)

S5 Fig Patients co-expressing both *PKCλ* and *ALDH1A3* have poorer prognoses in various cancers. (A) Kaplan-Meier survival curves for OS in pancreatic adenocarcinoma, bladder urothelial carcinoma, acute myeloid leukemia, and ovarian serous cystadenocarcinoma from the TCGA dataset. The *p* values were calculated using the log-rank test. (B) For the indicated cancers, *p* values were calculated using log-rank test. (C) Multivariable Cox regression analysis of OS in several cancers.
(TIF)

S6 Fig *PKCζ* overexpression exhibits low gene alteration frequency and indicates poorer prognoses in tumor stage III-IV breast cancer patients. (A) *PKCζ* gene alteration (amplification, mutation or deep deletion frequency) in several cancer type datasets. (B) Comparison of *PKCζ* expression in normal tissue and tumor tissue from TCGA dataset (***p < 0.001, Wilcoxon signed rank test) (n = 60). (C) *PKCζ* mRNA expression in the indicated breast cancer PAM50 subtypes from the METABRIC dataset. Centerline, median; box limits, lower (Q1) and upper (Q3) quartile; whiskers, ± 1.5 x interquartile range (IQR); x, outlier. **p < 0.01; Kruskal-Wallis test with Steel-Dwass test. (D) Kaplan-Meier survival curves for DSS in stage III-IV breast cancer from the METABARIC dataset. The *p* value was calculated using the log-rank test. (E) Multivariable Cox regression analysis of DSS in breast cancer subtypes at several tumor stages.
(TIF)

S1 Table Quantitative PCR primer and probe sequences. (XLSX)

S1 Text Flow cytometry analysis. (DOCX)

S1 Raw images (PDF)
