# Supplementary figures and images for: High PKCλ expression is required for ALDH1-positive cancer stem cell function and indicates a poor clinical outcome in late-stage breast cancer patients
Source: PLoS One. 2020 Jul 13;15(7):e0235747. doi: 10.1371/journal.pone.0235747 (PMC7357771; doi:10.1371/journal.pone.0235747)

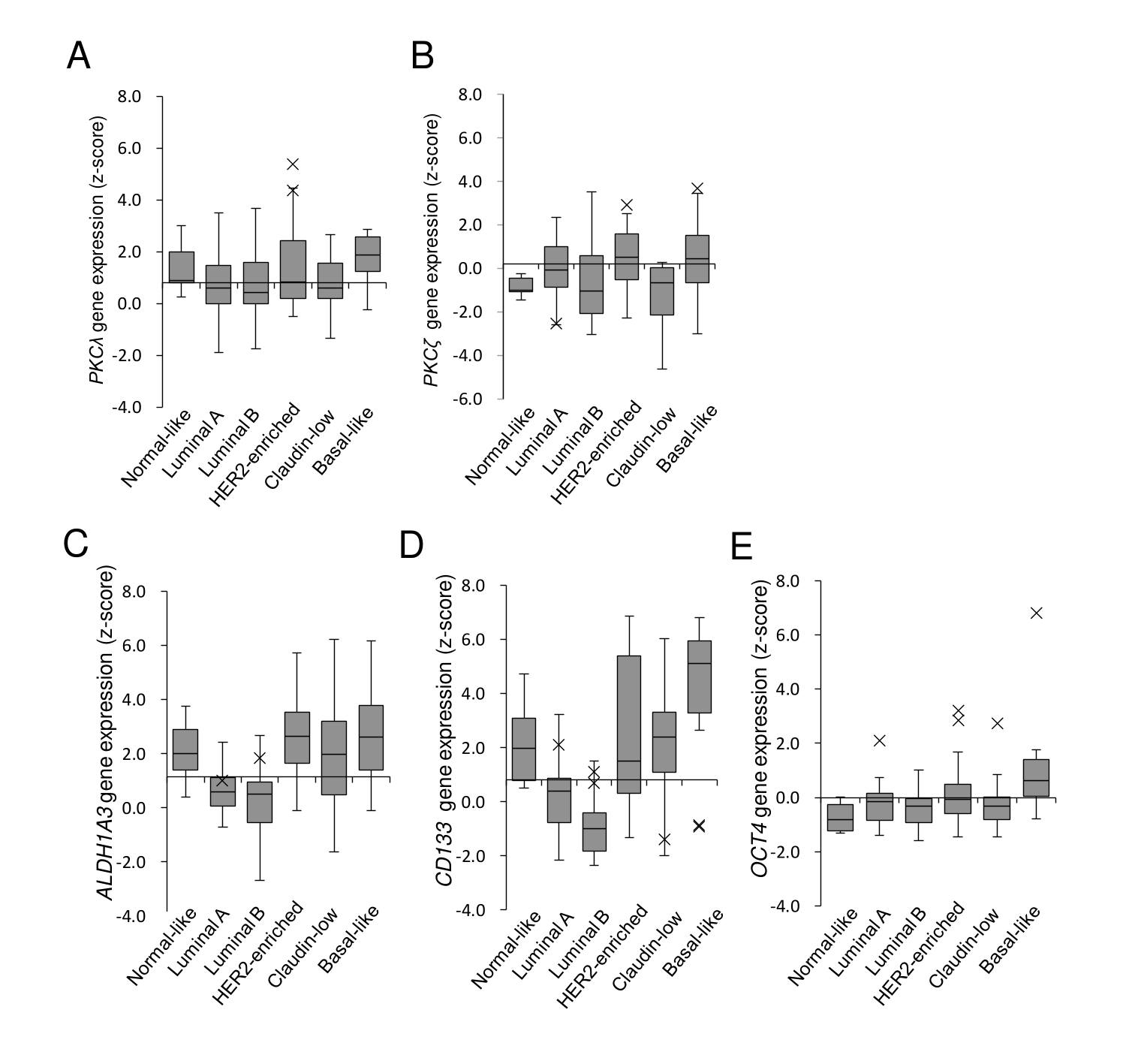

Supplement: S1 Fig — mRNA expression in breast cancer PAM50 subtypes in stage III-IV patients from the METABRIC dataset: (A) PKCλ, (B) PKCζ, (C) ALDH1A3, (D) CD133 and (E) OCT4. Centerline, median; box limits, lower (Q1) and upper (Q3) quartile; whiskers, ±1.5 x interquartile range (IQR); x, outlier. We could not find significant differences. (TIF) [file pone.0235747.s001.tif]

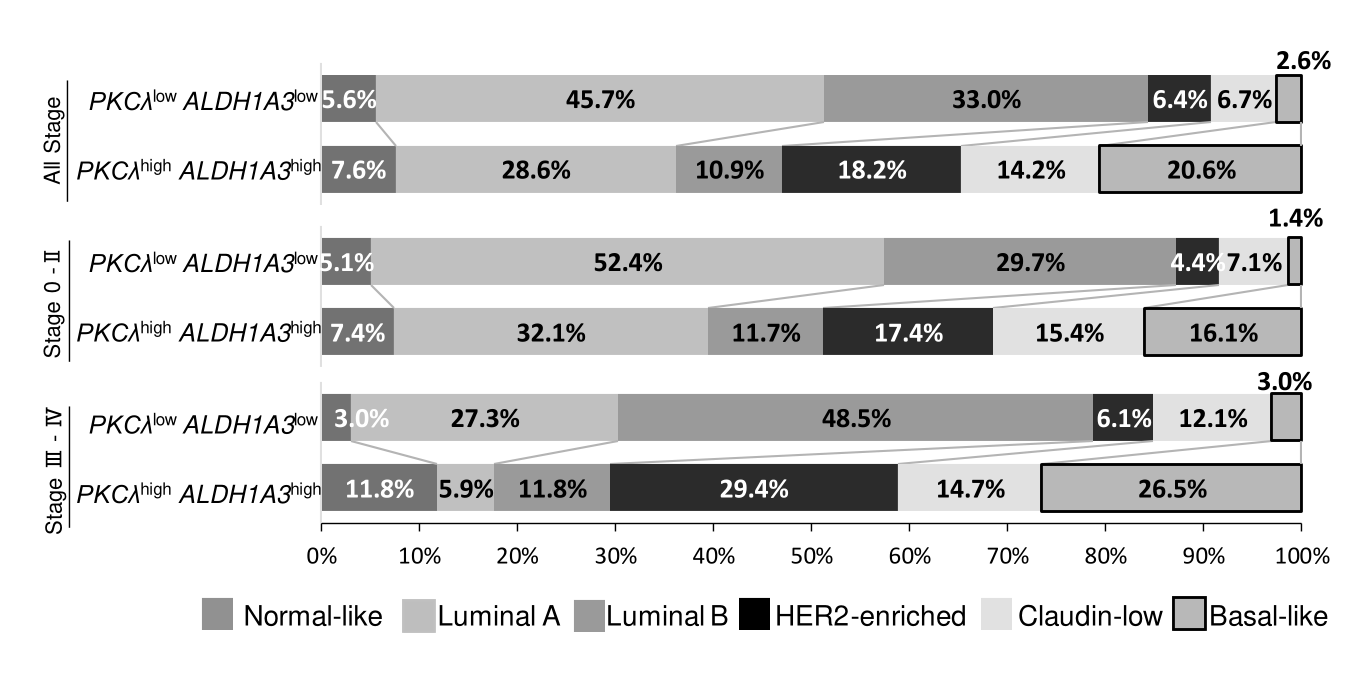

Supplement: S2 Fig — Population rates (%) of PAM50 subtypes at several tumor stage in the METABRIC dataset. (TIF) [file pone.0235747.s002.tif]

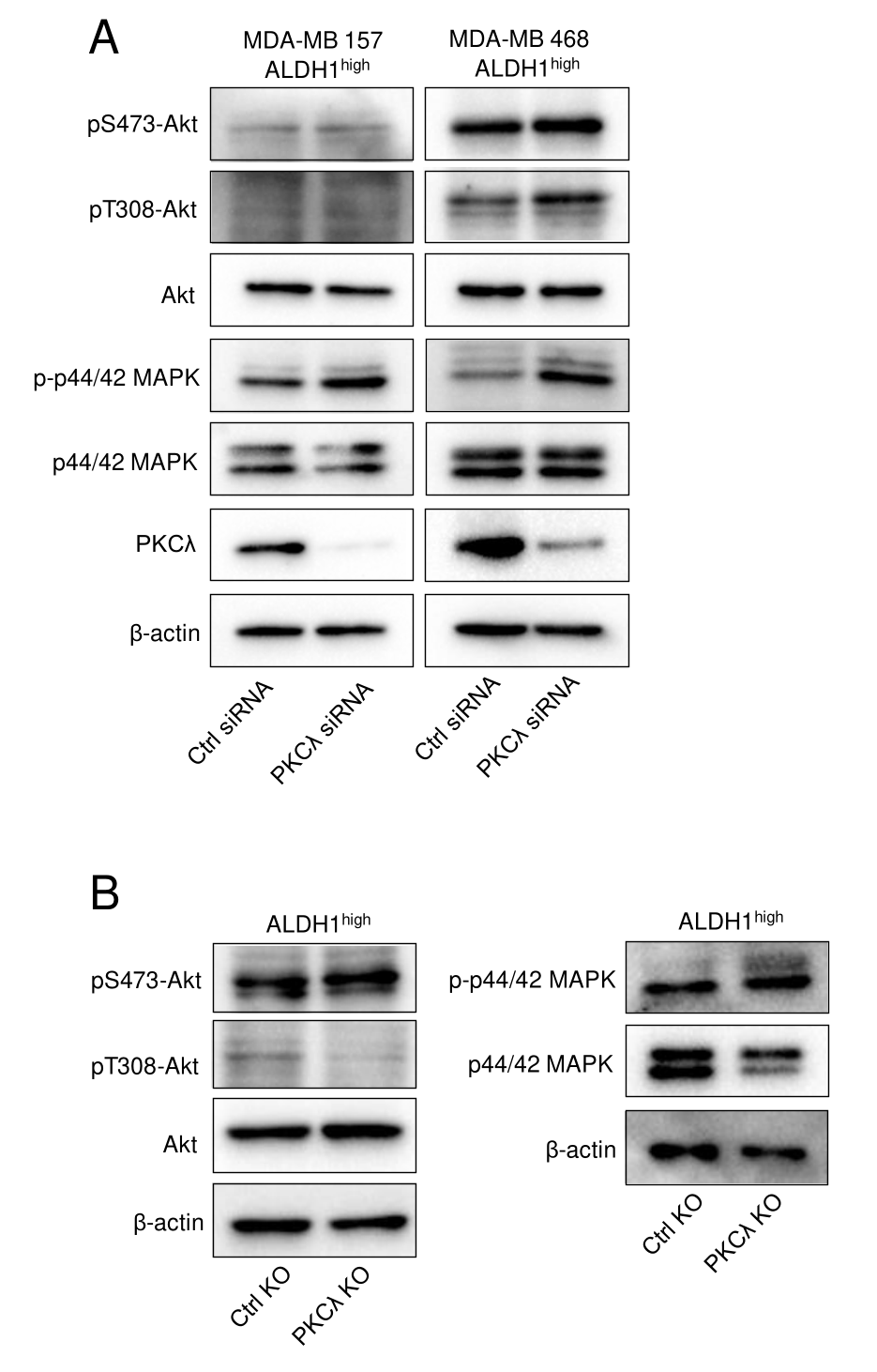

Supplement: S3 Fig — (A-B) Immunoblot analysis of Akt, phospho-Akt (S473), phospho-Akt (T308), p44/42 MAPK, phospho-p44/42 MAPK and PKCλ in ALDH1high cells isolated after PKCλ KD using targeted siRNA in MDA-MB 157 (left) and MDA-MB 468 (right) cells (A) and in MDA-MB 157 PKCλ KO cells (B). β-actin was used as an internal control. (TIF) [file pone.0235747.s003.tif]

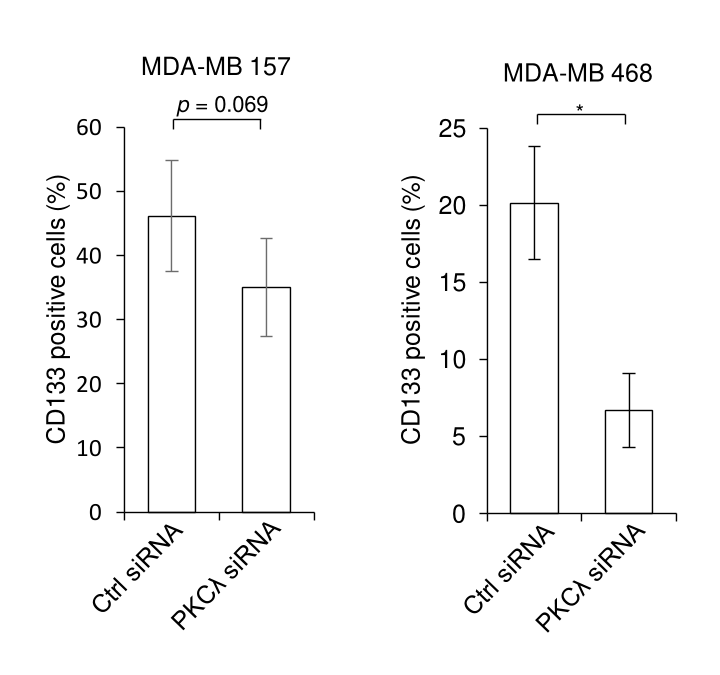

Supplement: S4 Fig — Numbers of CD133-positive cells after 48h PKCλ KD (left, MDA-MB 157; right, MDA-MB 468). *p < 0.05, Student’s t-test. Data depict the mean ± SD (three independent experiments). (TIF) [file pone.0235747.s004.tif]

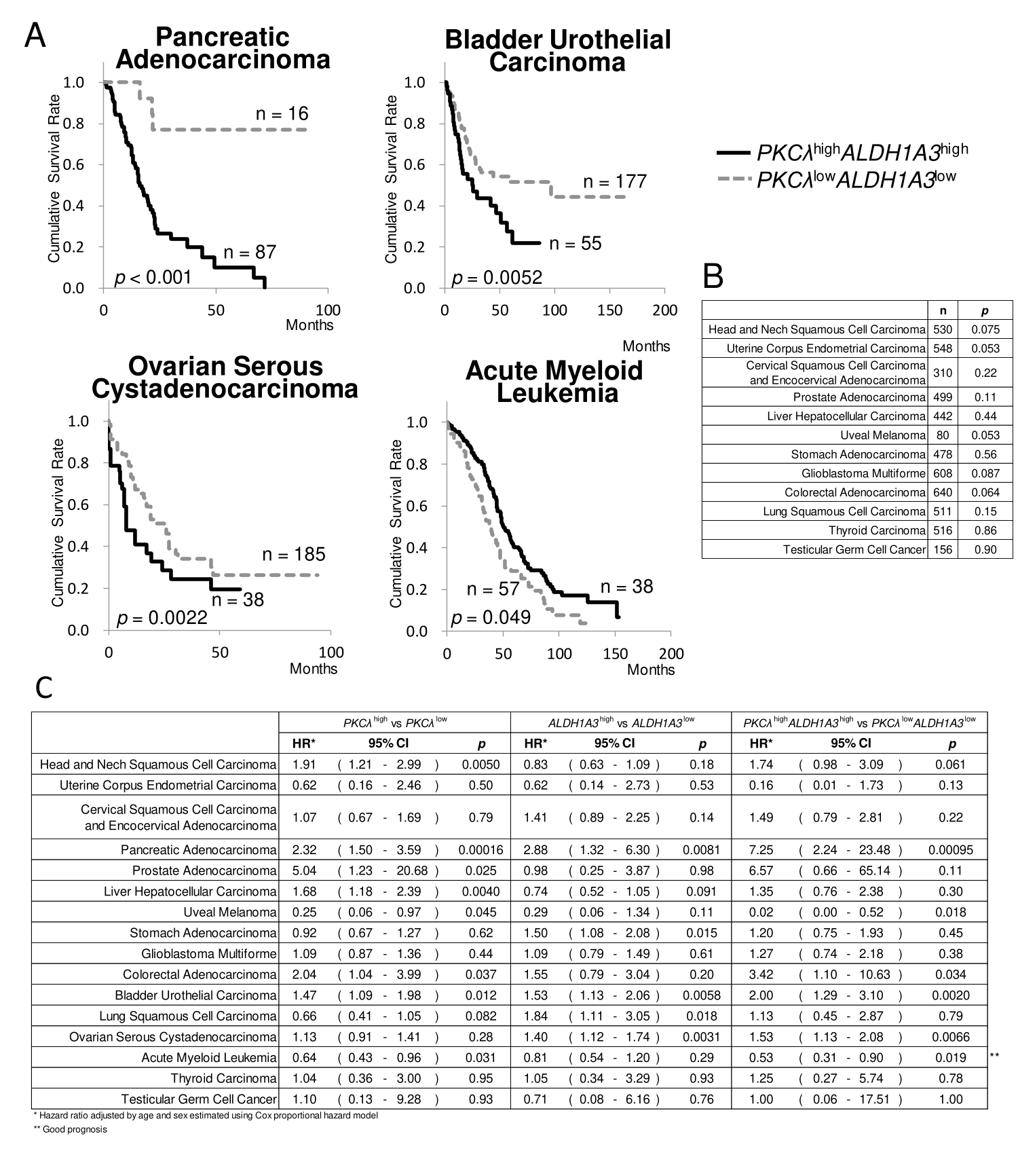

Supplement: S5 Fig — (A) Kaplan-Meier survival curves for OS in pancreatic adenocarcinoma, bladder urothelial carcinoma, acute myeloid leukemia, and ovarian serous cystadenocarcinoma from the TCGA dataset. The p values were calculated using the log-rank test. (B) For the indicated cancers, p values were calculated using log-rank test. (C) Multivariable Cox regression analysis of OS in several cancers. (TIF) [file pone.0235747.s005.tif]

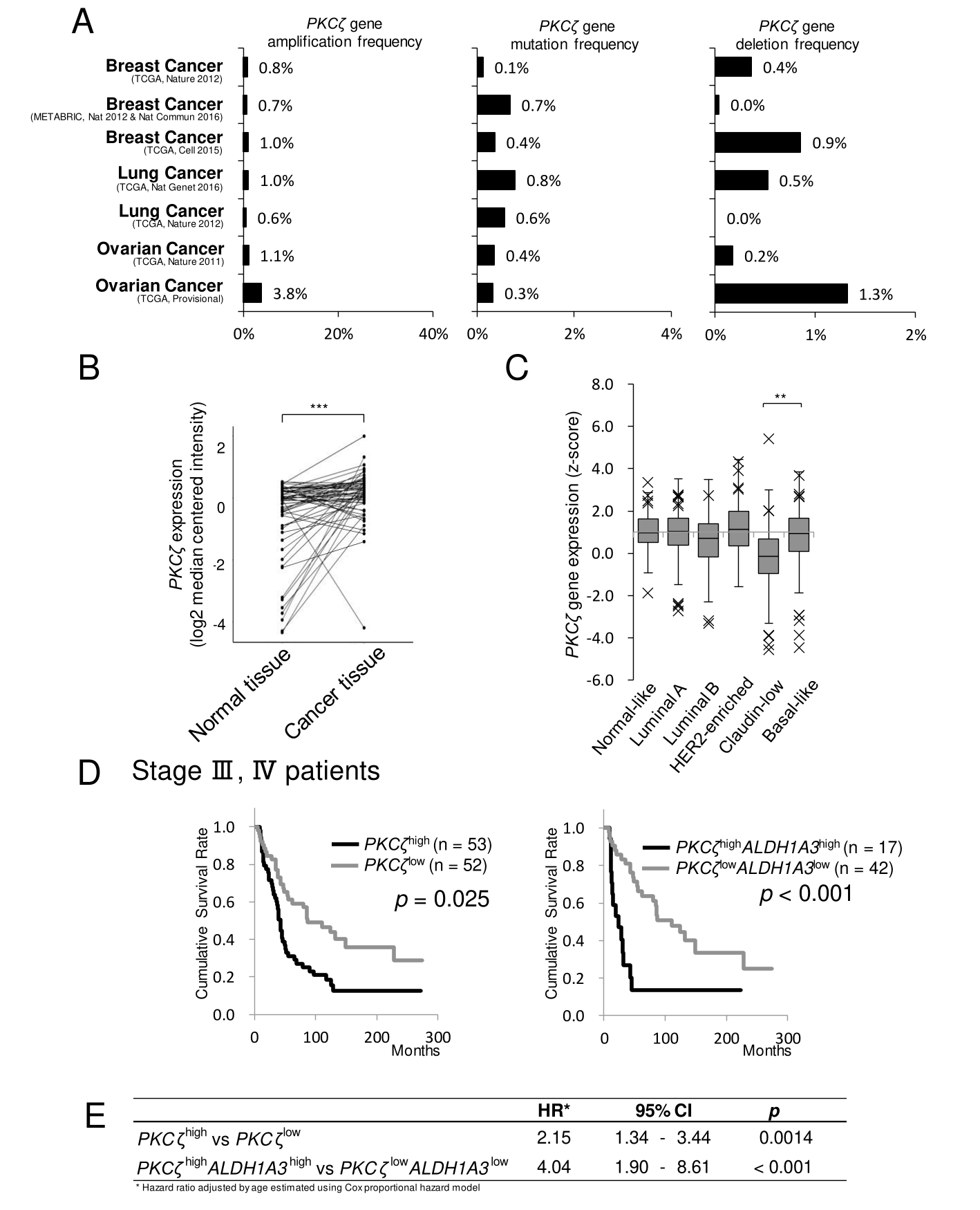

Supplement: S6 Fig — (A) PKCζ gene alteration (amplification, mutation or deep deletion frequency) in several cancer type datasets. (B) Comparison of PKCζ expression in normal tissue and tumor tissue from TCGA dataset (***p < 0.001, Wilcoxon signed rank test) (n = 60). (C) PKCζ mRNA expression in the indicated breast cancer PAM50 subtypes from the METABRIC dataset. Centerline, median; box limits, lower (Q1) and upper (Q3) quartile; whiskers, ± 1.5 x interquartile range (IQR); x, outlier. **p < 0.01; Kruskal-Wallis test with Steel-Dwass test. (D) Kaplan-Meier survival curves for DSS in stage III-IV breast cancer from the METABARIC dataset. The p value was calculated using the log-rank test. (E) Multivariable Cox regression analysis of DSS in breast cancer subtypes at several tumor stages. (TIF) [file pone.0235747.s006.tif]
