# Supplementary material for: High PKCλ expression is required for ALDH1-positive cancer stem cell function and indicates a poor clinical outcome in late-stage breast cancer patients
Source: PLoS One. 2020 Jul 13;15(7):e0235747. doi: 10.1371/journal.pone.0235747 (PMC7357771; doi:10.1371/journal.pone.0235747)
Supplement: S1 Text — (DOCX) [file pone.0235747.s008.docx]

**Flow cytometry analysis**

CD133-positive cells were isolated from among MDA-MB 157 and MDA-MB 468 cells and analyzed using a cell analyzer (Calibur, BD bioscience) and FlowJo 8.8.4 software as described previously [32]. The anti-CD133 APC antibody (293C3) (Miltenyi Biotec, 130-090-854) was purchased from BD Bioscience.
