# Supplementary material for: High PKCλ expression is required for ALDH1-positive cancer stem cell function and indicates a poor clinical outcome in late-stage breast cancer patients
Source: PLoS One. 2020 Jul 13;15(7):e0235747. doi: 10.1371/journal.pone.0235747 (PMC7357771; doi:10.1371/journal.pone.0235747)

Figure 3A ALDH1A3

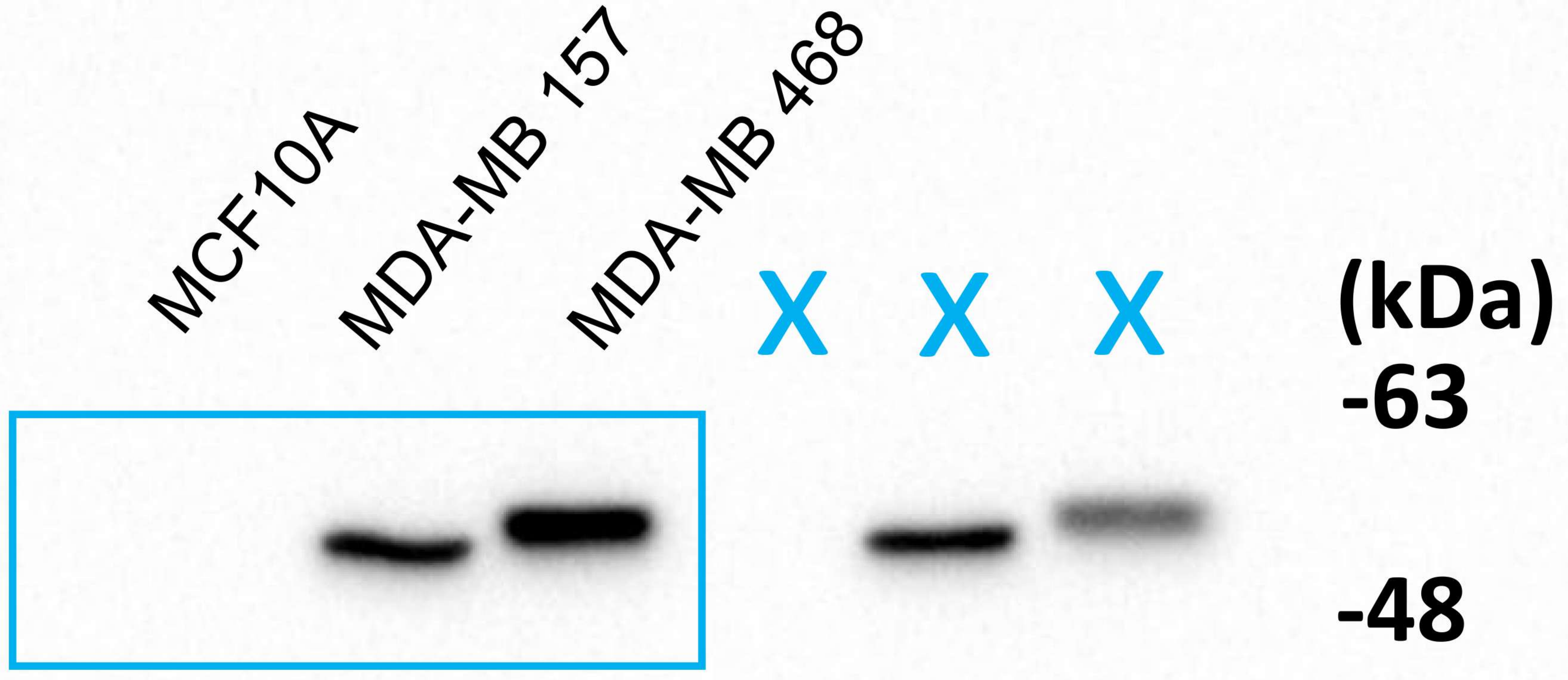

Figure 3A PKCλ

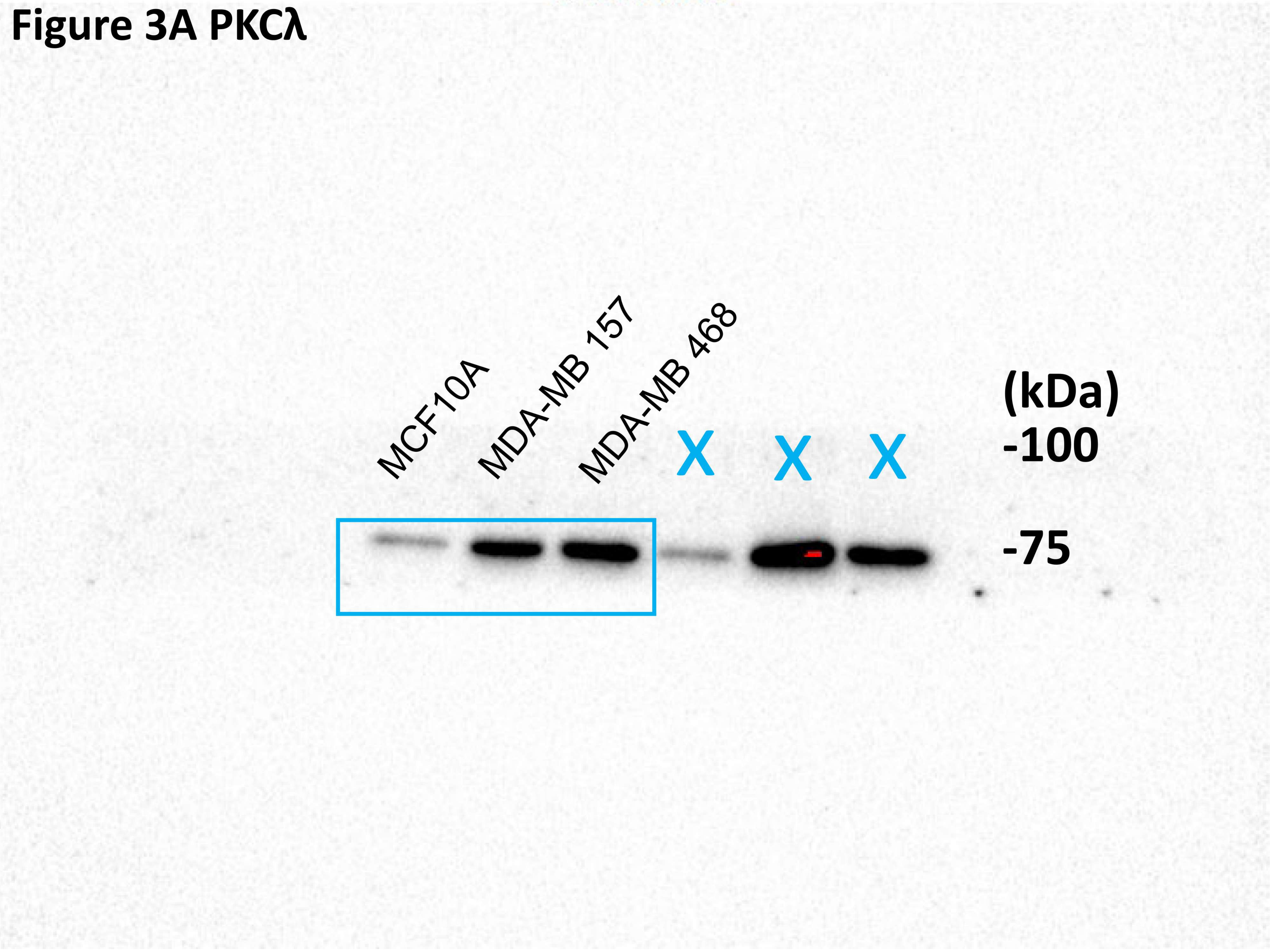

Figure 3A β-actin

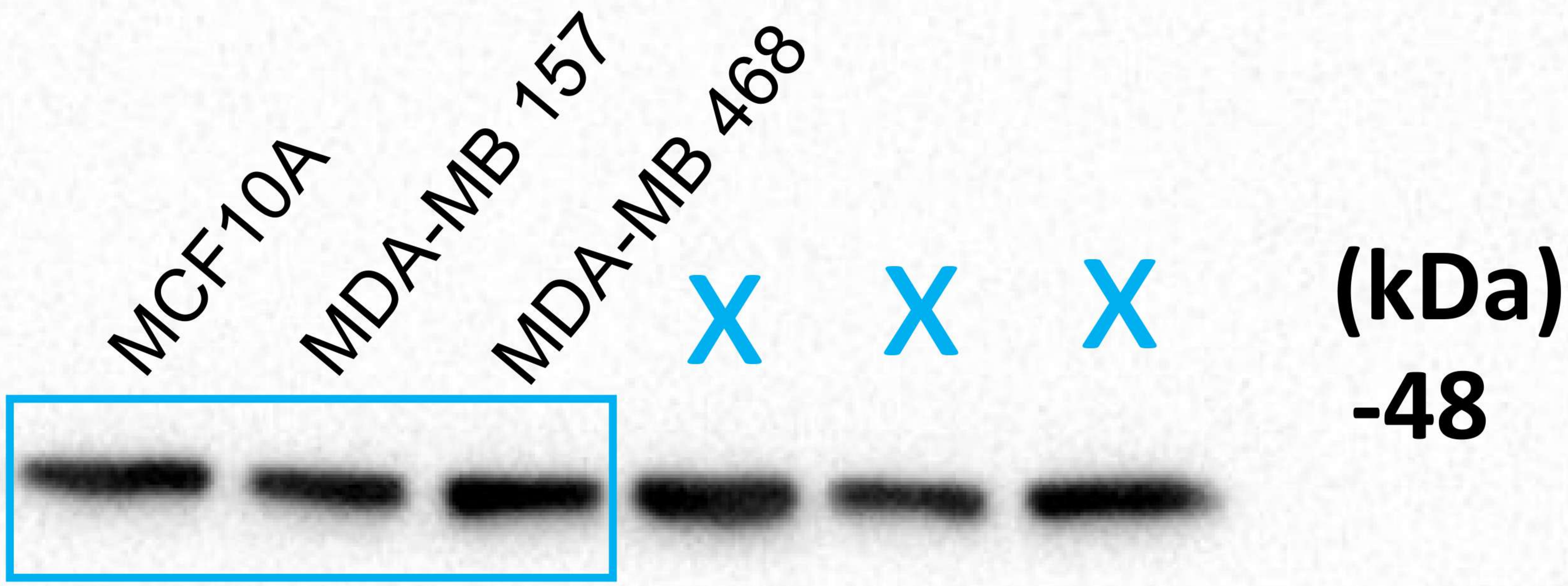

Figure 3B ALDH1A3 (MDA-MB 157)

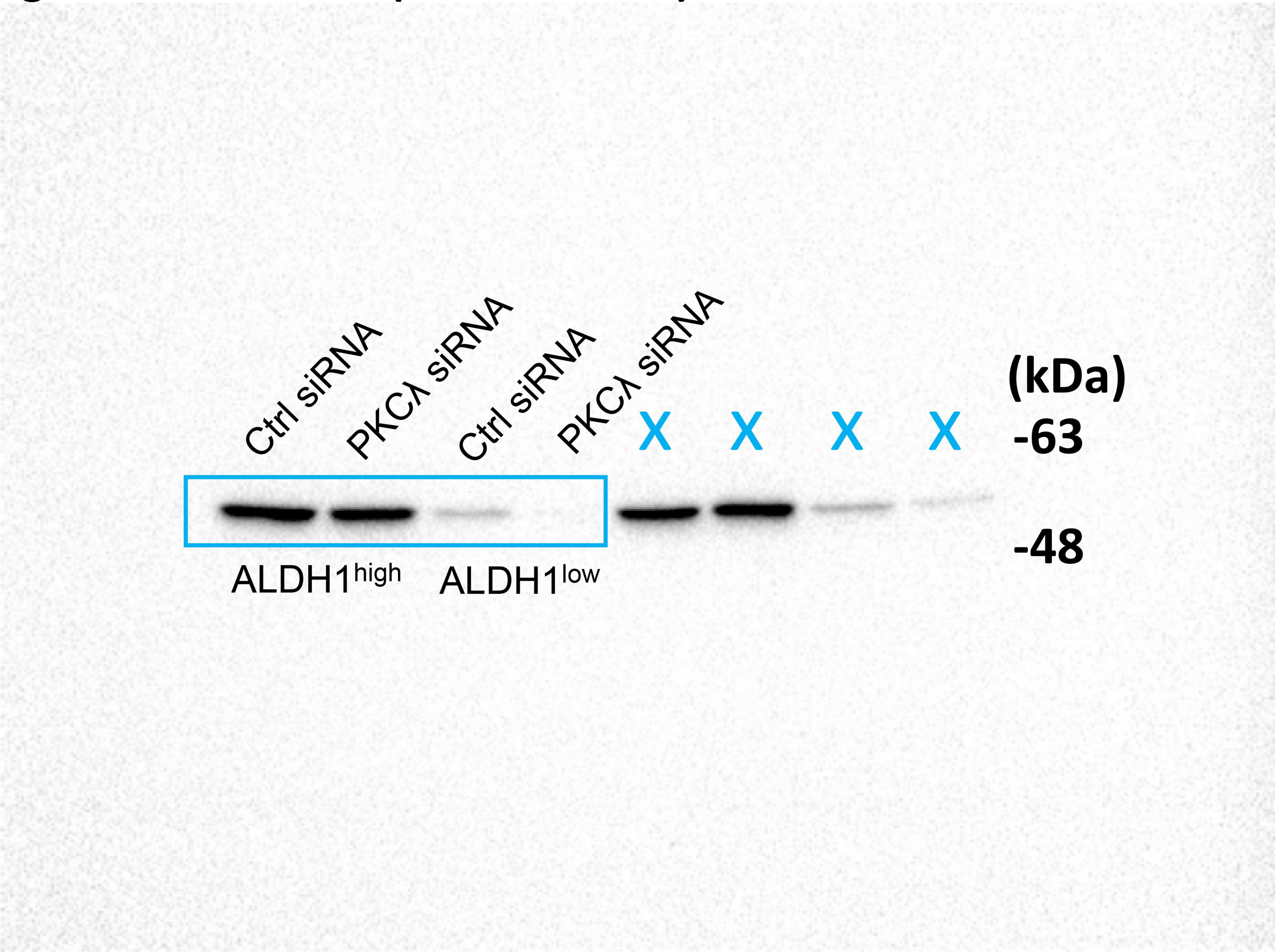

Figure 3B PKCλ (MDA-MB 157)

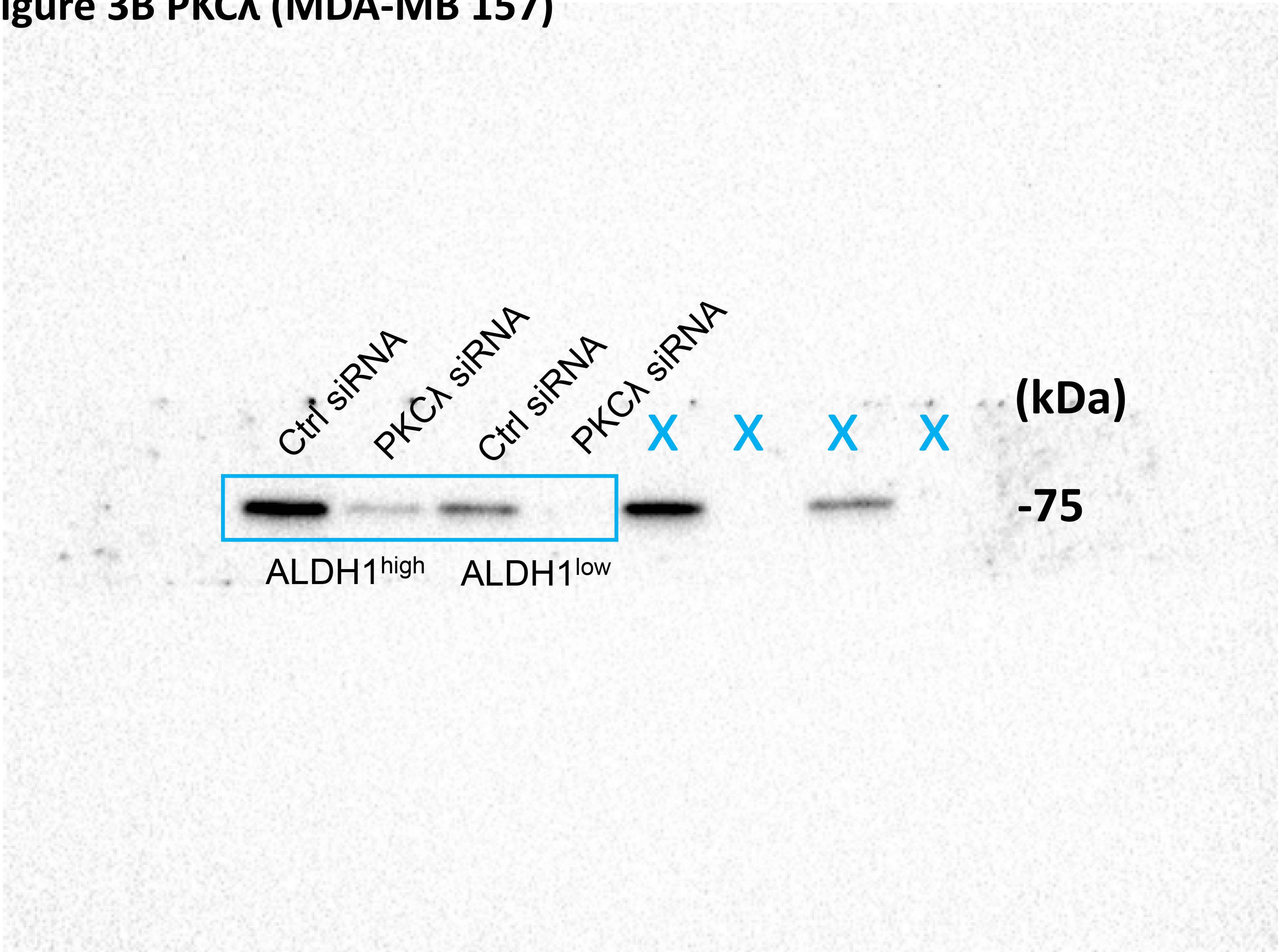

Figure 3B β-actin (MDA-MB 157)

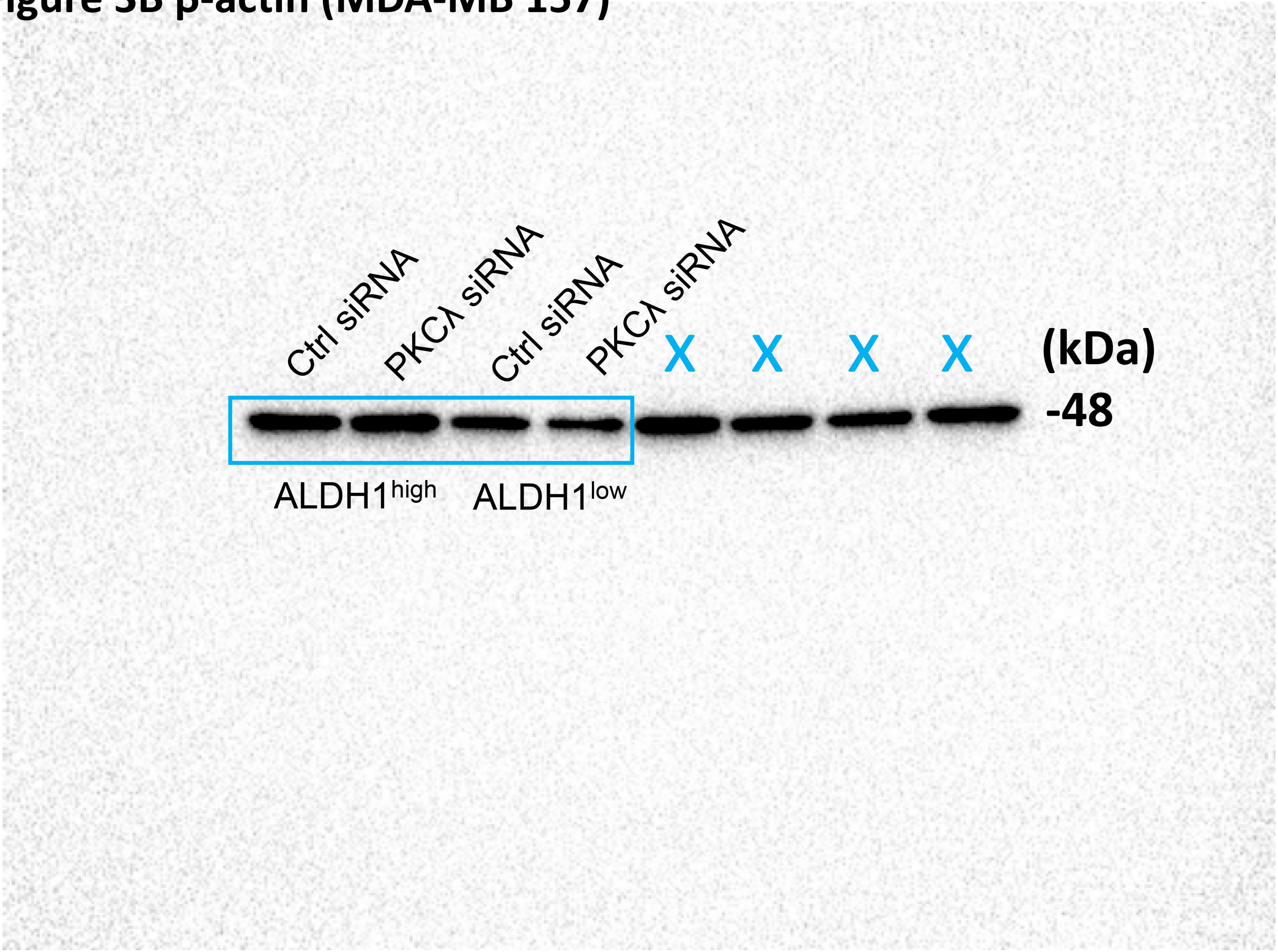

Figure 3B ALDH1A3 (MDA-MB 468)

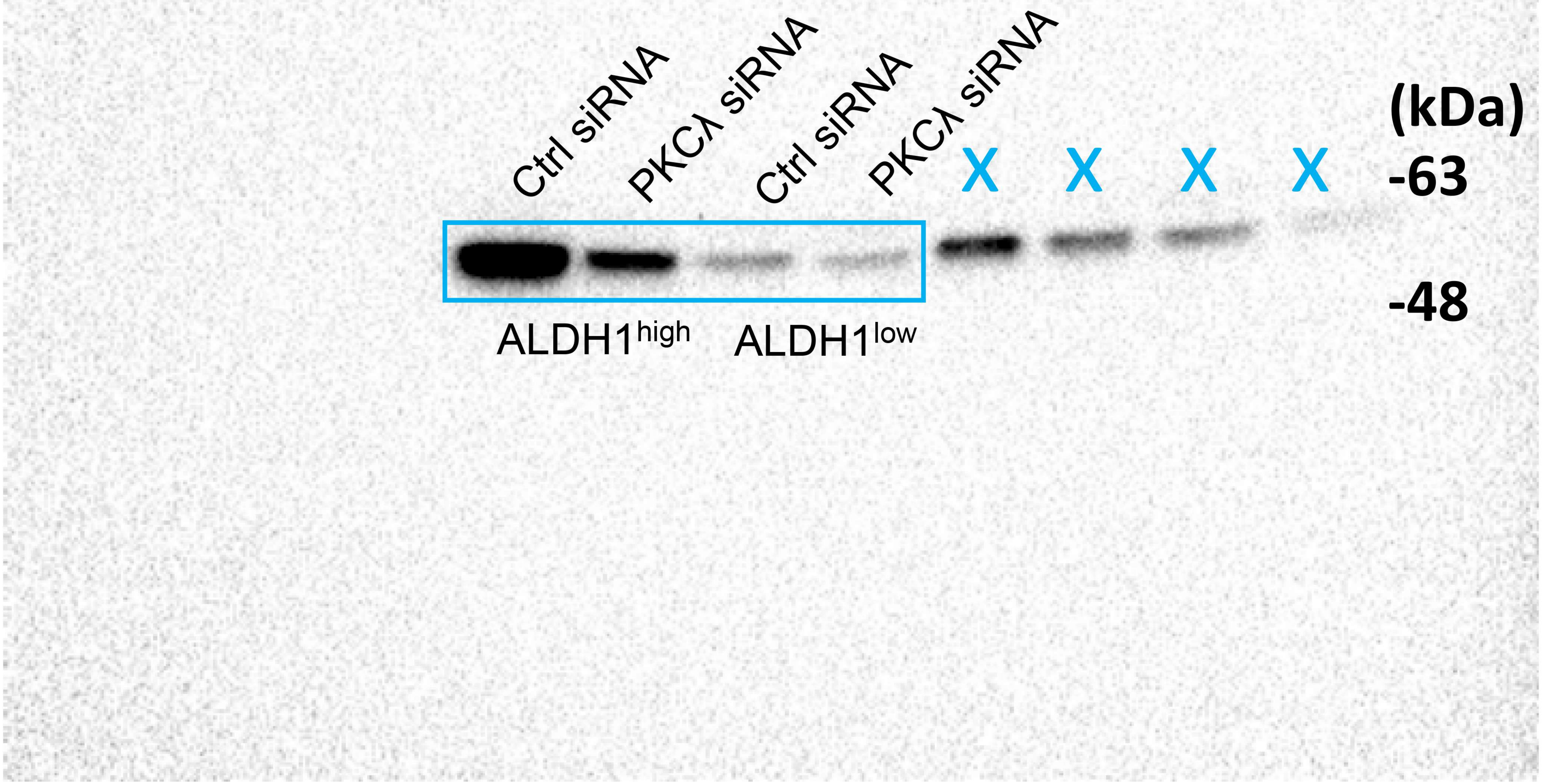

Figure 3B PKCλ (MDA-MB 468)

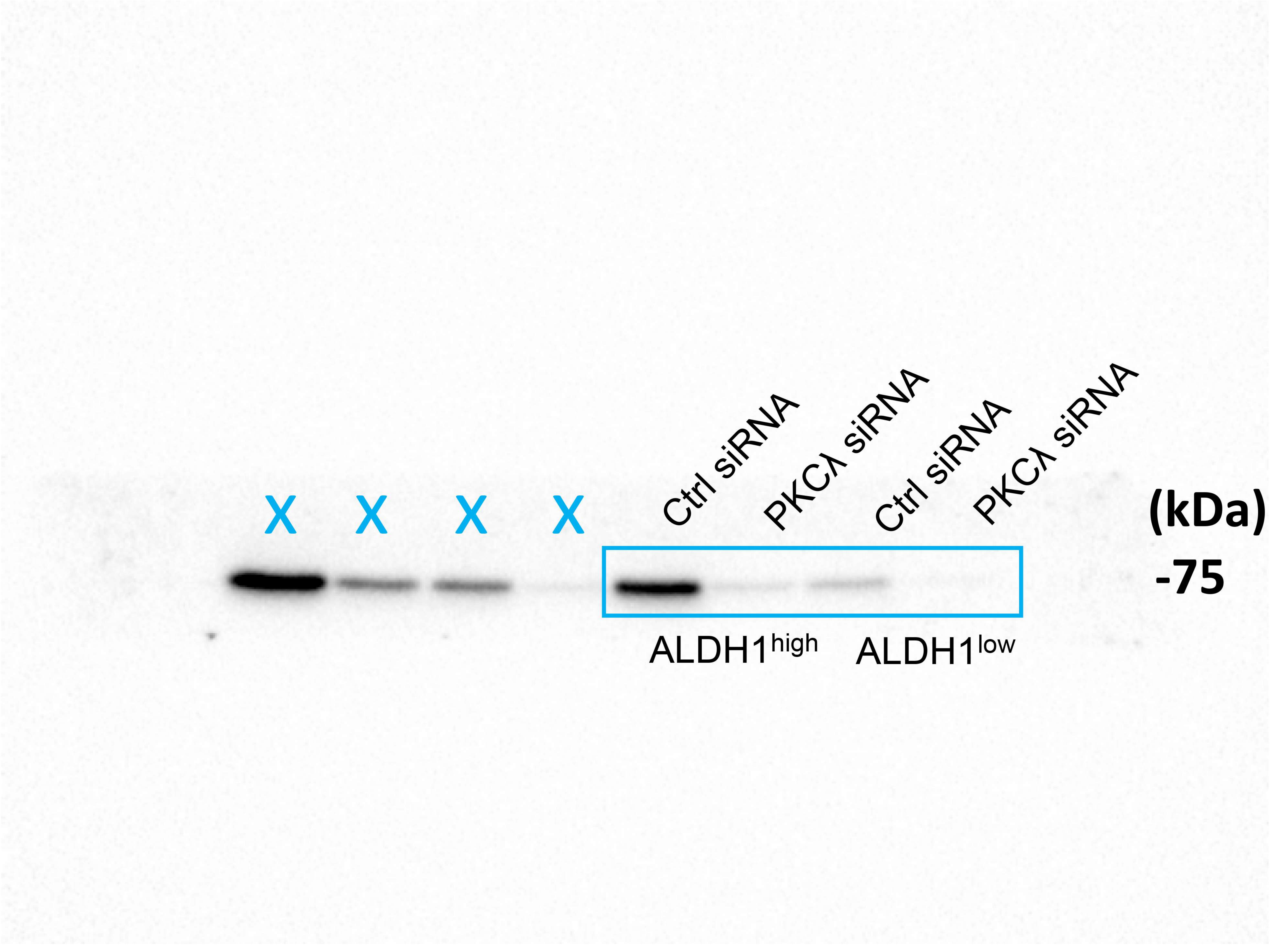

Figure 3B β-actin (MDA-MB 468)

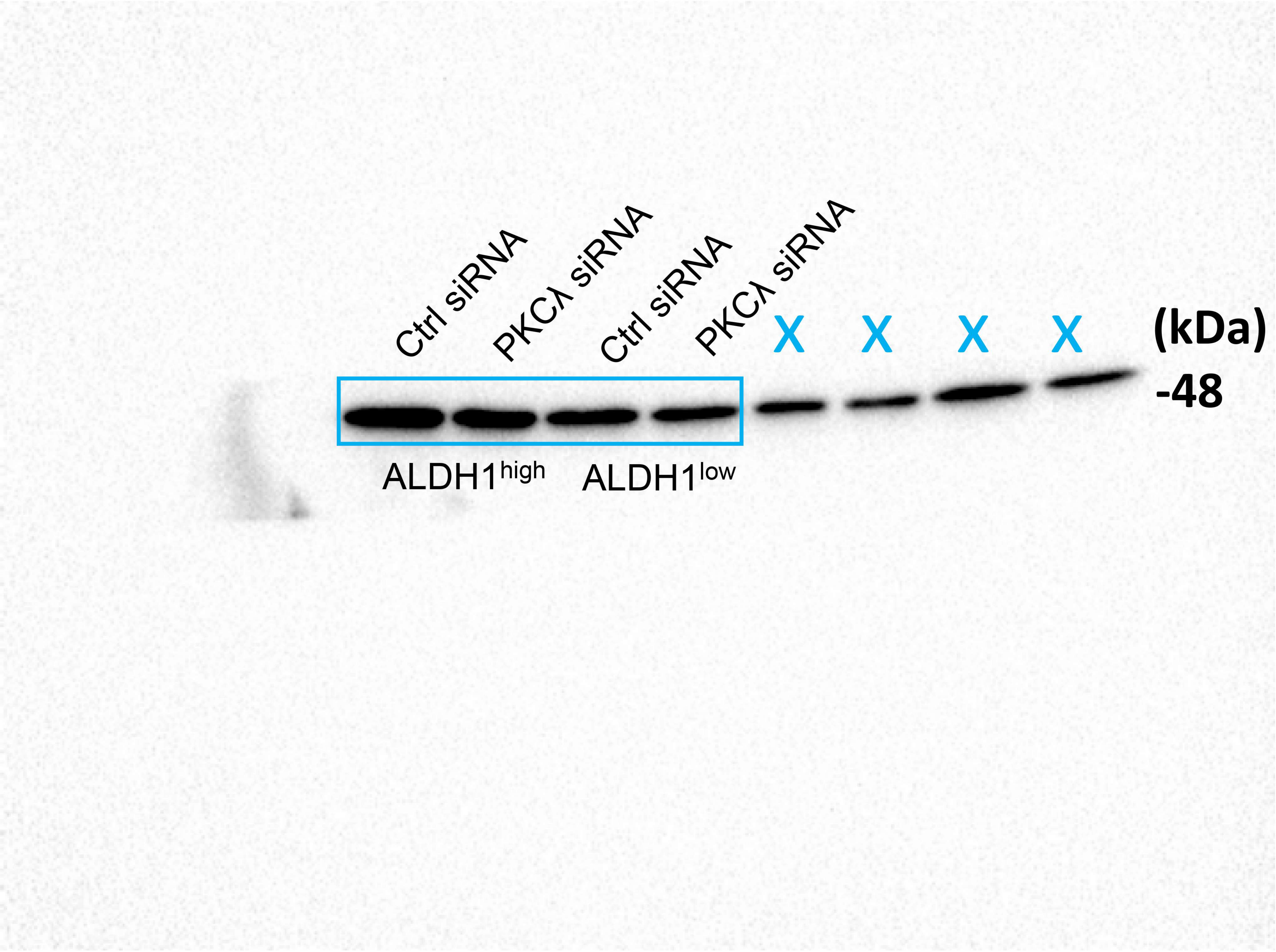

Figure 3C ALDH1A3

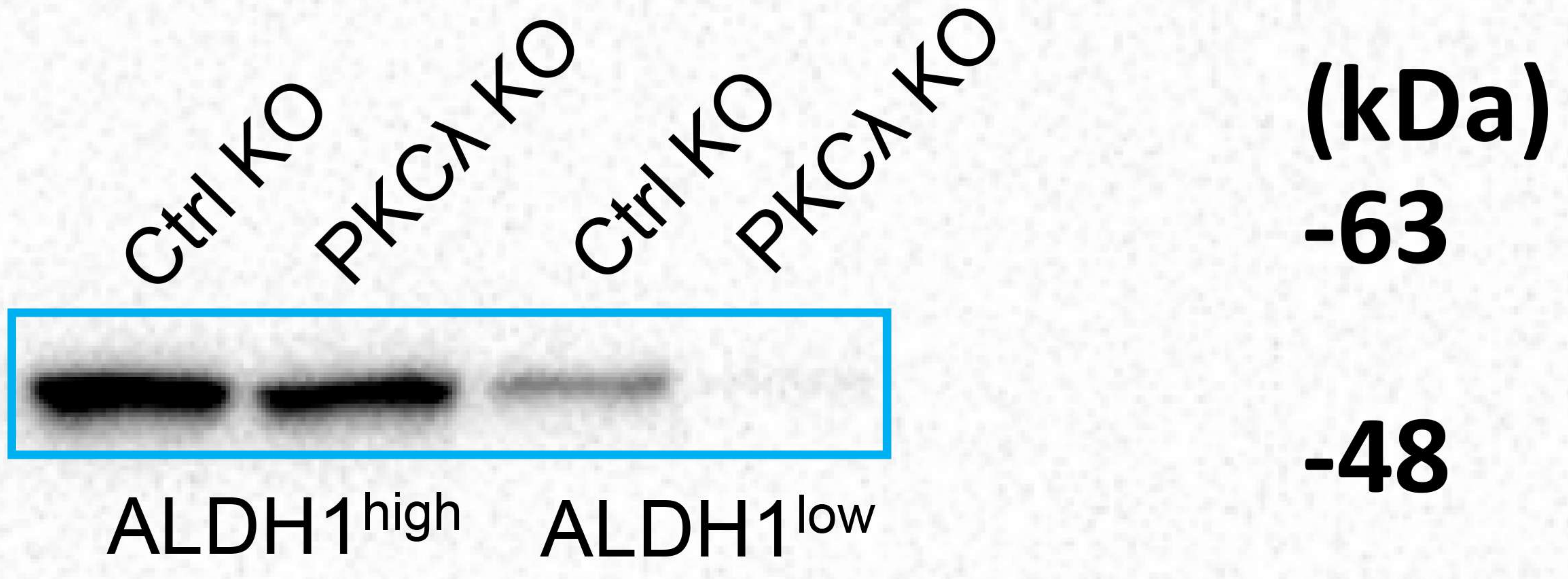

Figure 3C PKCλ

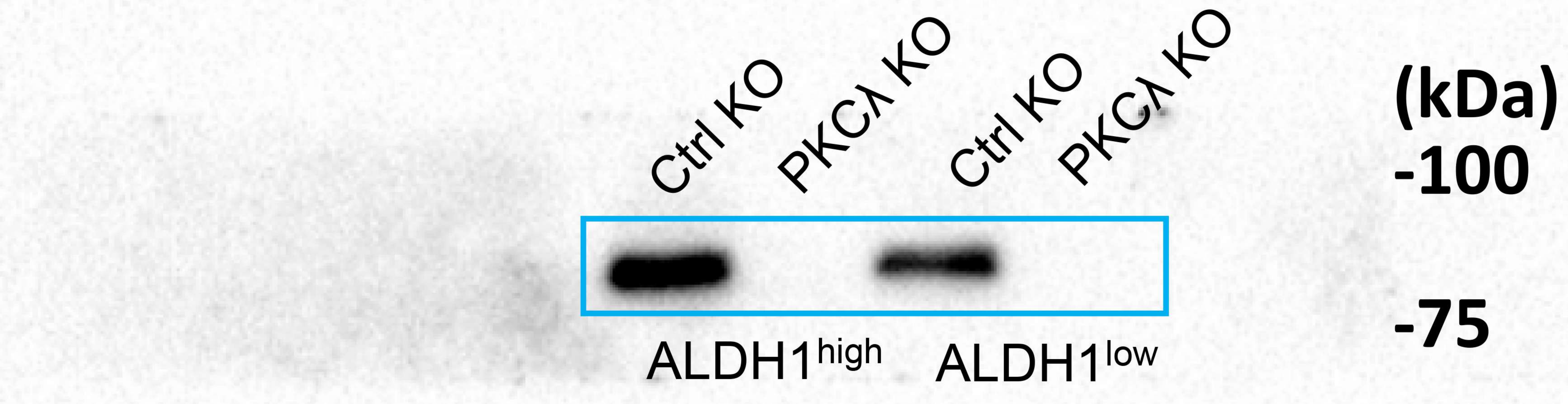

Figure 3C β-actin

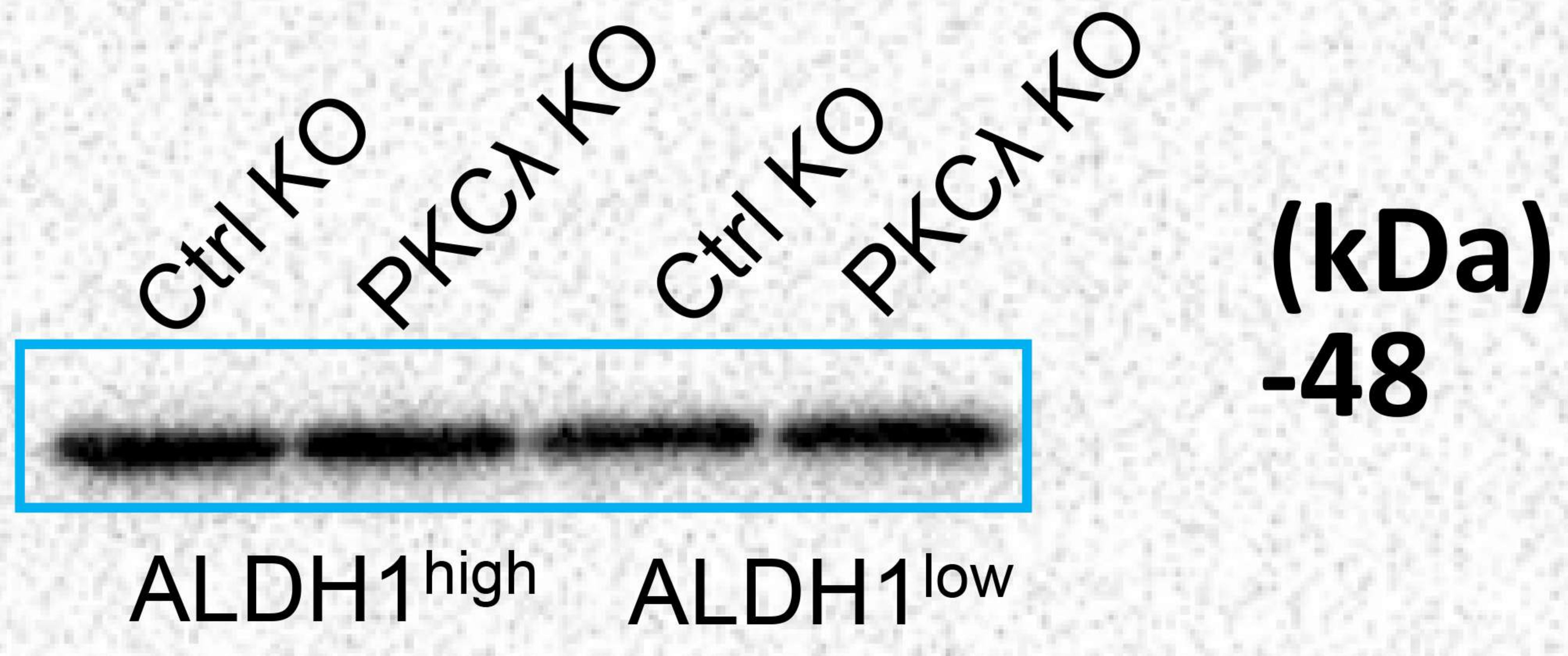

Figure 4B anti-Caspase-3 (intact caspase3) (MDA-MB 157)

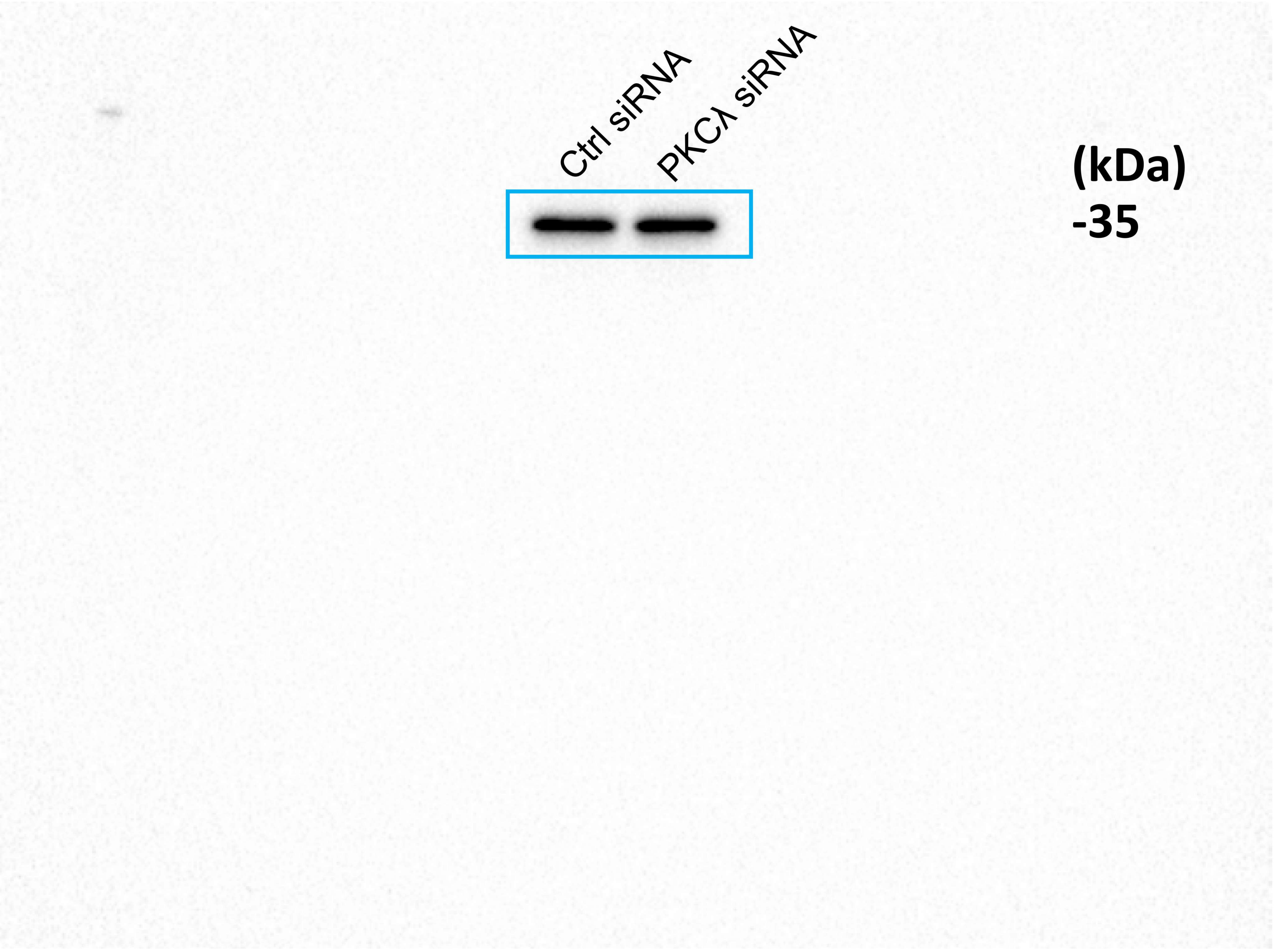

Figure 4B anti-Caspase-3 (cleaved caspase-3) (MDA-MB 157)

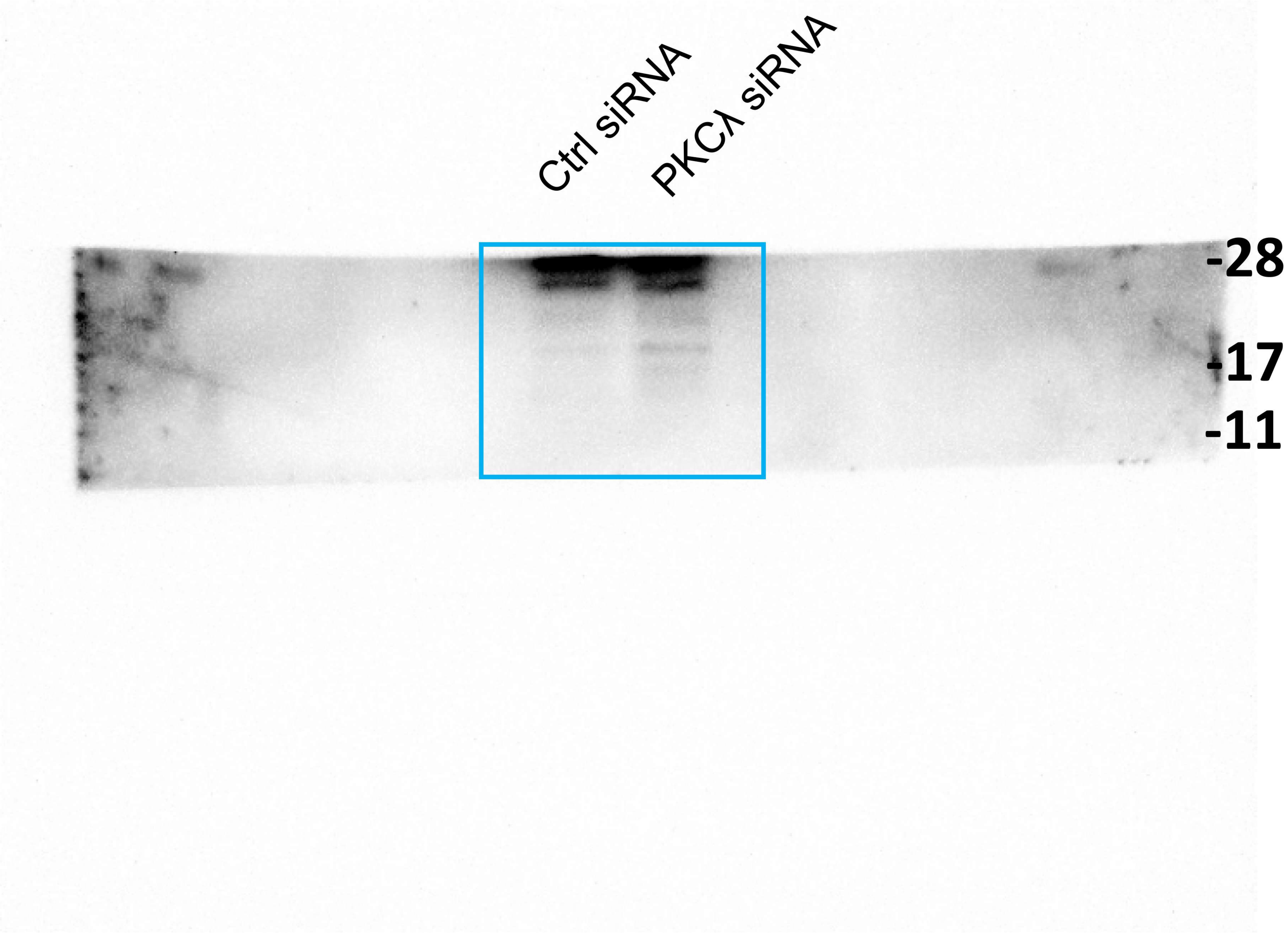

Figure 4B anti-Cleaved caspase-3 (MDA-MB 157)

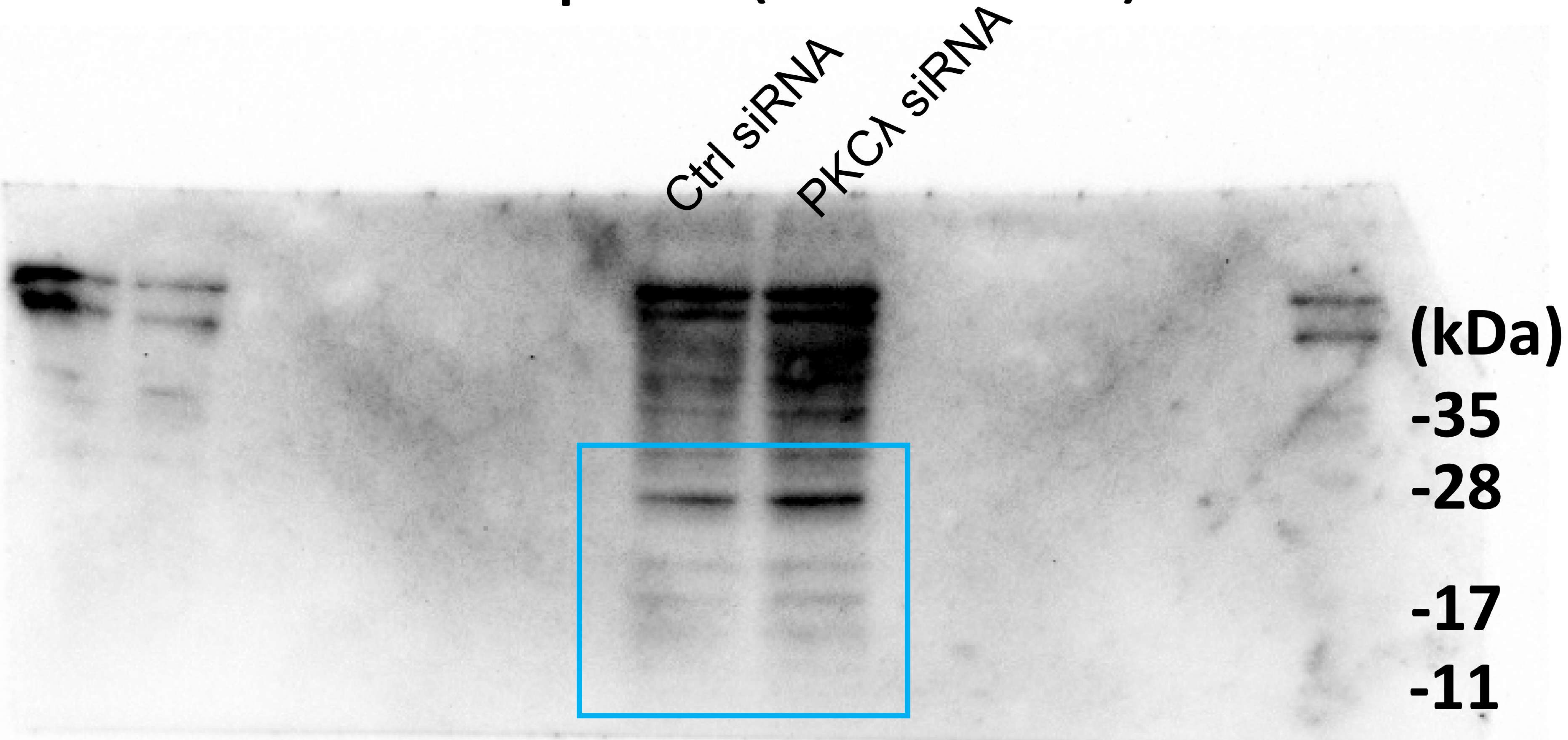

Figure 4B β-actin (MDA-MB 157)

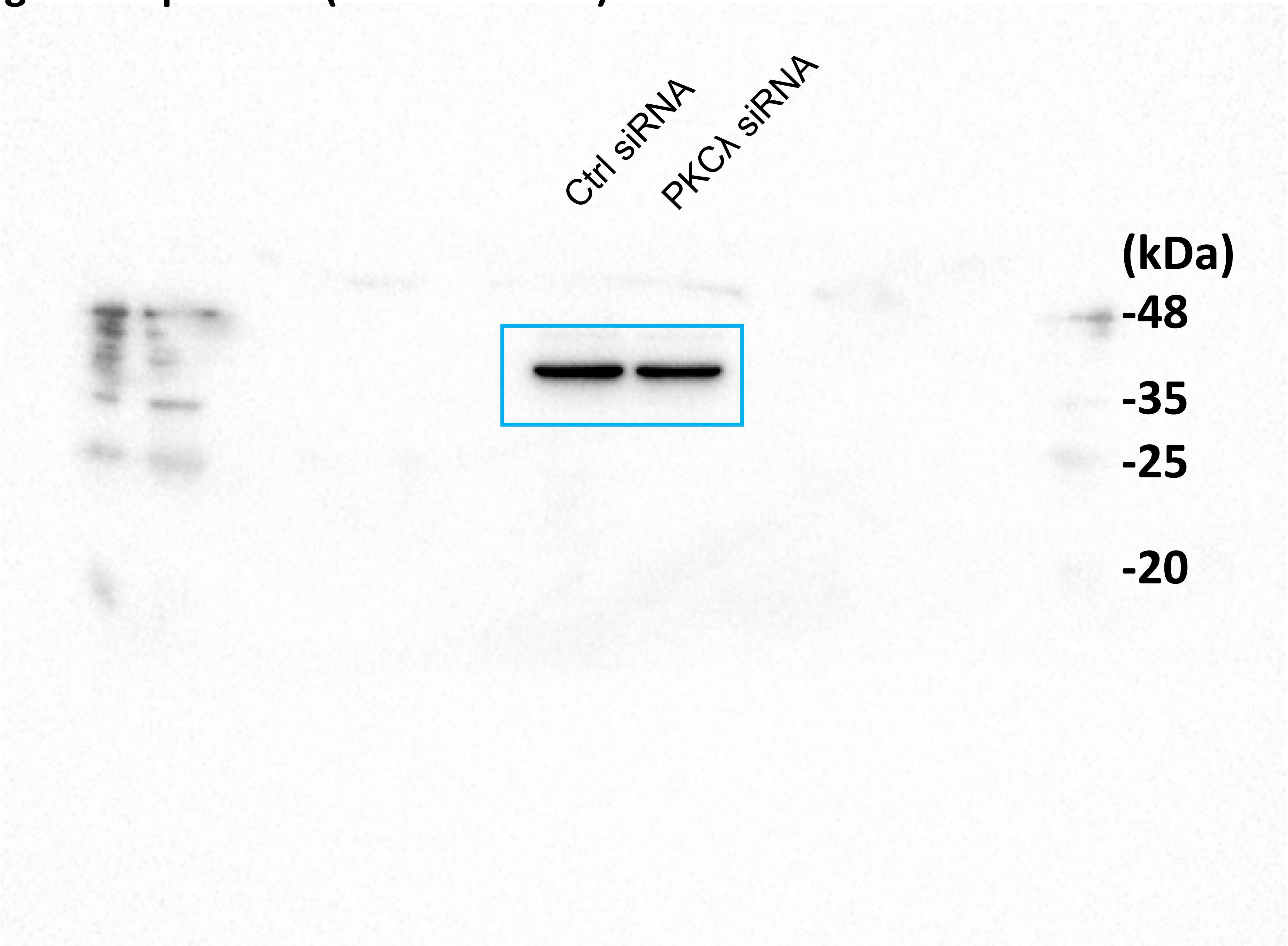

Figure 4B anti-Caspase-3 (intact caspase3) (MDA-MB 468)

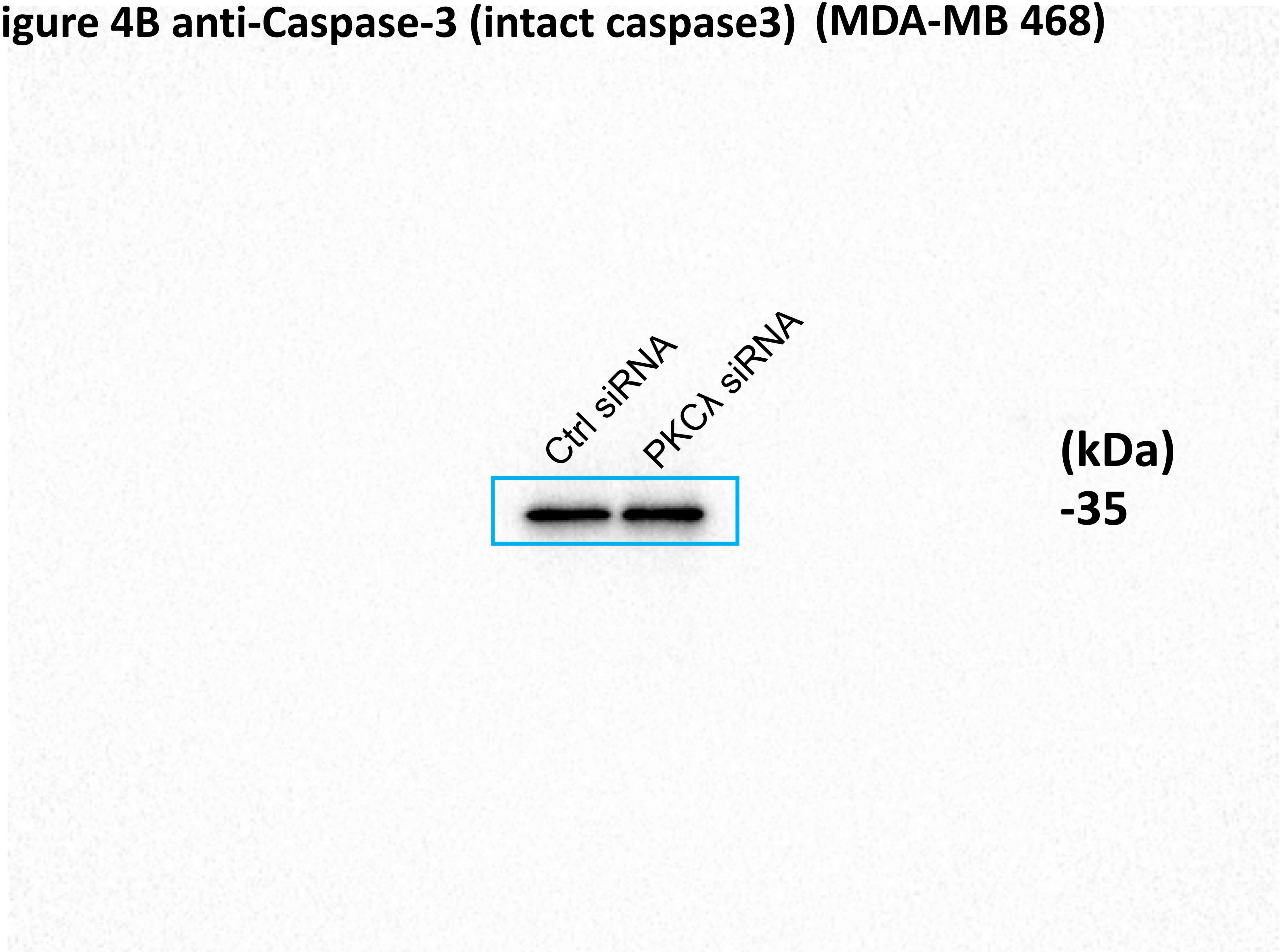

Figure 4B anti-Caspase-3 (cleaved caspase3)(MDA-MB 468)

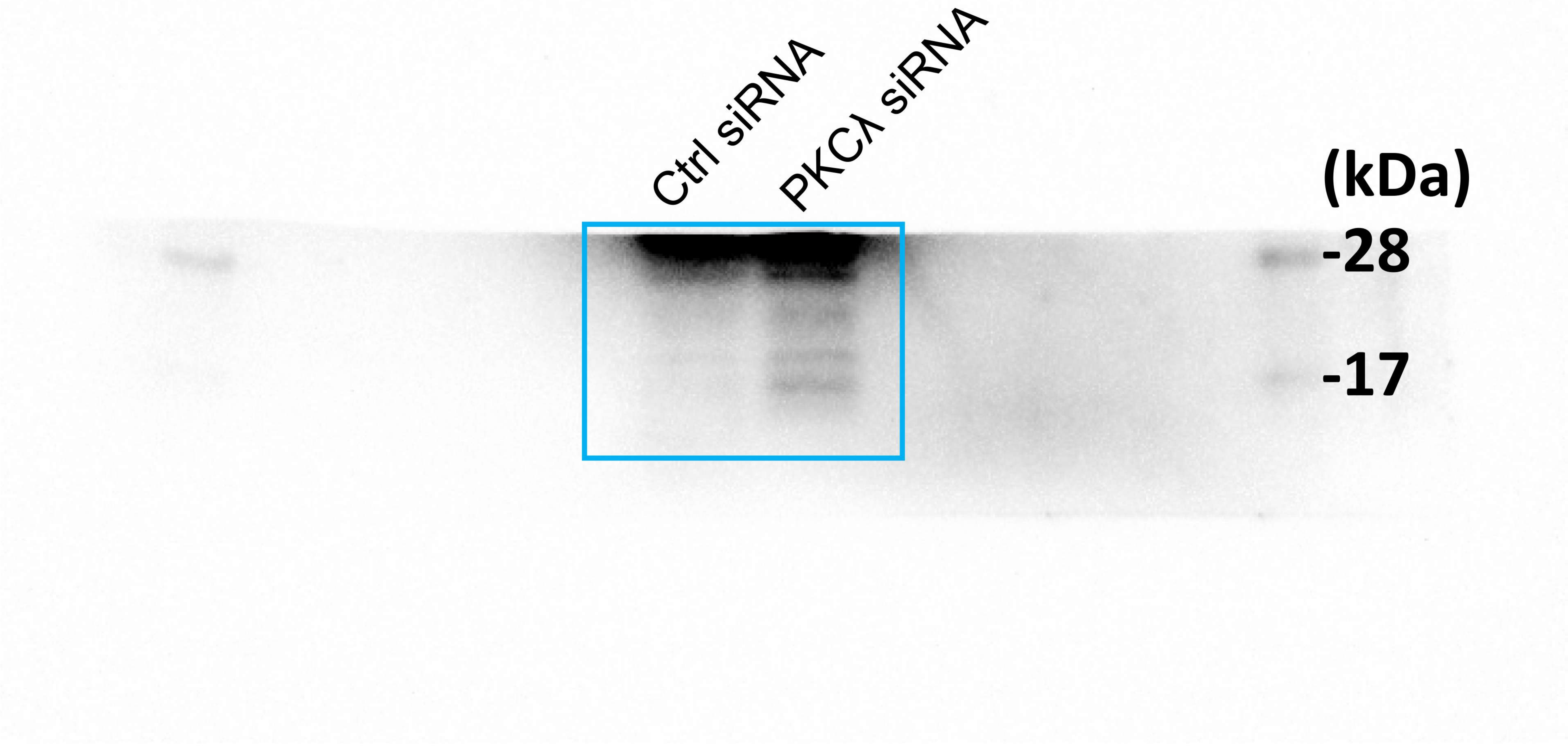

Figure 4B anti-Cleaved caspase-3 (MDA-MB 468)

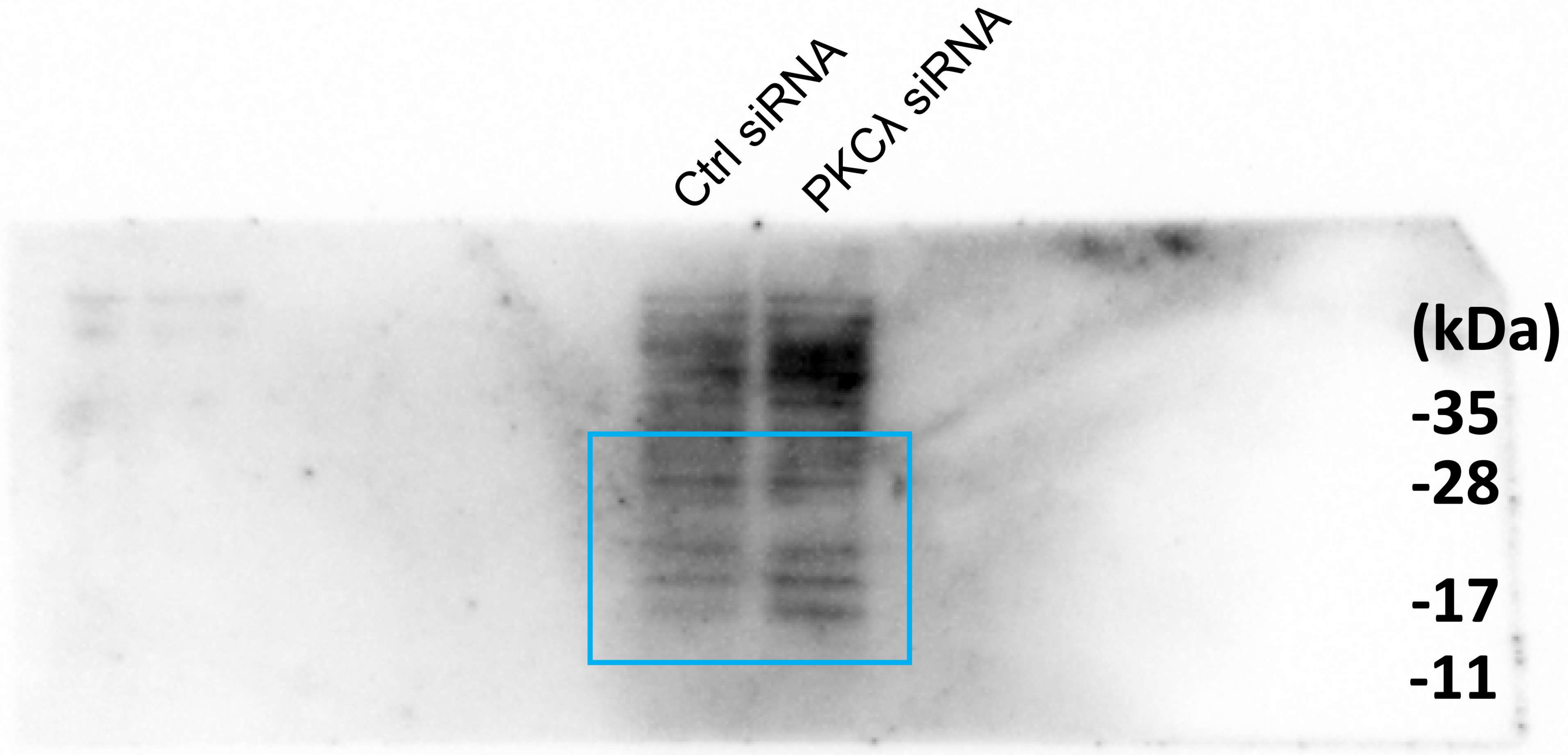

Figure 4B β-actin (MDA-MB 468)

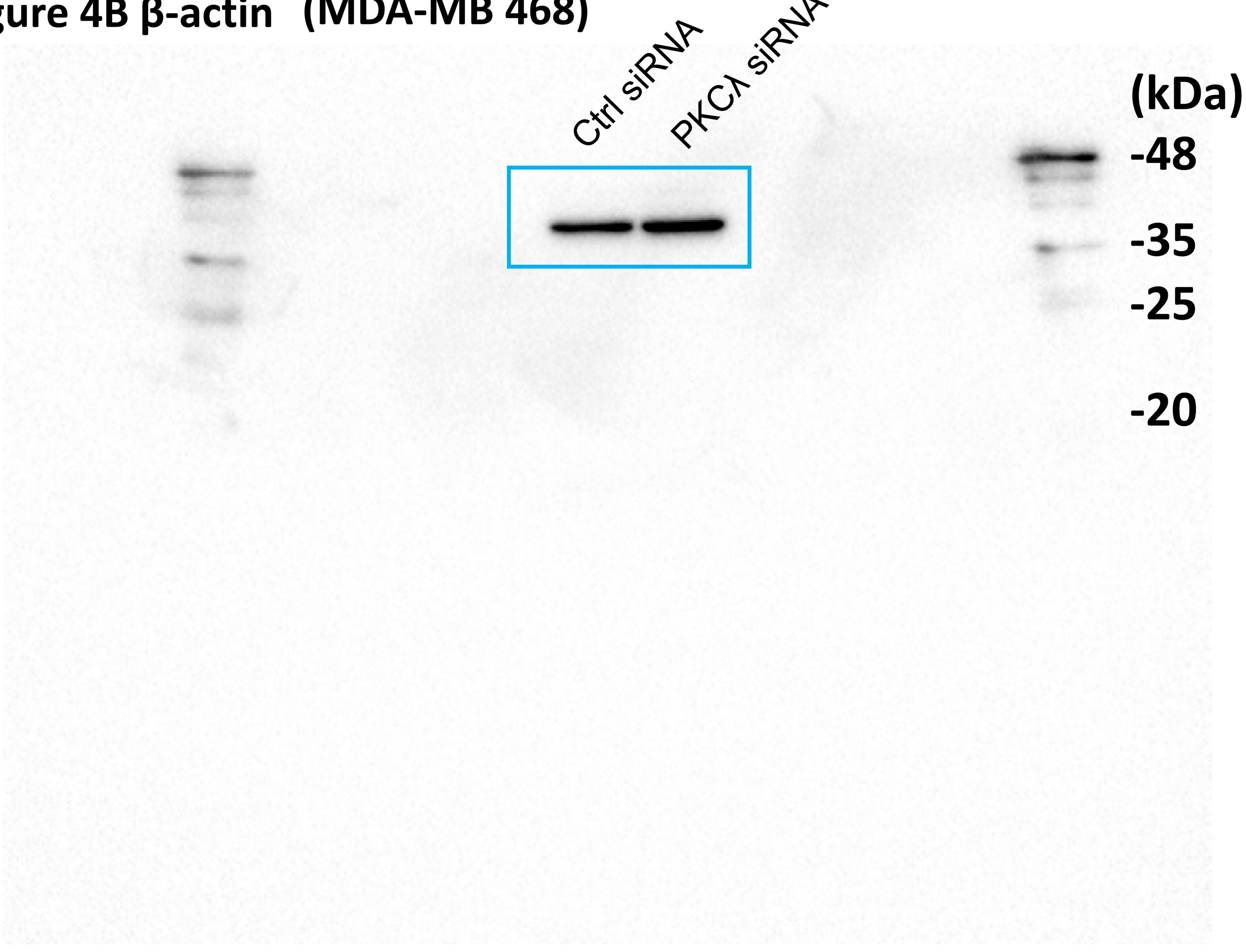

Figure 4E Caspase-3 (2D culture condition)

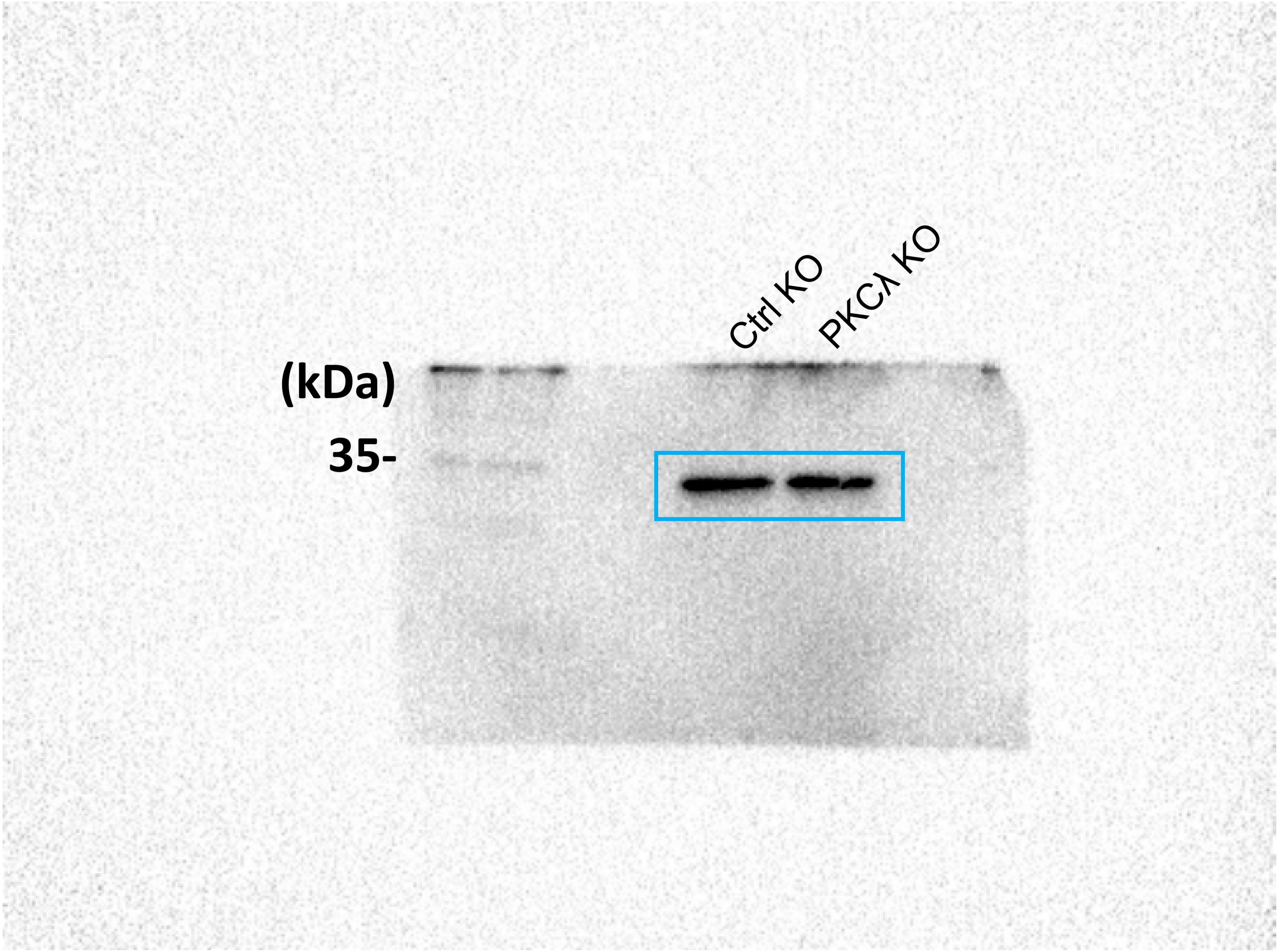

Figure 4E Cleaved caspase-3 (2D culture condition)

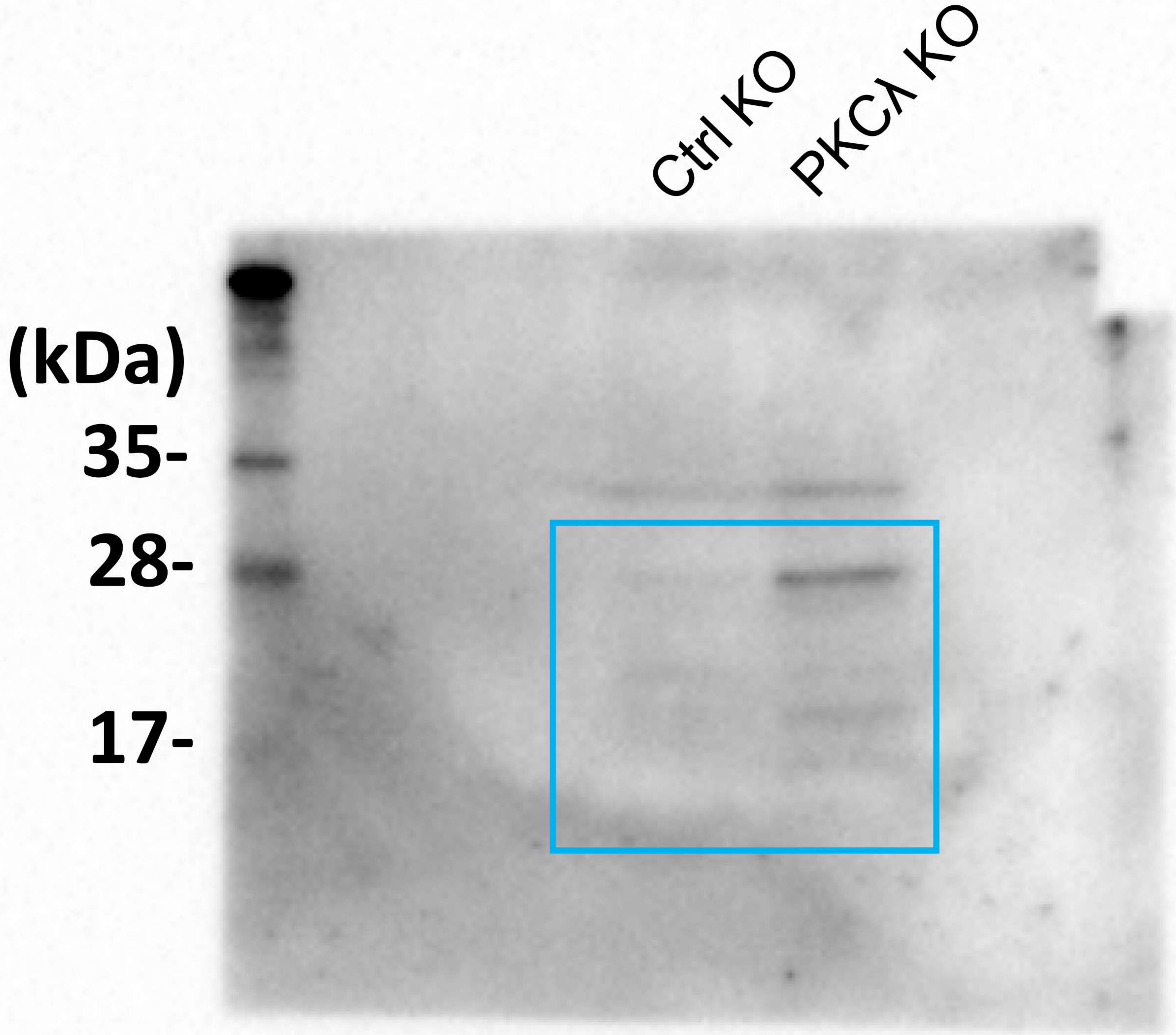

Figure 4E β-actin (2D culture condition)

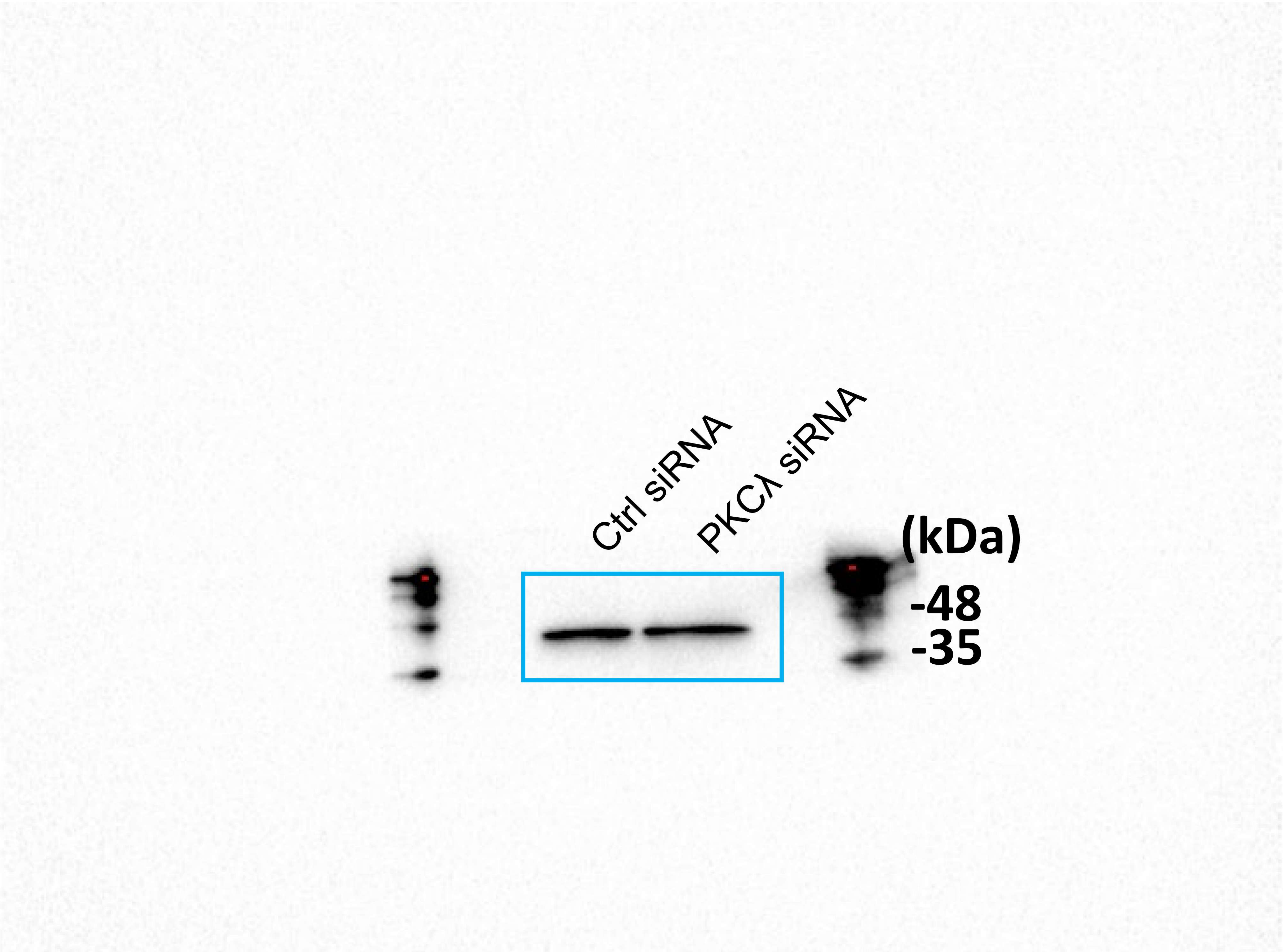

Figure 4E Caspase-3 (Tumor-sphere culture condition)

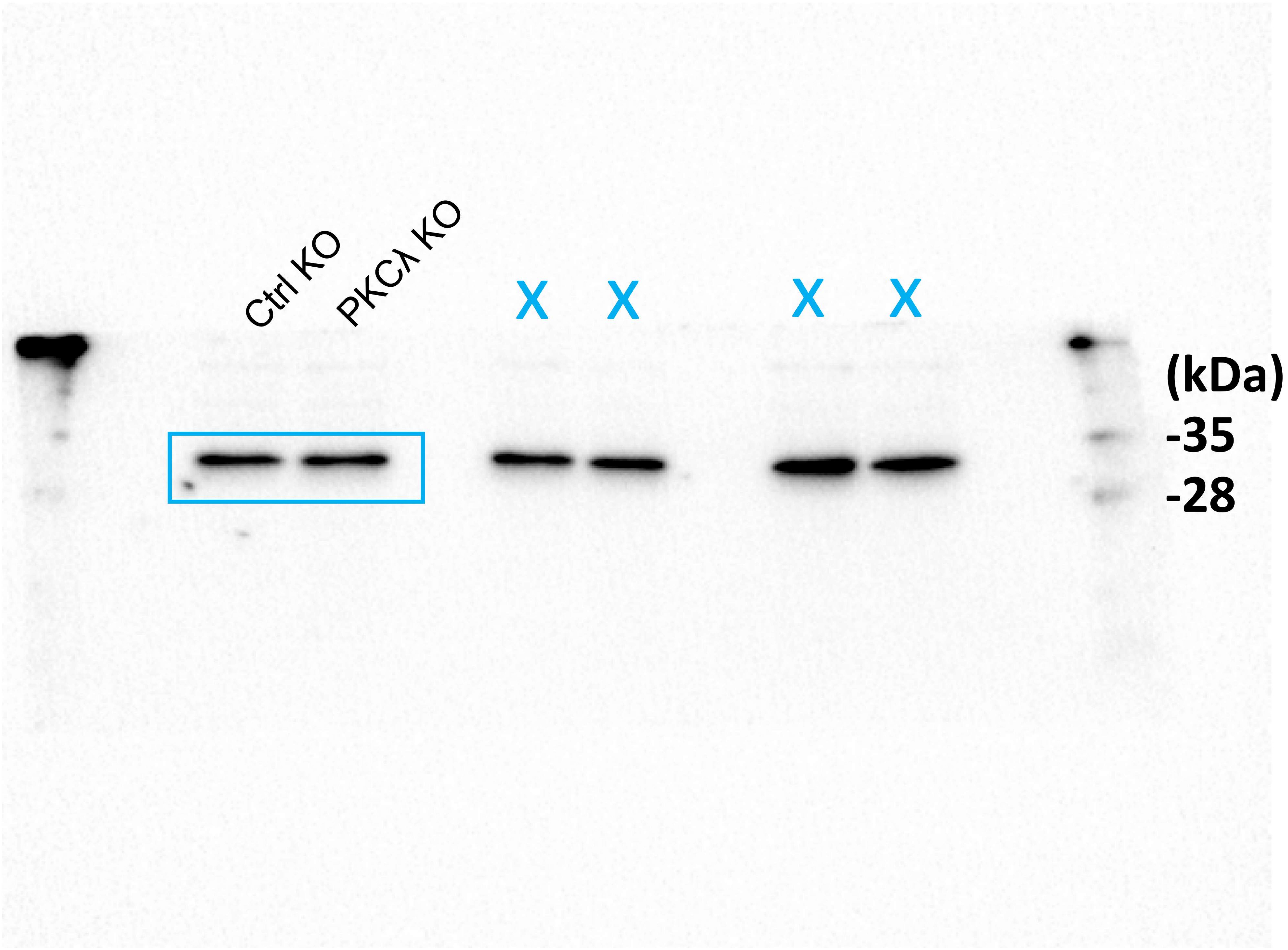

Figure 4E Cleaved caspase-3 (Tumor-sphere culture condition)

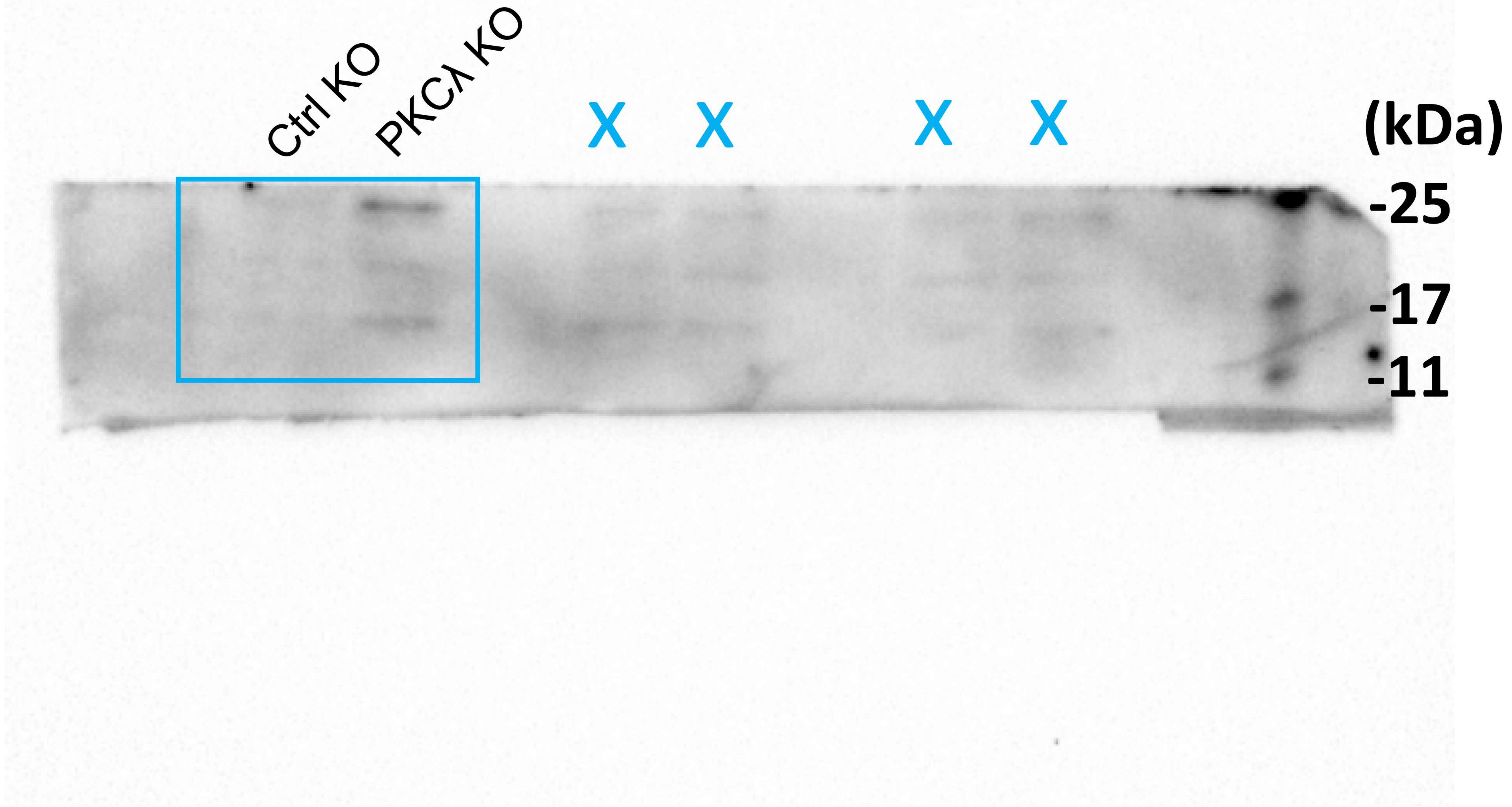

Figure 4E β-actin (Tumor-sphere culture condition)

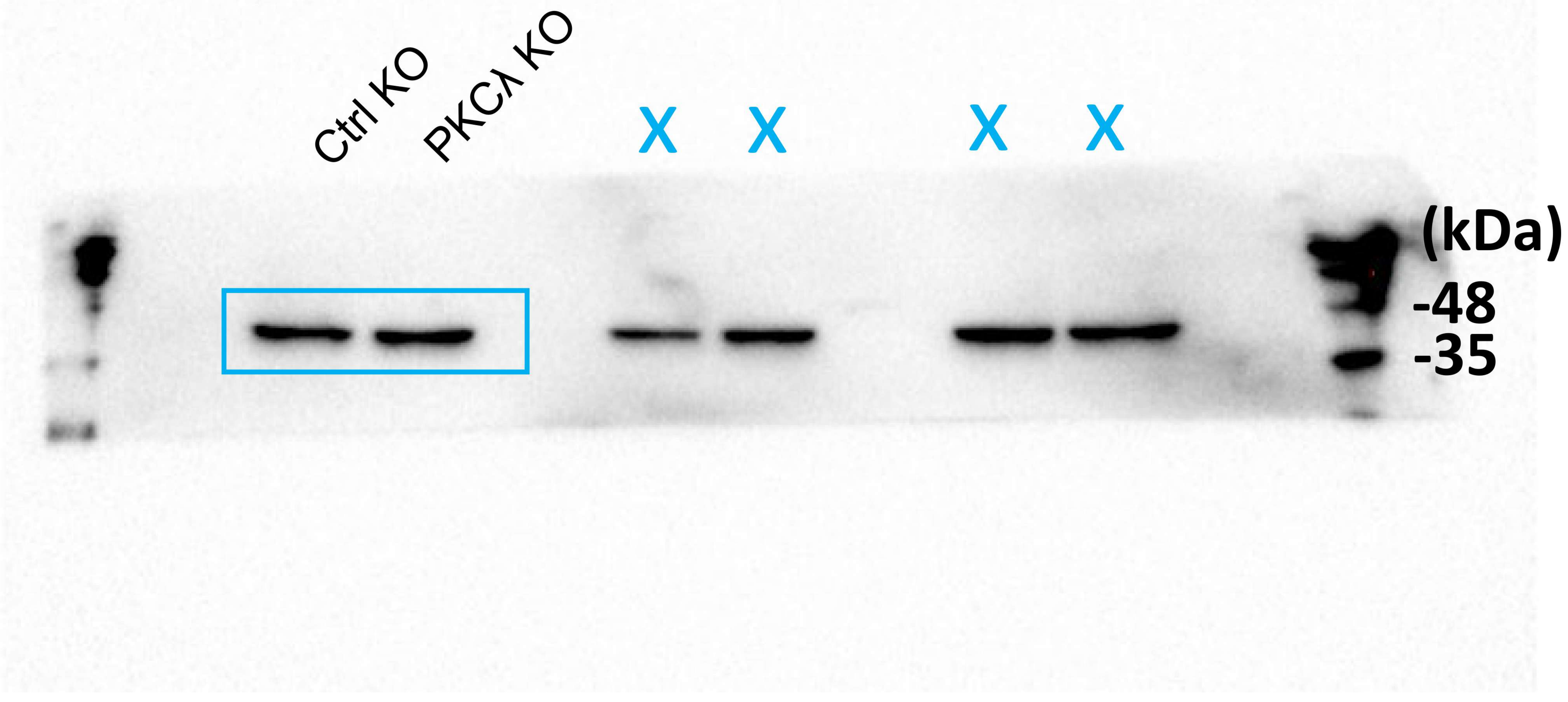

Figure 4G Caspase-3

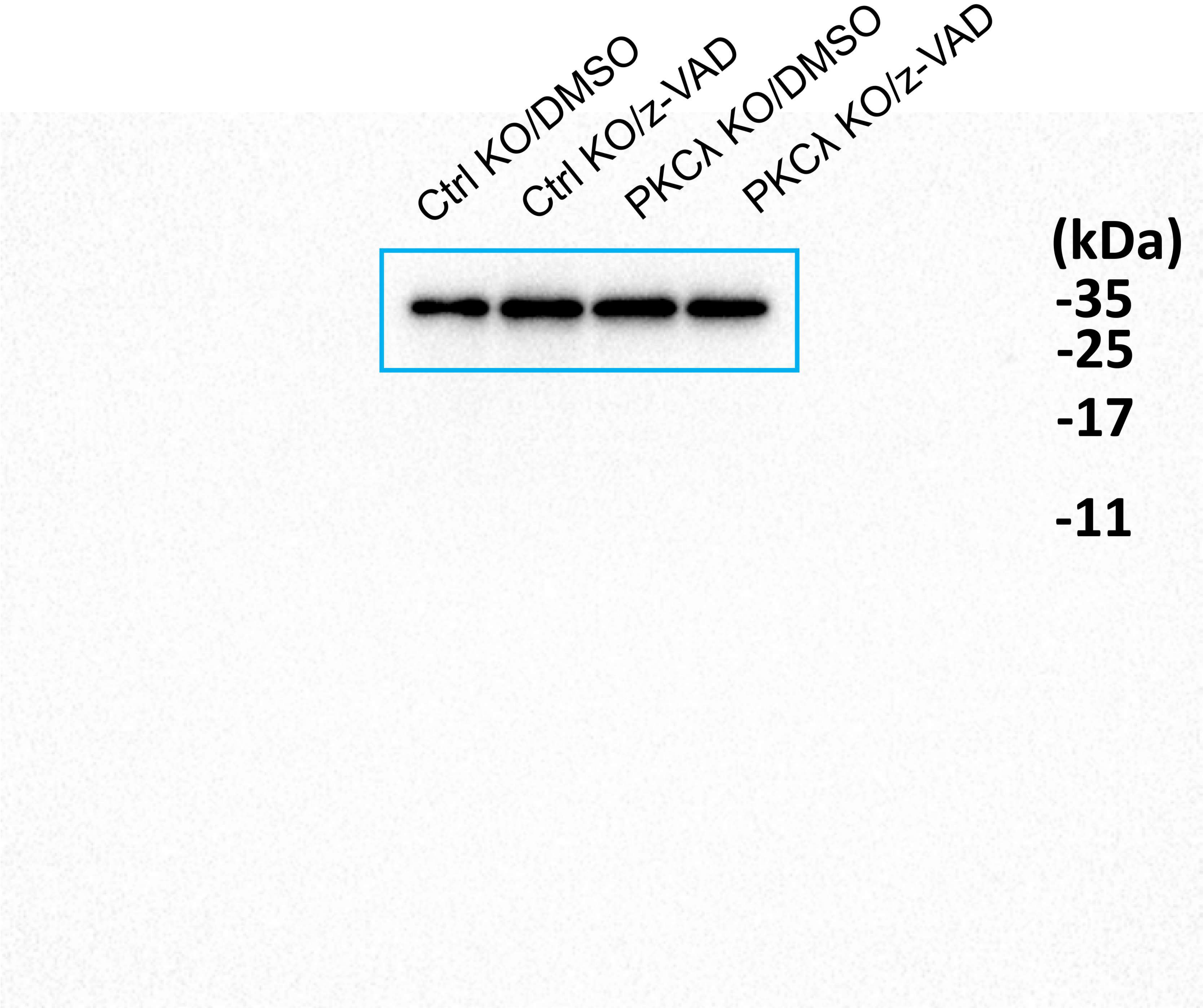

Figure 4G Cleaved Caspase-3

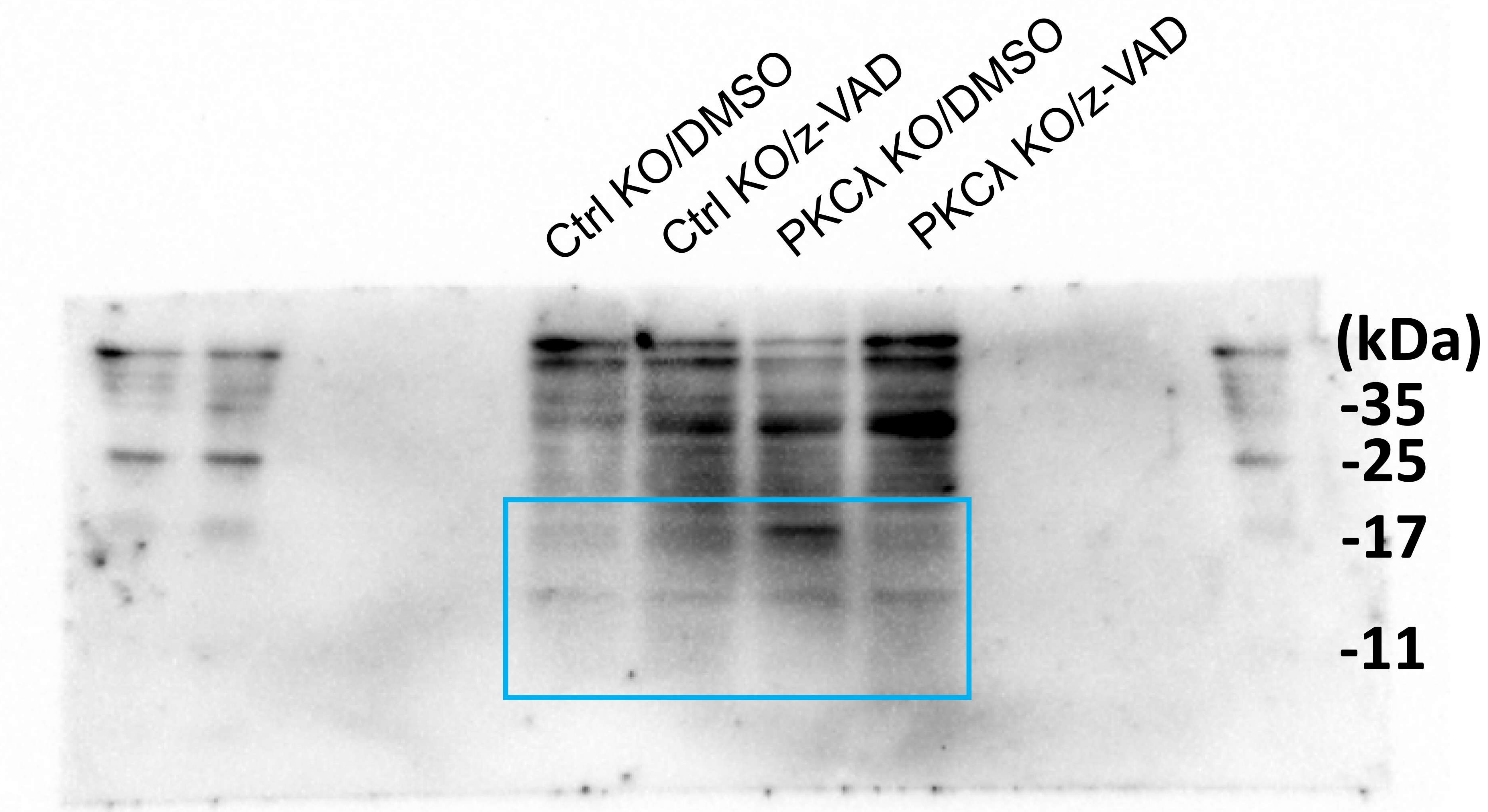

Figure 4G β-actin

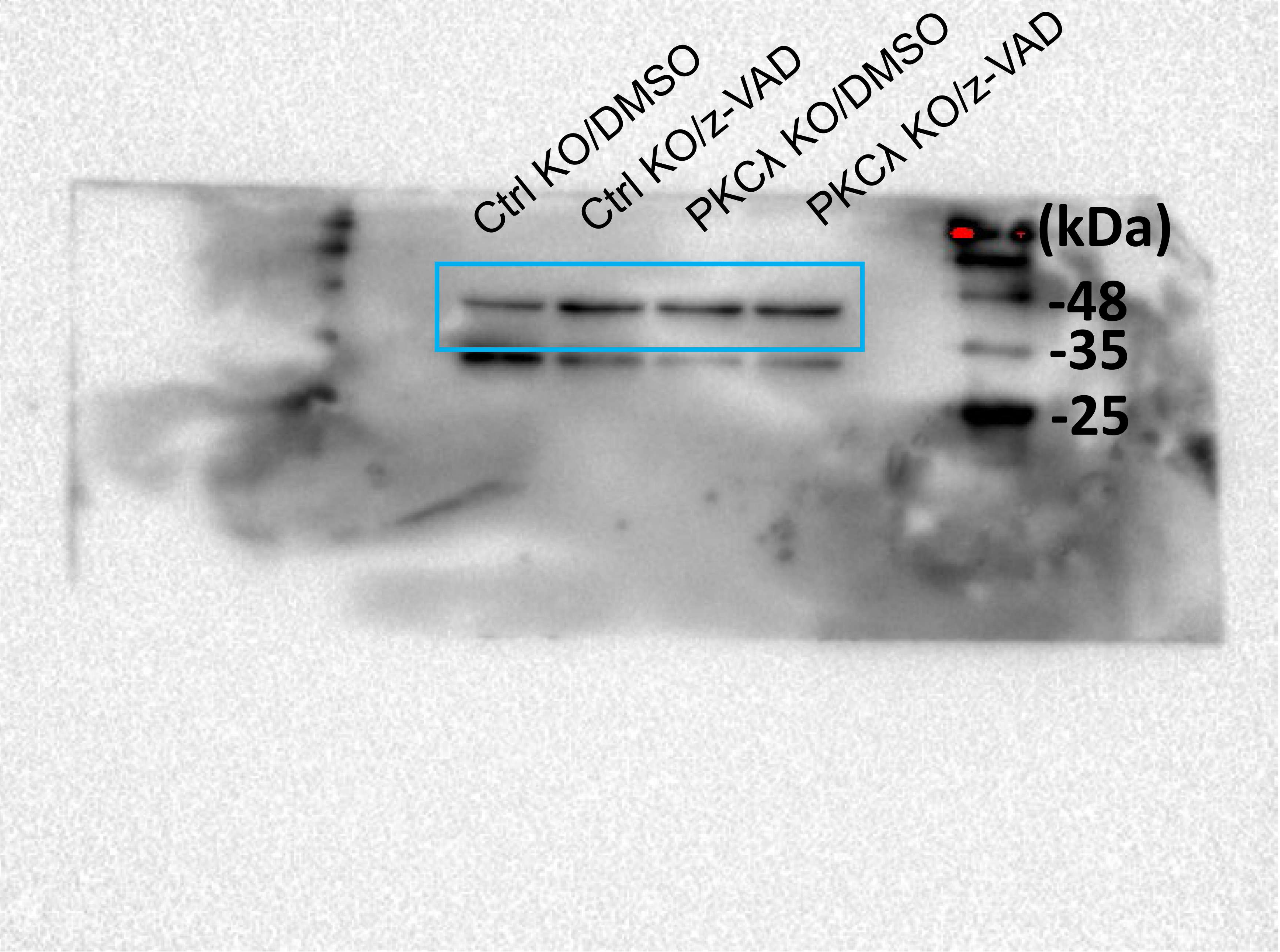

Figure S3A pS473-Akt1 (MDA-MB 157)

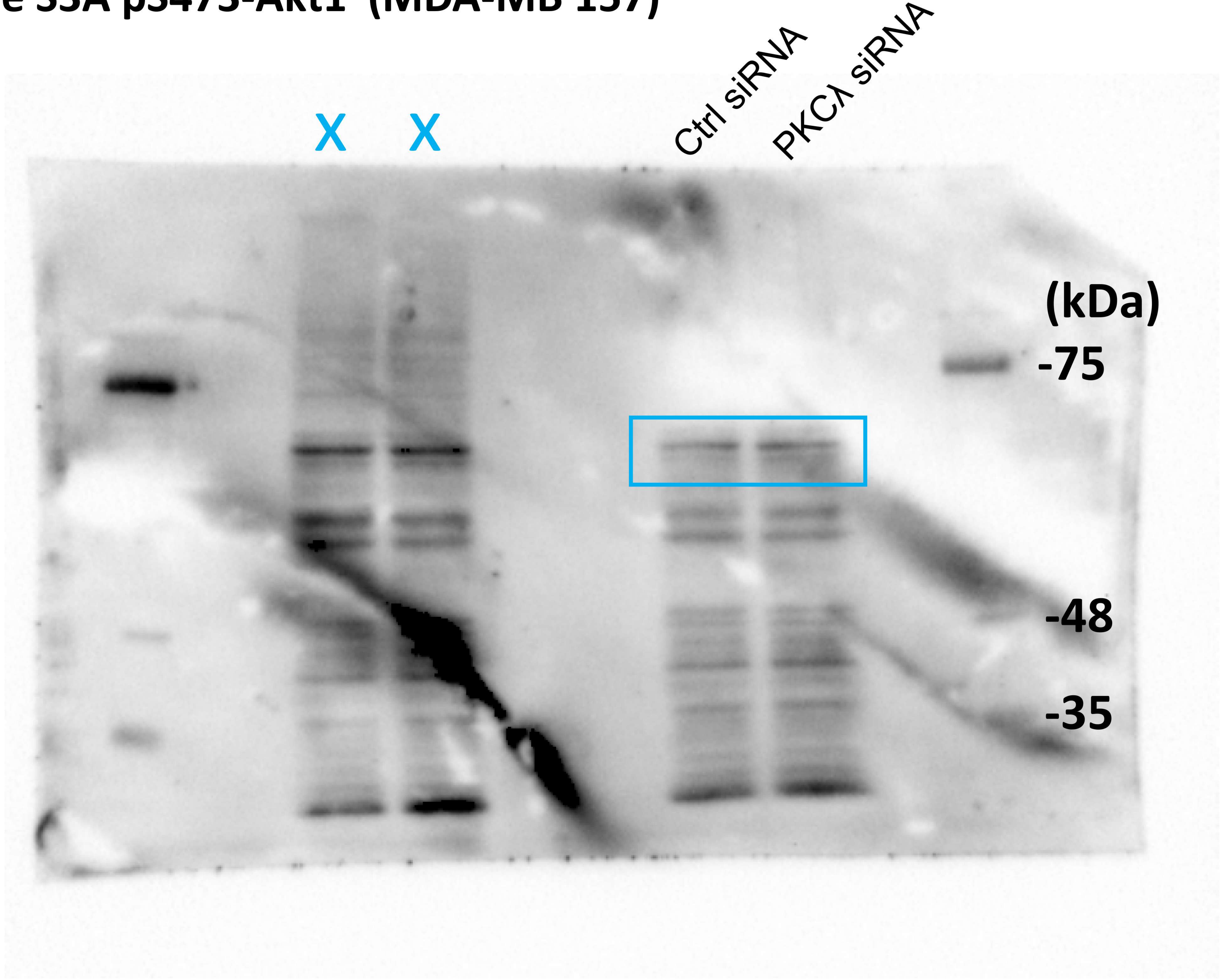

Figure S3A pT308-Akt1 (MDA-MB 157)

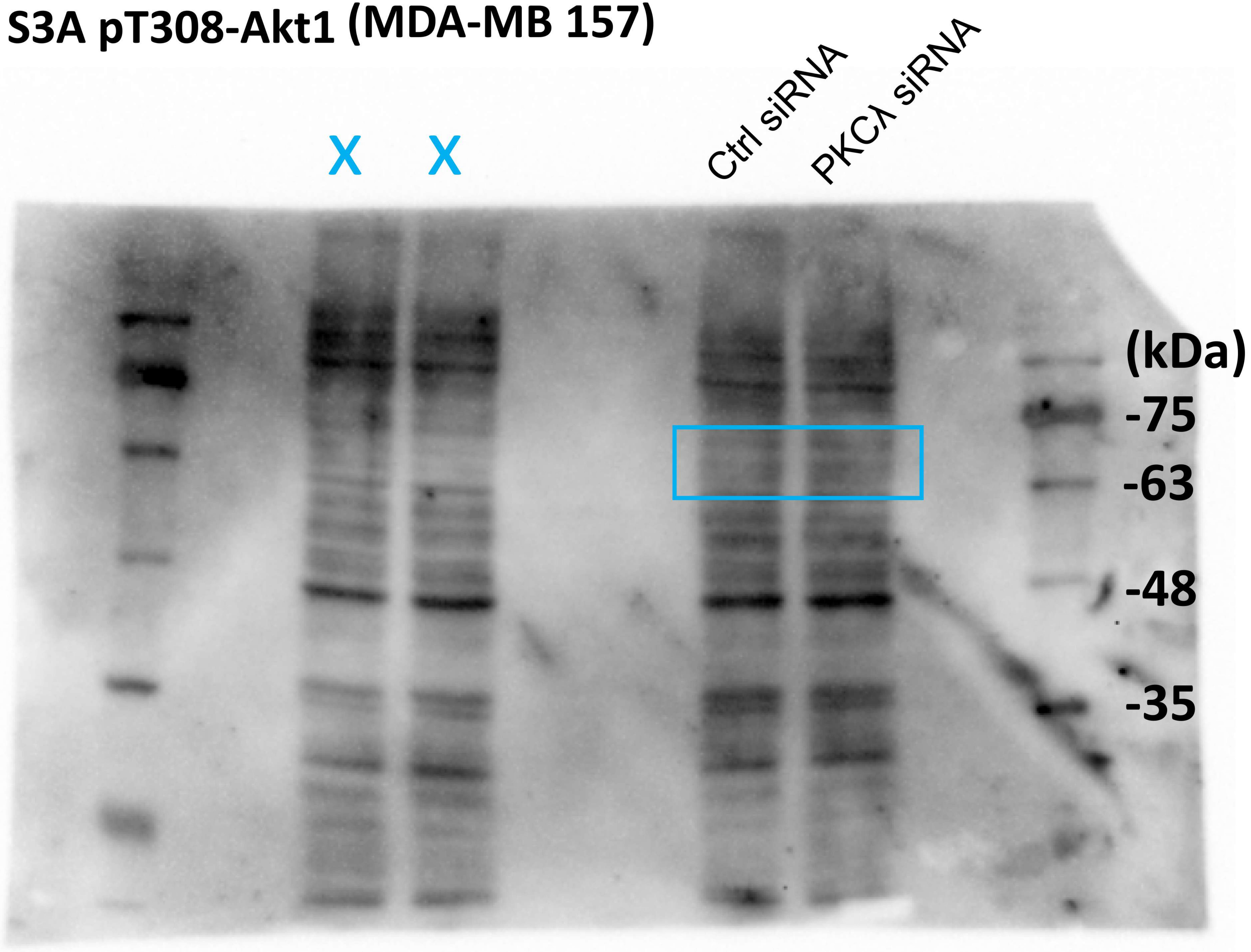

Figure S3A Akt1 (MDA-MB 157)

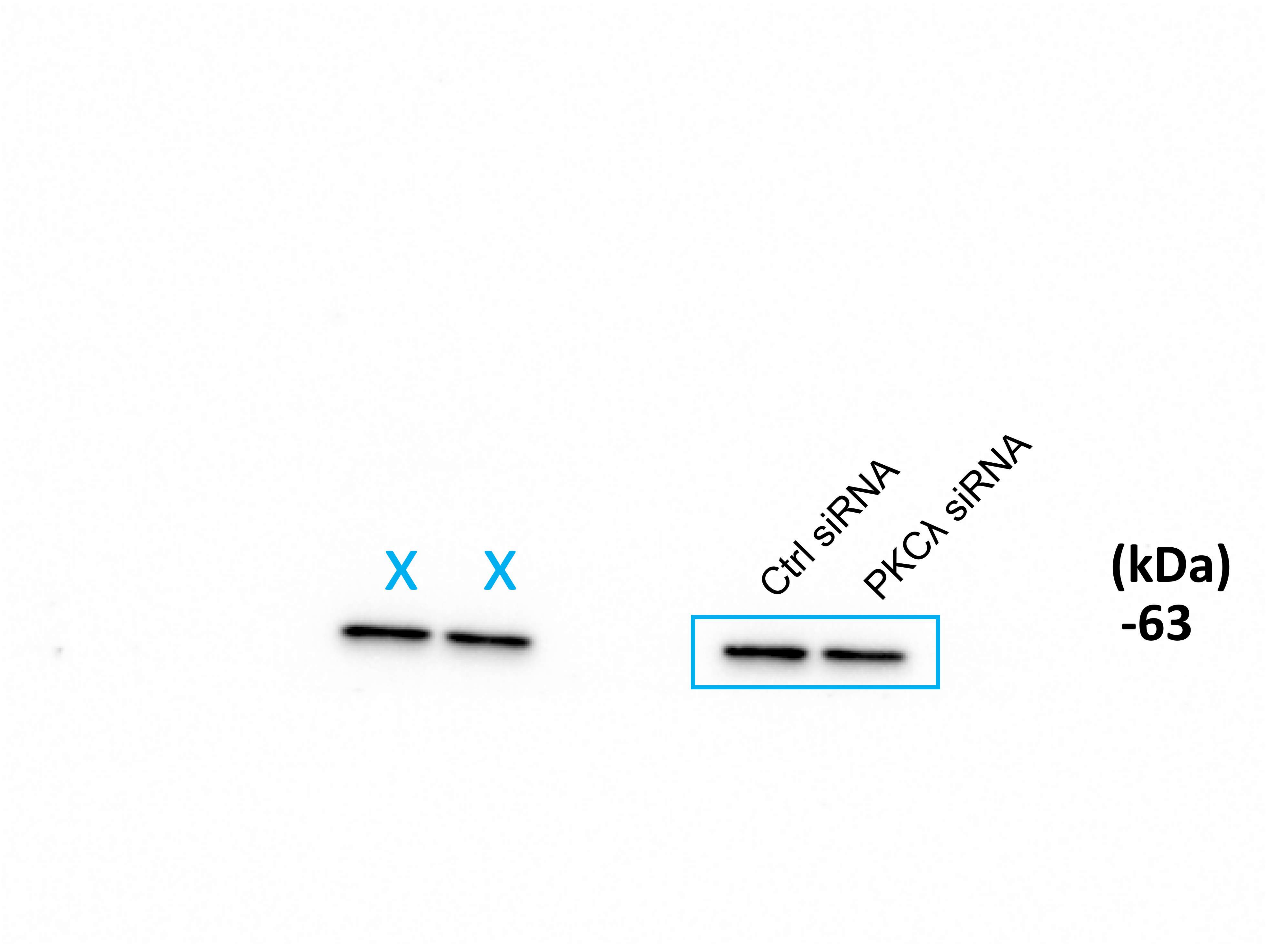

Figure S3A Phospho-p44/42 MAPK (MDA-MB 157)

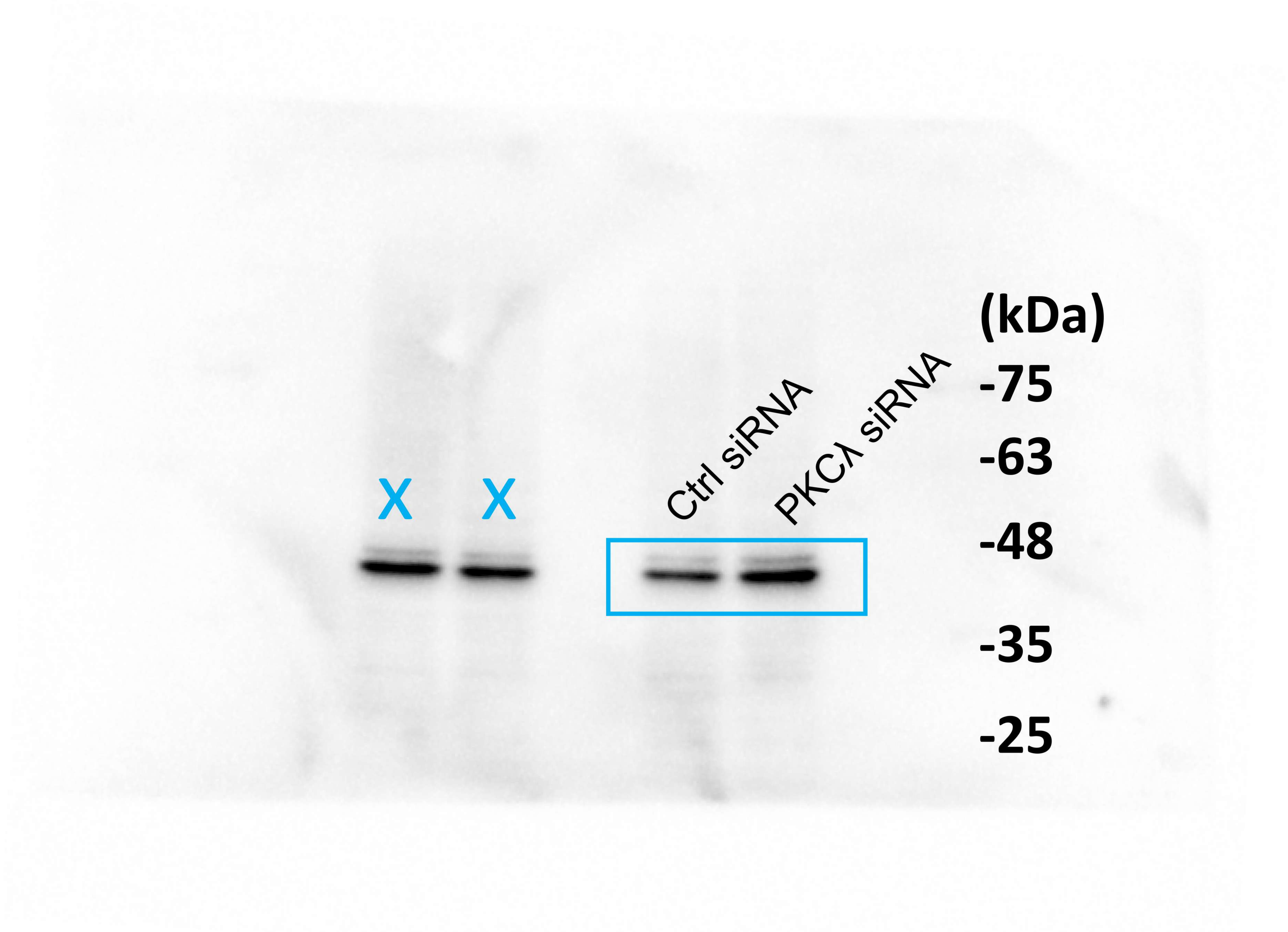

Figure S3A p44/42 MAPK (MDA-MB 157)

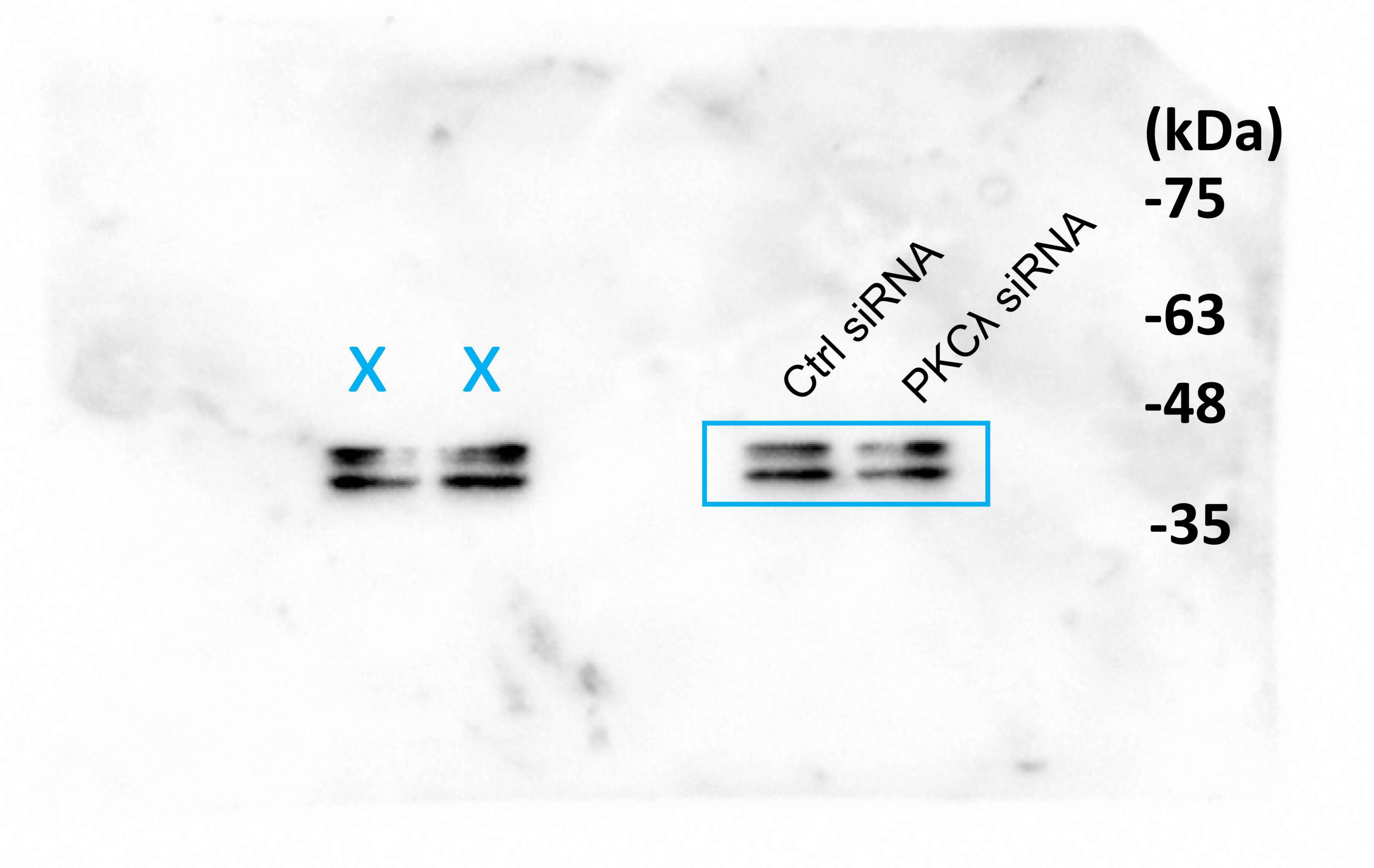

Figure S3A PKCλ (MDA-MB 157)

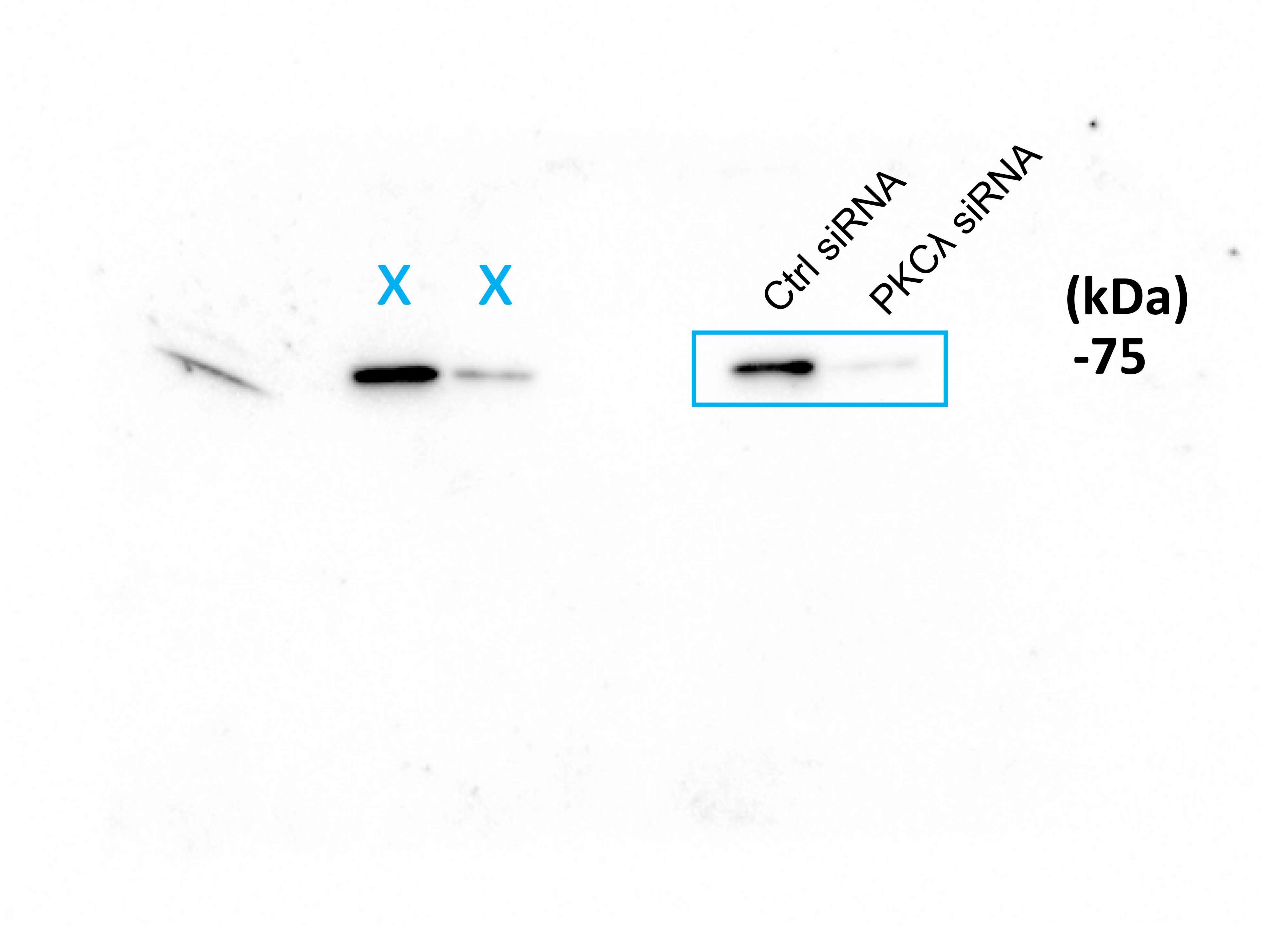

Figure S3A β-actin (MDA-MB 157)

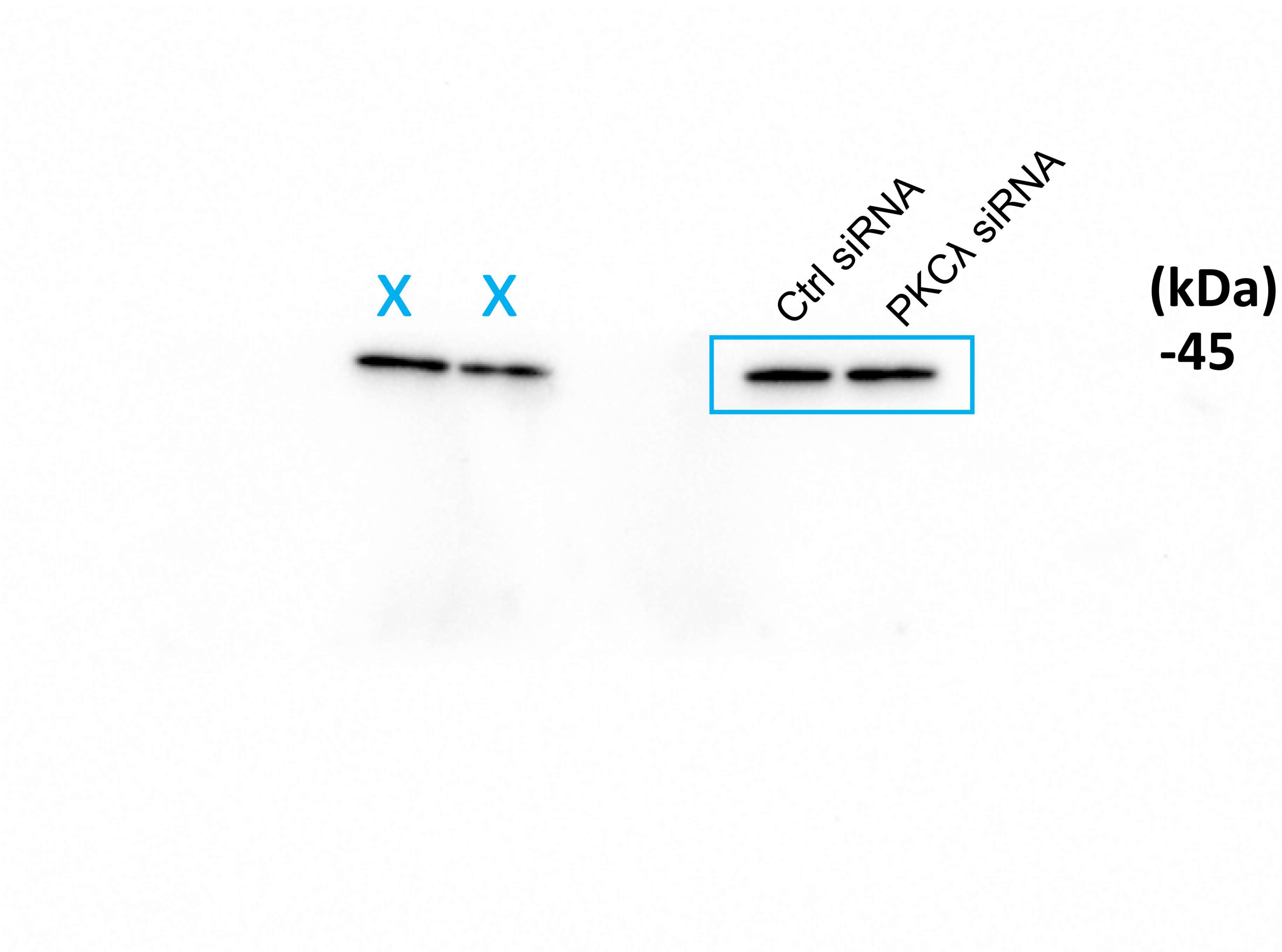

Figure S3A pS473-Akt1 (MDA-MB 468)

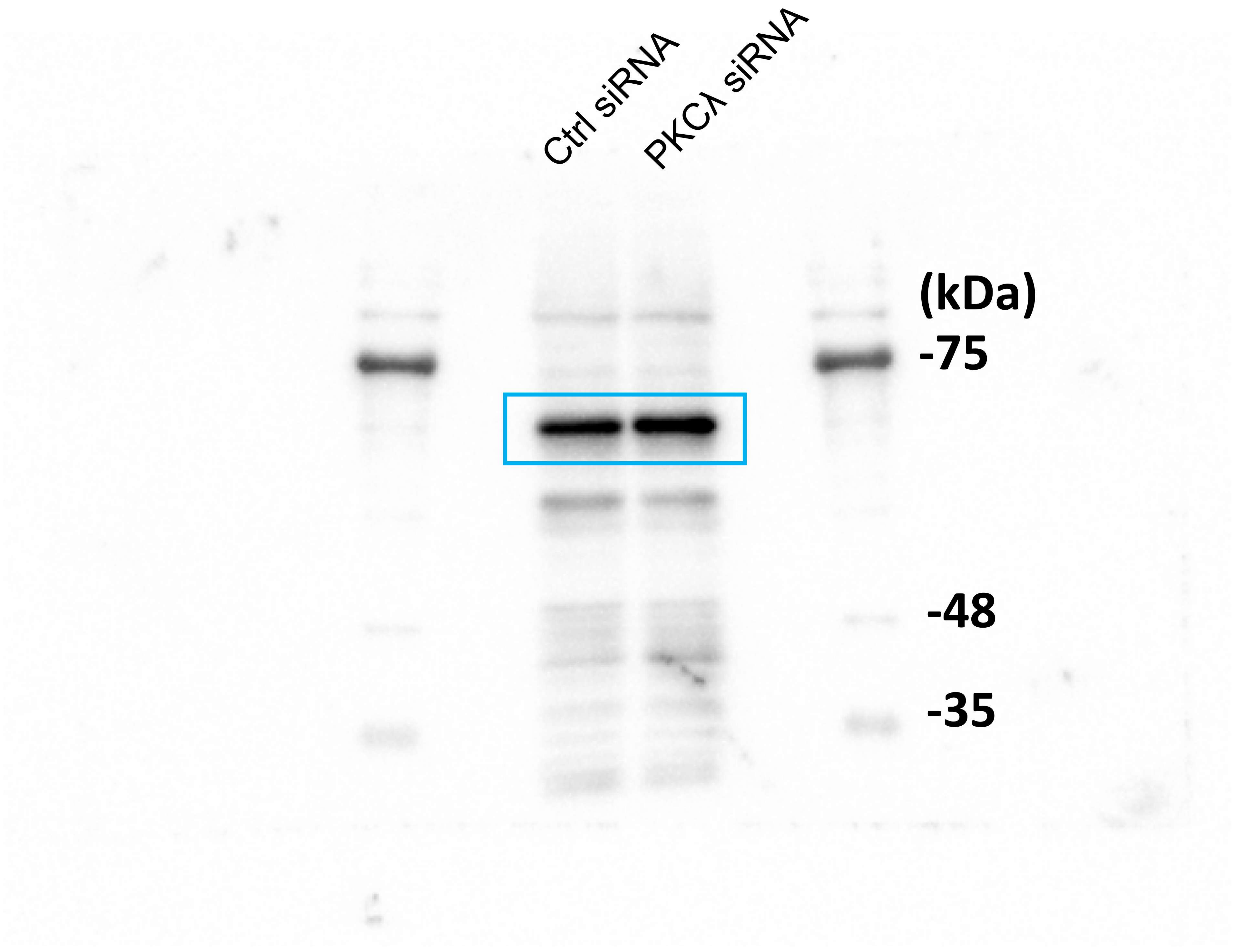

Figure S3A pT308-Akt1 (MDA-MB 468)

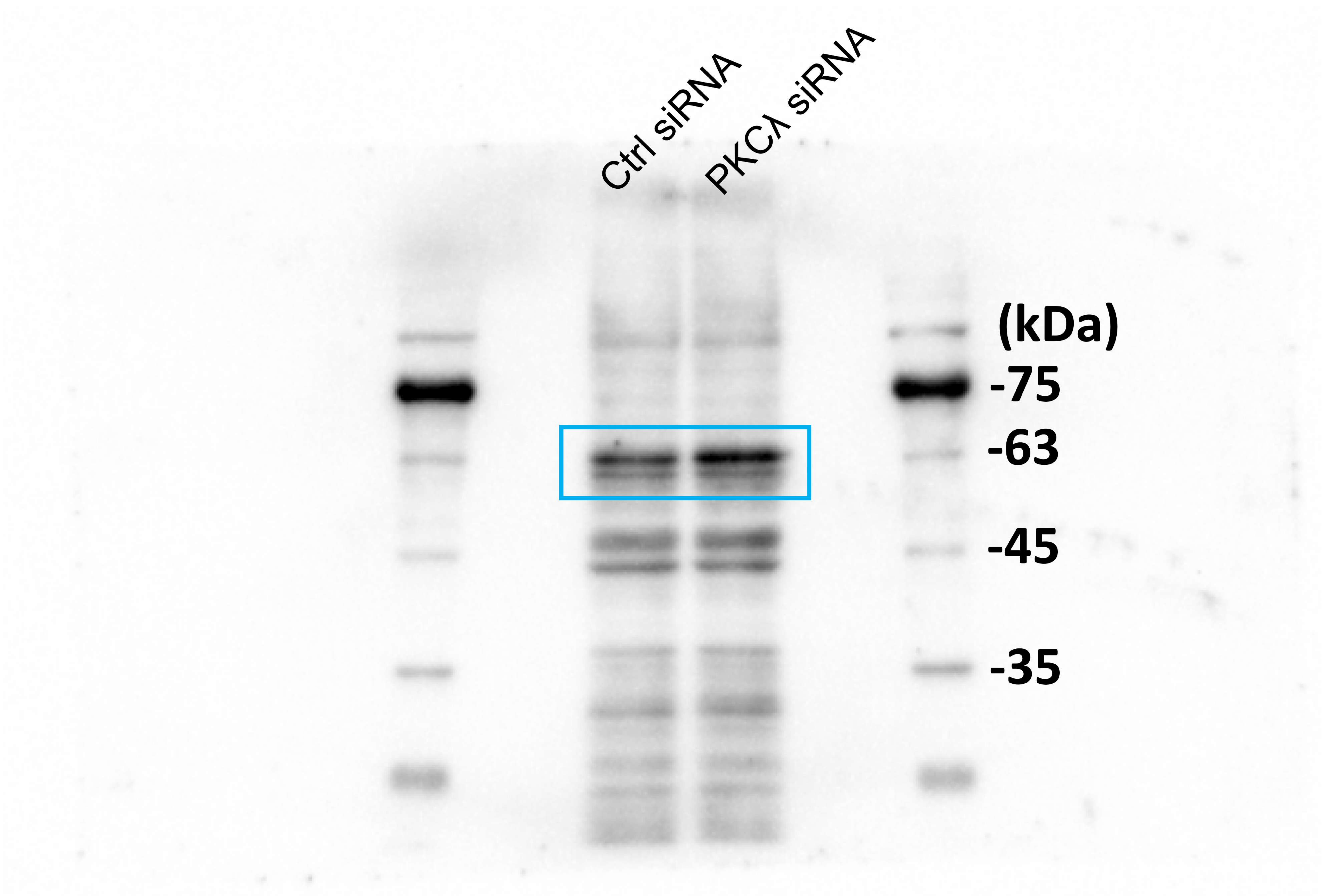

Figure S3A Akt1 (MDA-MB 468)

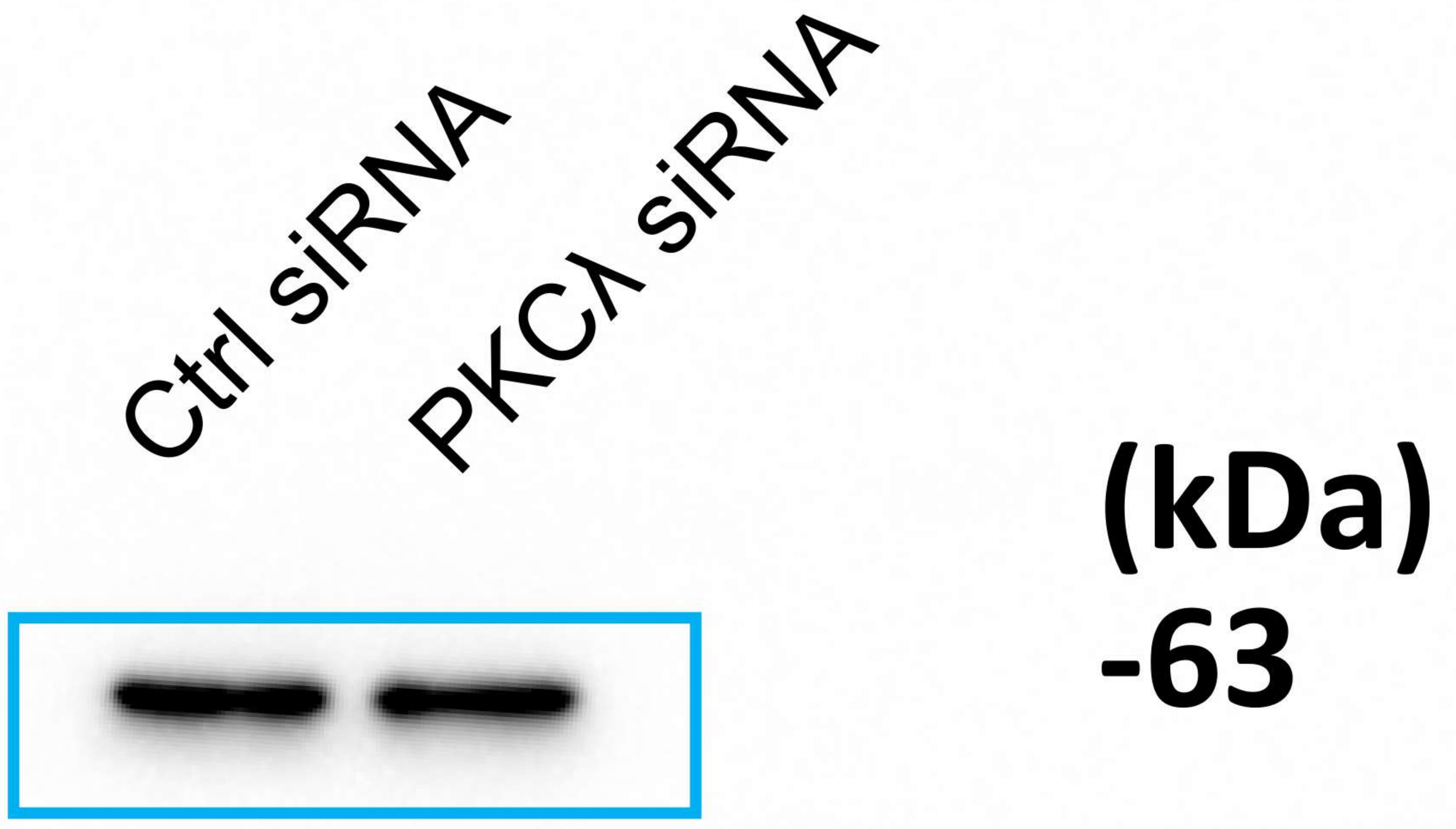

Figure S3A Phospho-p44/42 MAPK (MDA-MB 468)

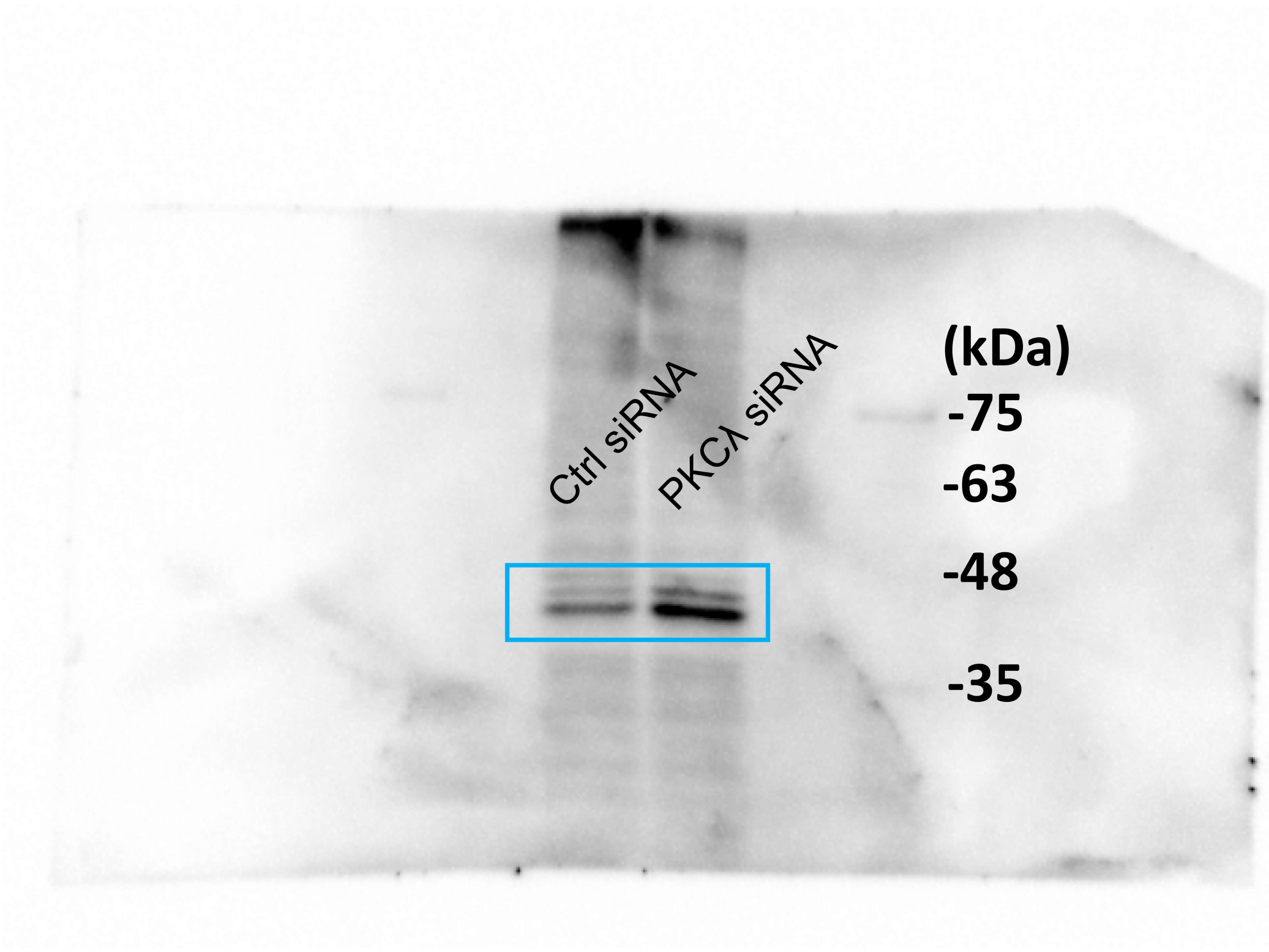

Figure S3A p44/42 MAPK (MDA-MB 468)

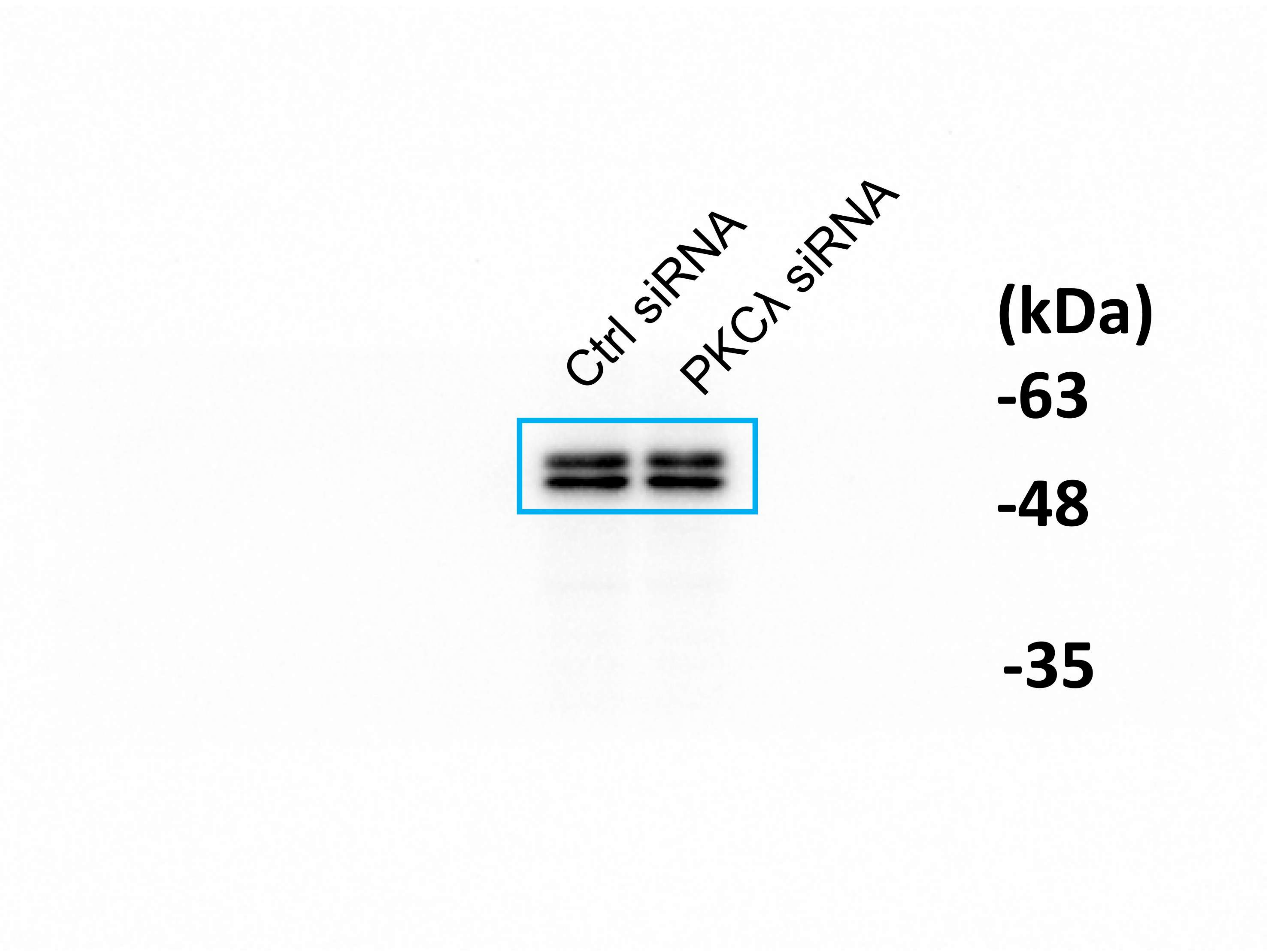

Figure S3A PKCλ (MDA-MB 468)

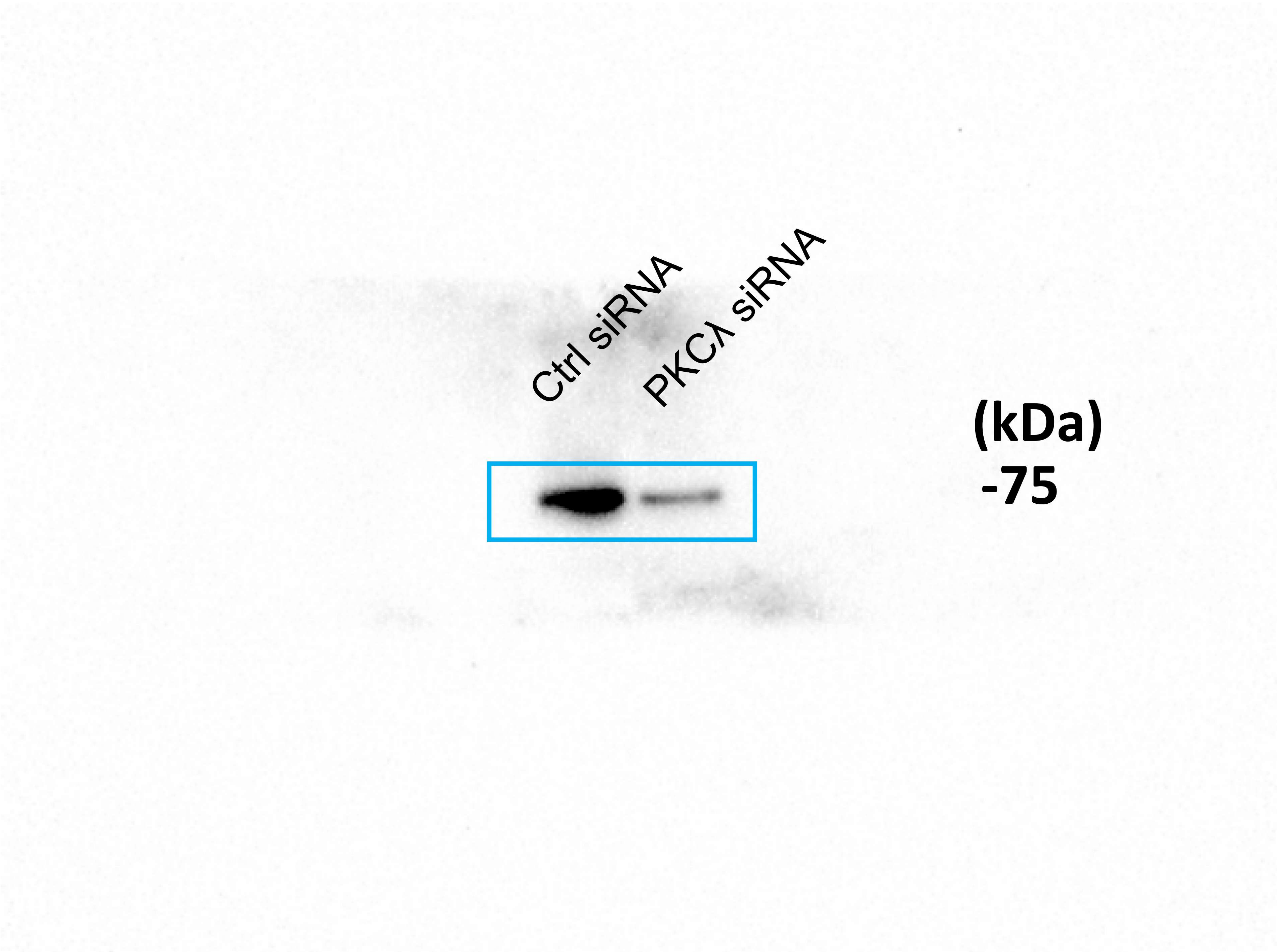

Figure S3A β-actin (MDA-MB 468)

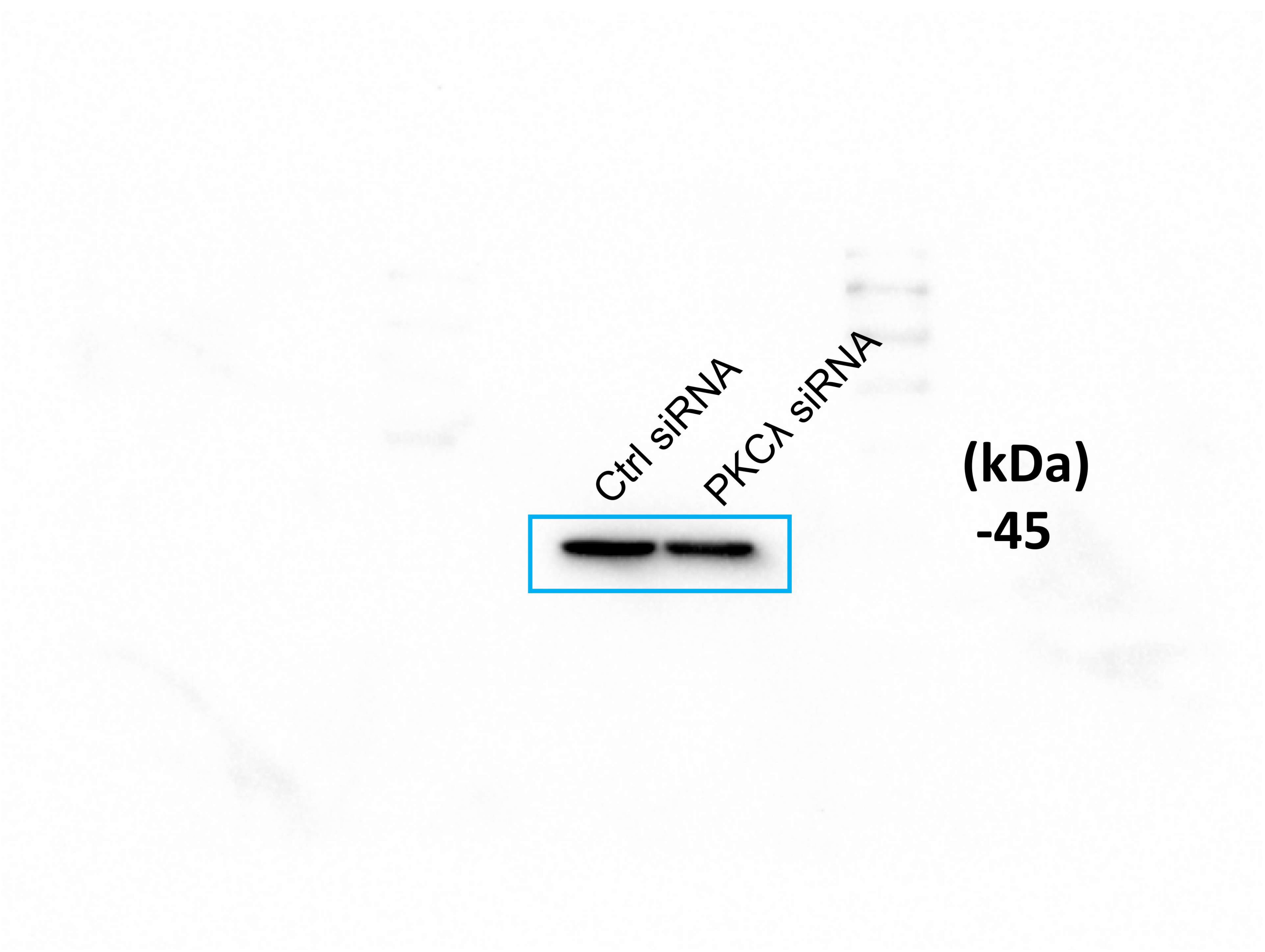

Figure S3B pS473-Akt1

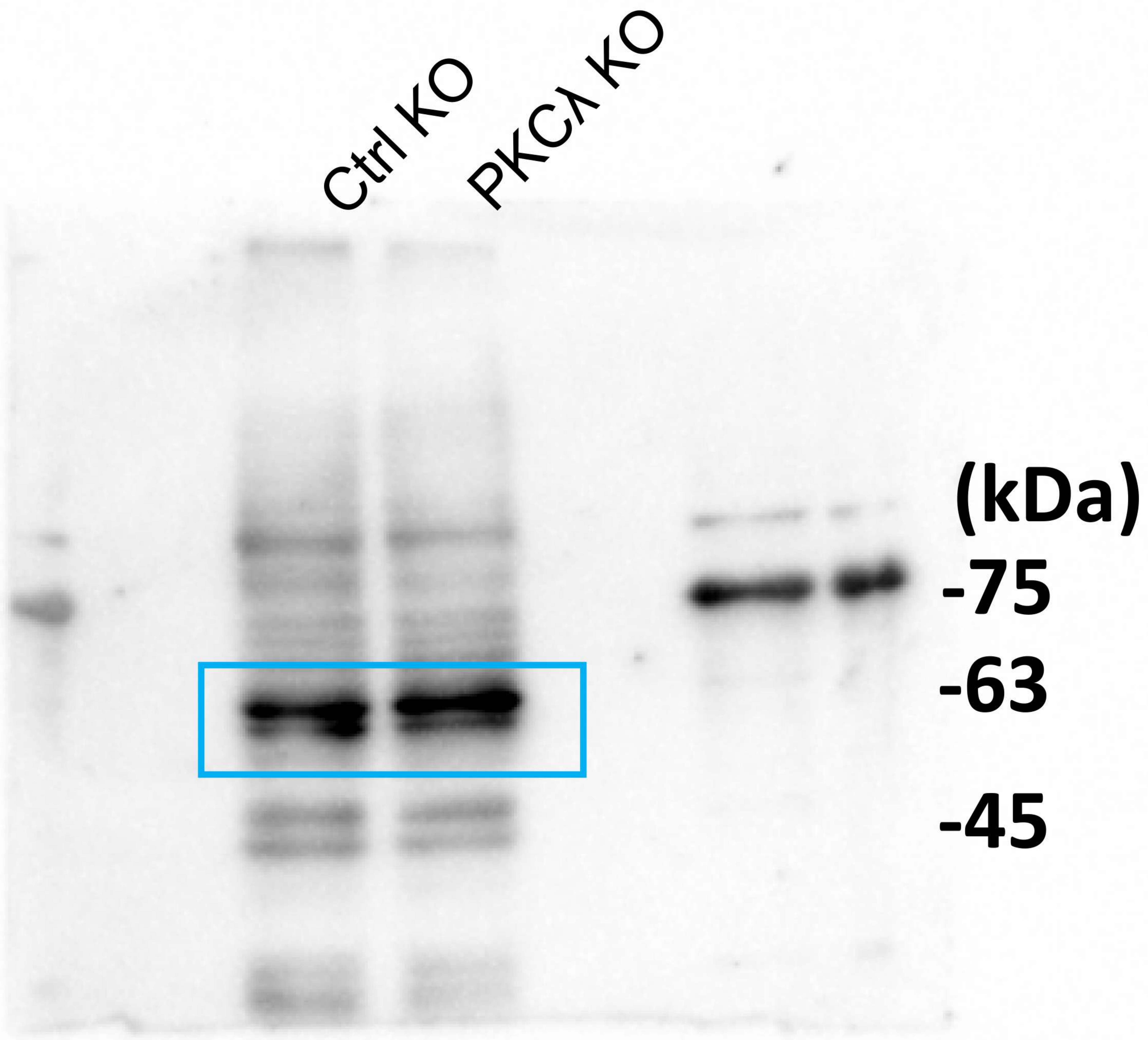

Figure S3B pT308-Akt1

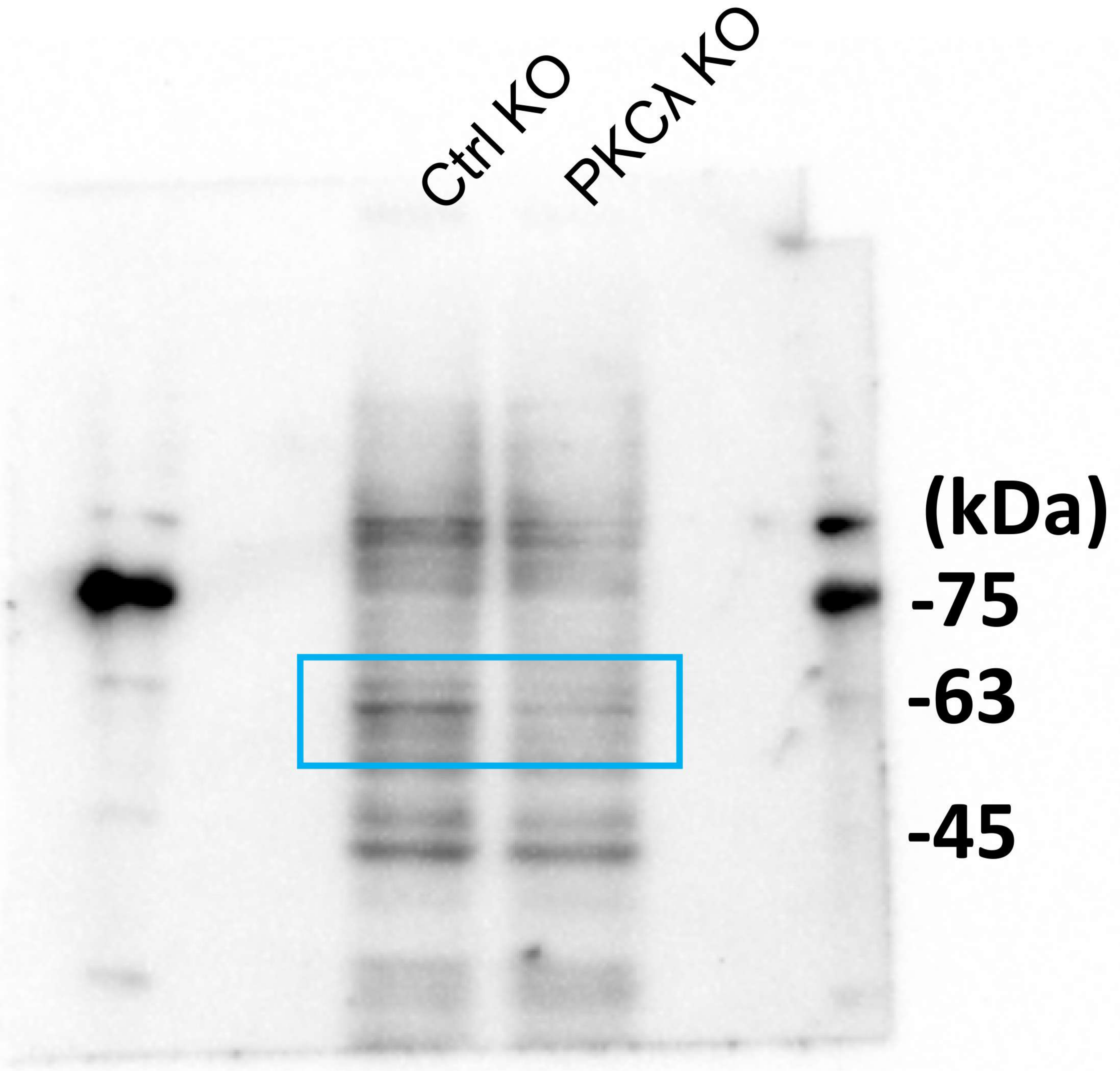

Figure S3B Akt1

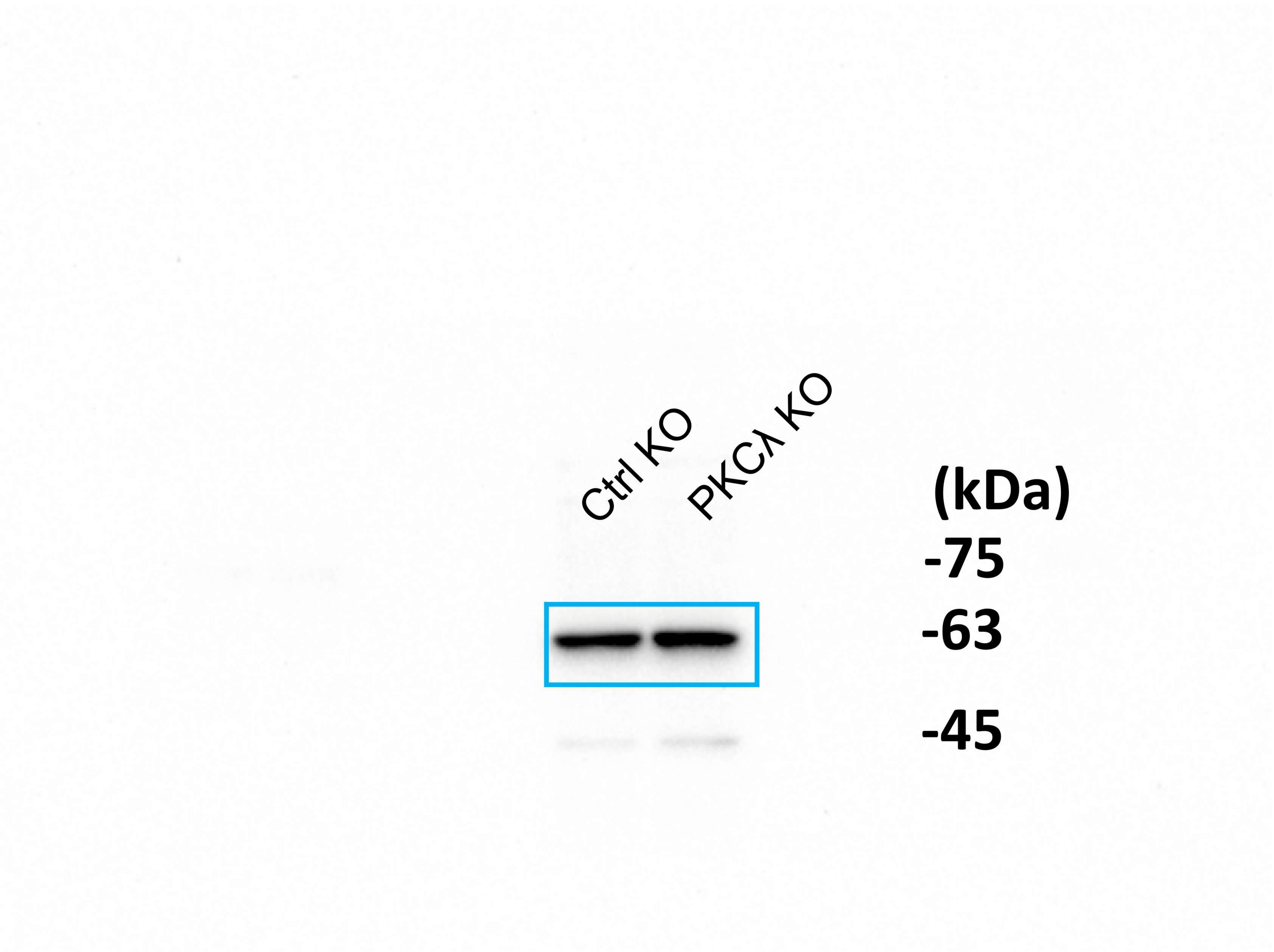

Figure S3B β-actin

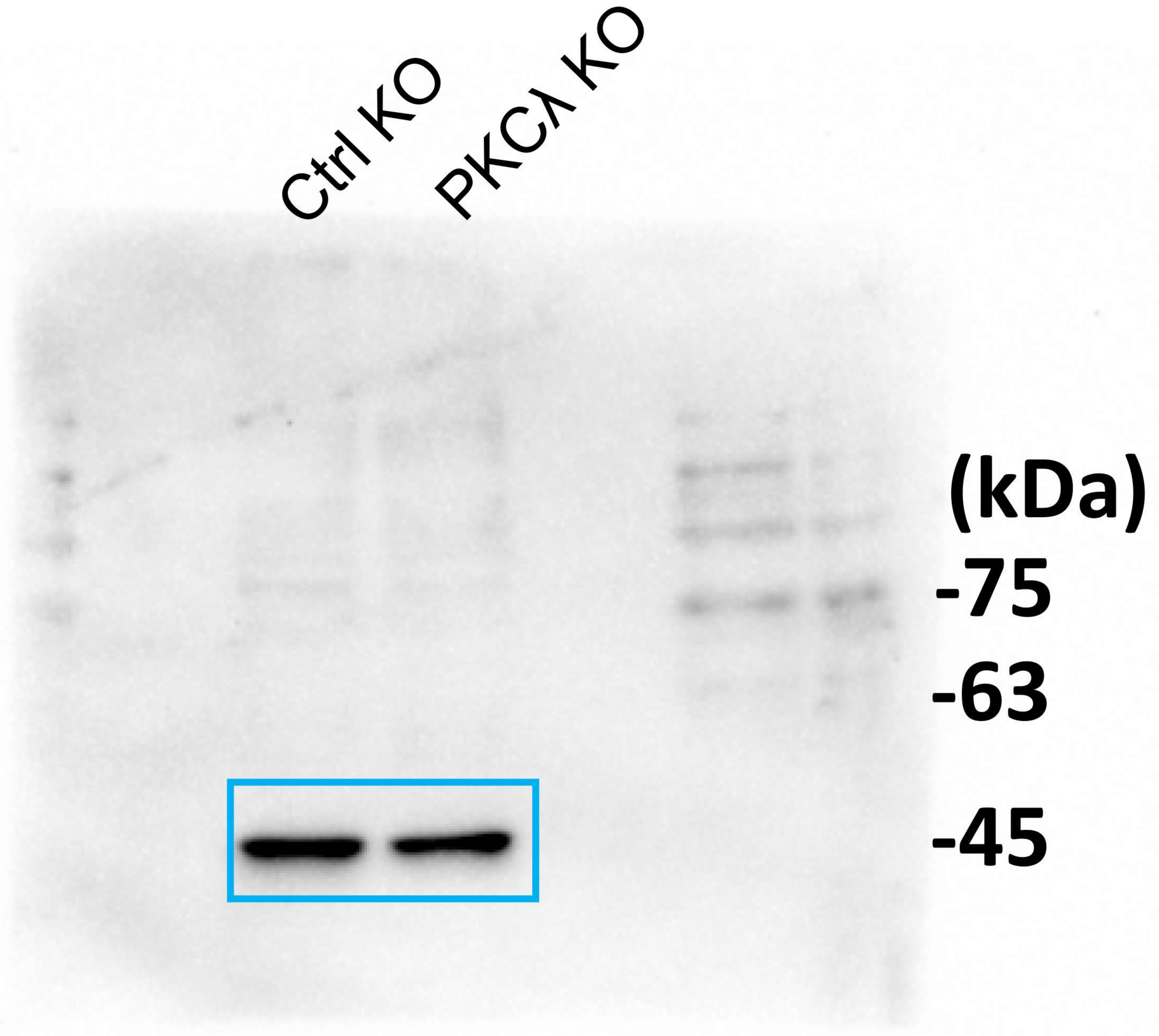

Figure S3B p-p44/42 MAPK

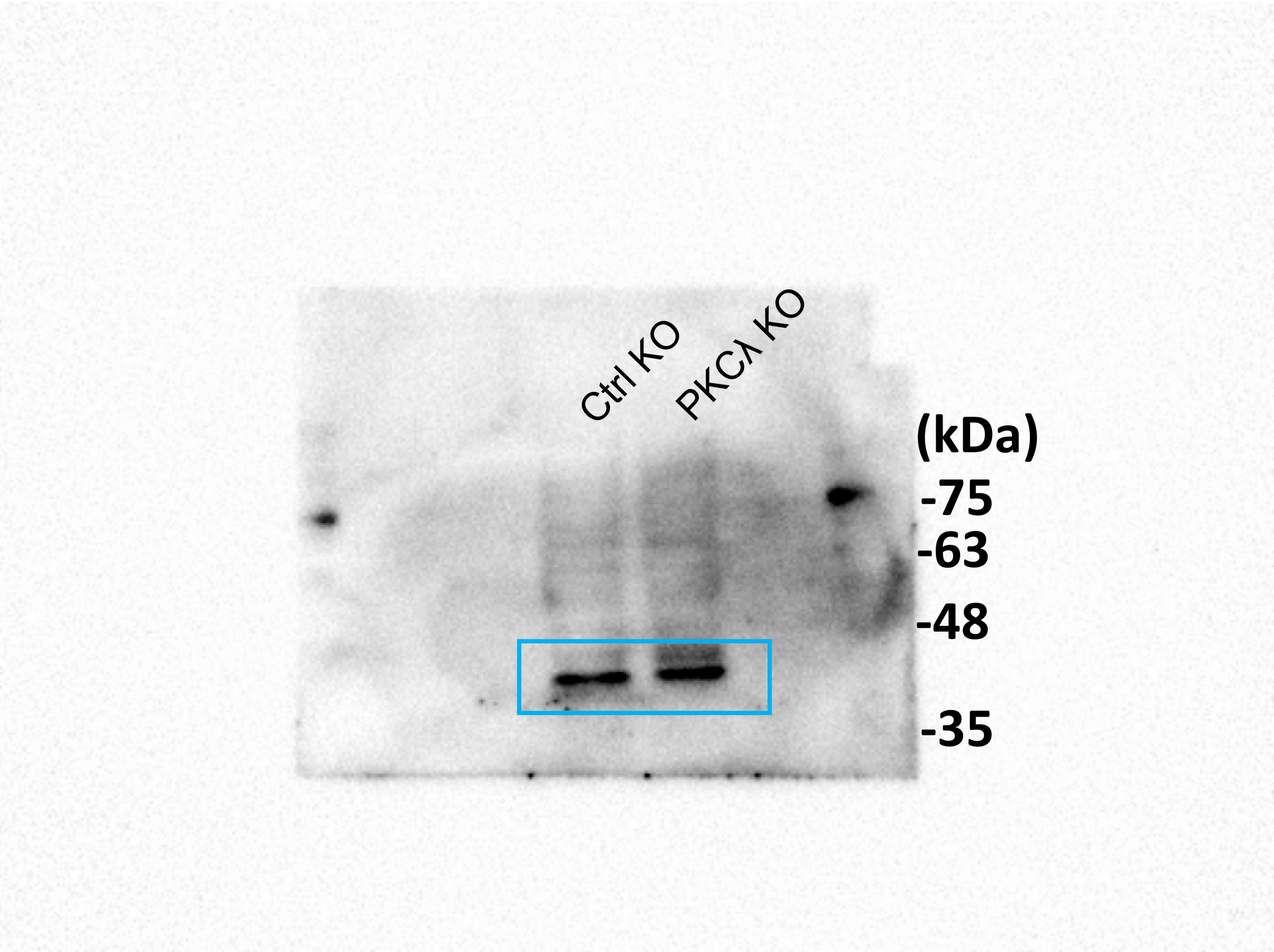

Figure S3B p44/42 MAPK

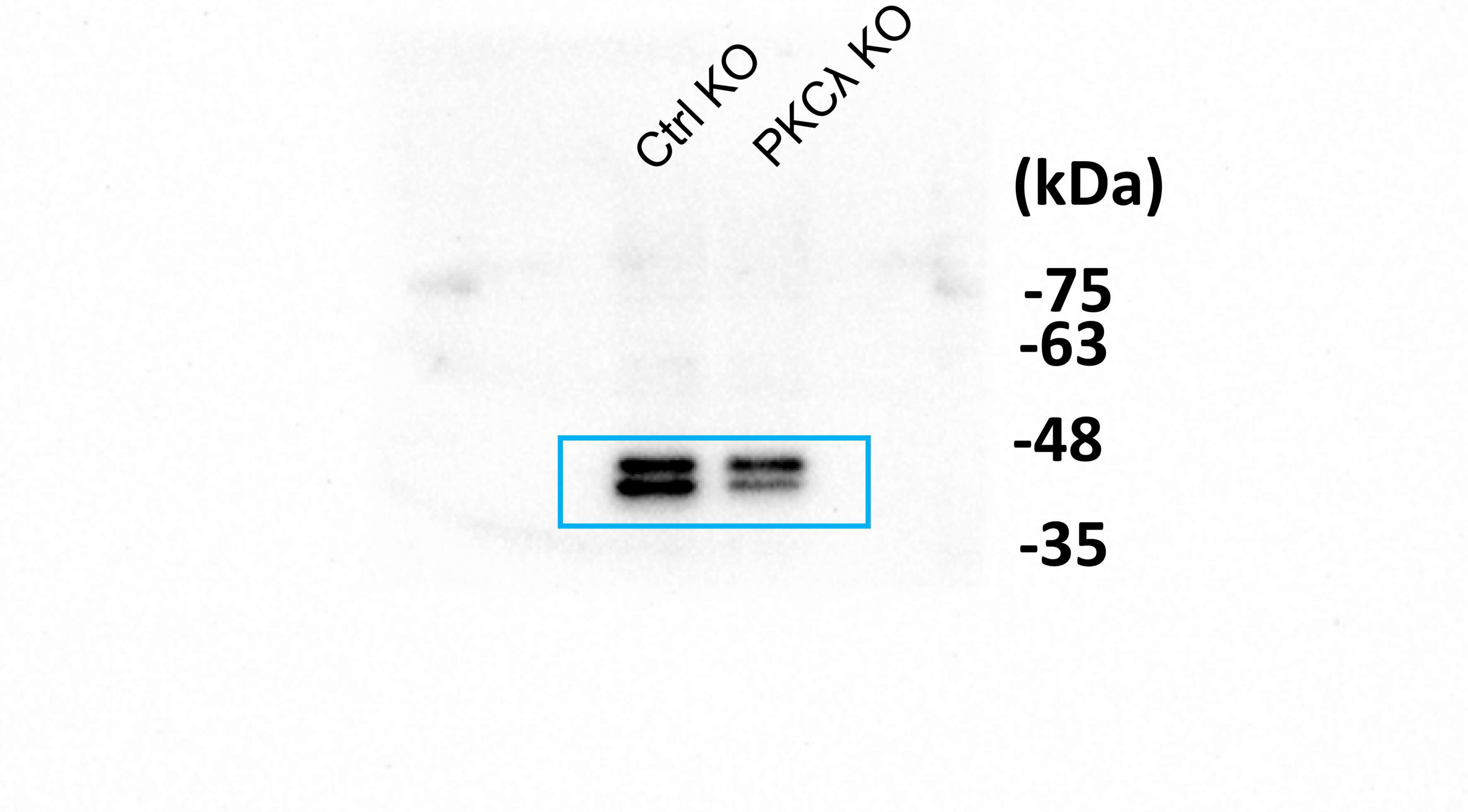

Figure S3B β-actin

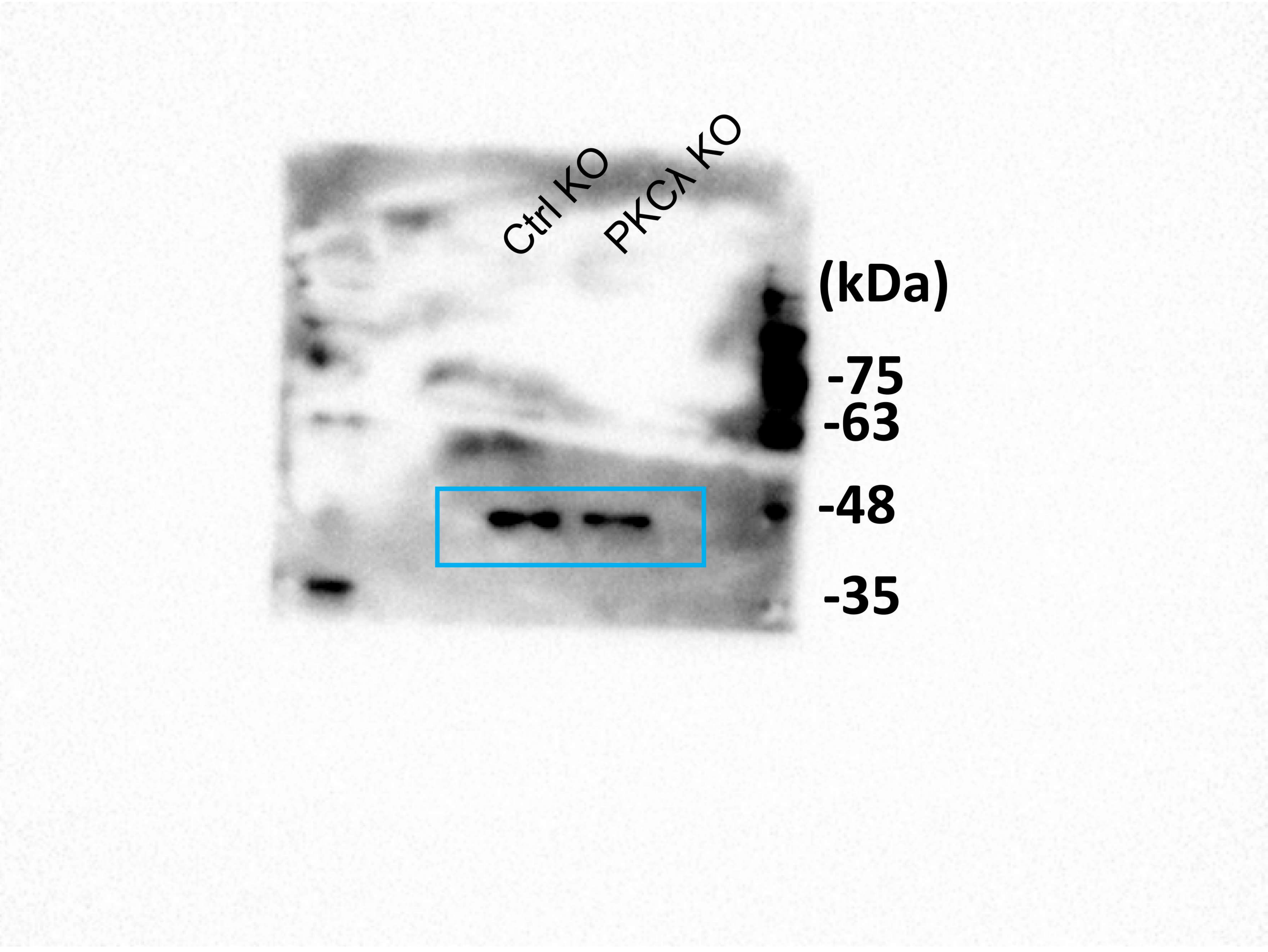

Supplement: S1 Raw images — (PDF) [file pone.0235747.s009.pdf]
